# Divergent trends and regional disparities in PM_2.5_ and O_3_ health economic burdens in China, 2013–2023: an integrated assessment with policy implications

**DOI:** 10.3389/fpubh.2025.1683415

**Published:** 2025-11-13

**Authors:** Ziqi Tang, Xialing Sun, Shichao Zhu, Chongbao Ren, Xiaotong Qie, Jiaxi Wu

**Affiliations:** 1 School of Public Health, Shandong Second Medical University, Weifang, China; 2 School of Mining and Geomatics Engineering, Hebei University of Engineering, Handan, China; 3 China Special Equipment Inspection and Research Institute, Beijing, China; 4 School of Humanities and Social Sciences, Beijing Institute of Petrochemical Technology, Beijing, China; 5 School of Economics and Management, Beijing Institute of Petrochemical Technology, Beijing, China

**Keywords:** PM_2.5_, O_3_, health economic burdens, spatial autocorrelation, China

## Abstract

Utilizing China’s air quality monitoring data from 2013 to 2023, this study employs spatial autocorrelation analysis and health impact assessment methodologies to quantify the health economic costs and temporal trends of PM_2.5_ and O_3_ pollution across mainland China. Results indicate a substantial decline in PM_2.5_-attributable mortality over the study period. With 0 and 15 μg/m^3^ taken as the reference concentrations, all-cause mortality decreased by 47.41% (233,173 cases) and 65.55% (240,448 cases), respectively. Cardiovascular disease mortality declined by 47.71% (56,086 cases) and 65.75% (57,504 cases), while respiratory disease mortality reduced by 46.97% (38,519 cases) and 65.27% (40,055 cases). Conversely, O_3_-related mortality exhibited a significant upward trend. At reference concentrations of 0 and 60 μg/m^3^, all-cause mortality increased by 24.25% (365,084 cases) and 79.70% (372,724 cases), respectively. Cardiovascular mortality rose by 25.43% (80,056 cases) and 81.52% (77,497 cases), and respiratory mortality increased by 15.46% (98,620 cases) and 64.54% (163,165 cases). Spatially, health economic costs and their GDP proportions for both pollutants followed a high distribution in the east and a low distribution in the west. Shandong, Henan and Jiangsu were the top regions for PM_2.5_-related economic costs, with Henan, Hebei and Tianjin exhibiting the highest GDP ratios. For O_3_, Guangdong, Jiangsu and Shandong incurred the greatest economic costs, while Henan, Hebei and Gansu showed the highest GDP proportions. These findings underscore the divergent trends in PM_2.5_ and O_3_ health impacts and provide critical evidence for targeted air pollution control strategies in China.

## 1 Introduction

Air pollution remains a paramount global public health challenge, with fine particulate matter (PM_2.5_) and ground-level ozone (O_3_) emerging as key contributors to premature mortality and morbidity, particularly in rapidly industrializing nations like China (1). Rapid economic growth and urbanization in China have intensified these issues, exacerbating ecological degradation and environmental risks. Since the implementation of stringent air quality controls in 2013, PM_2.5_ concentrations have declined substantially, yet O_3_ levels have increased in both intensity and duration, leading to a shift where short-term O_3_ exposures now account for more premature deaths than PM_2.5_ in certain seasons (2–4).

Epidemiological evidence underscores the distinct yet severe health hazards posed by each pollutant. PM_2.5_ exposure is associated with elevated risks of respiratory diseases (e.g., chronic obstructive pulmonary disease and asthma exacerbations) and cardiovascular conditions (e.g., ischemic heart disease and stroke), driven by mechanisms such as systemic inflammation, oxidative stress, and endothelial dysfunction (5–7). In contrast, O_3_, a highly reactive oxidant, primarily affects the respiratory system by inducing airway inflammation and reduced lung function, while also contributing to cardiovascular mortality (8–10). O_3_ and PM_2.5_ pollution disproportionately burdens developing countries like China and India, where rising concentrations pose escalating threats to vulnerable populations (4, 11). According to China’s Blue Book on Atmospheric Ozone Pollution Prevention and Control (63), developing effective O_3_ control strategies is crucial to mitigate its rising trends, enable synergistic management with PM_2.5_, improve air quality, and advance the Beautiful China initiative, underscoring the need for integrated health impact research across multiple policy phases.

Despite these concerns, existing studies on PM_2.5_ and O_3_ have predominantly focused on short-term analyses or specific regions, such as the Beijing-Tianjin-Hebei (BTH) and Yangtze River Delta (YRD) urban clusters (12, 13), limiting insights into long-term national trends. For instance, the study employed integrated models (GAINS, GEOS-Chem, and CGE) to assess national health and economic impacts but emphasized future scenarios rather than historical co-evolution (14). Similarly, another study quantified city-level burdens from 2013 to 2020, revealing a ~ 48% reduction in PM_2.5_ exposure alongside worsening O_3_ pollution, yet overlooked broader spatial autocorrelations and demographic vulnerabilities (4). Another analysis examined premature mortality trends from 2005 to 2017, highlighting health disparities attributable to PM_2.5_, but did not fully integrate O_3_ dynamics or economic costs at a provincial scale (15). While these works provide valuable regional or temporal insights, they rarely address the co-evolutionary trends of PM_2.5_ and O_3_ in relation to socioeconomic factors like urbanization that exacerbate exposure inequalities. Likewise, another study analyzed spatiotemporal trends in disease burden from 2005 to 2017, highlighting reductions in PM_2.5_ impacts but rising O_3_ burdens, with a focus on national temporal evolution and limited policy integration beyond general air quality improvements. While post-2013 policies—such as the Air Pollution Prevention and Control Action Plan and the Three-Year Action Plan for Winning the Blue Sky Defense Battle (2018–2020)—have driven PM_2.5_ reductions, O_3_ levels have paradoxically risen due to shared precursors (e.g., volatile organic compounds and nitrogen oxides), where PM_2.5_ mitigation reduces aerosol scavenging of hydroperoxyl radicals (HO_2_), thereby enhancing O_3_ formation (16–19). This synergistic interaction underscores a critical research gap: few studies have examined decadal, nationwide spatiotemporal dynamics (20), including spatial disparities, demographic vulnerabilities, and policy-driven co-evolution across all 31 provinces, particularly in relation to evolving vulnerability factors like urbanization and socioeconomic inequalities.

To address these gaps, this study integrates air quality monitoring data from 2013 to 2023 to quantify the spatiotemporal distributions, autocorrelations, and health economic burdens attributable to PM_2.5_ and O_3_ in mainland China. By employing spatial autocorrelation analysis and health impact assessment models, we delineate interannual variations, identify high-risk hotspots, and evaluate divergent mortality trends and economic costs relative to GDP, while accounting for policy influences. These findings provide evidence-based insights for targeted, synergistic pollution control strategies, contributing to air quality management in a policy-relevant context.

## 2 Methodology and data sources

### 2.1 Data sources

This study selects data from 31 provinces across China (excluding Hong Kong, Macao, and Taiwan) spanning 2013–2023. The PM_2.5_ concentrations and O_3_ data for each province are sourced from the National Tibetan Plateau / Third Pole Environment Data Center (21, 22). The study utilizes the ChinaHighPM_2.5_ and ChinaHighO_3_ datasets from the Data Center, which are part of the China High Air Pollutants (CHAP) collection, providing high-resolution, high-quality near-surface air pollutant data for China, with a spatial resolution of 1 km and temporal resolutions of daily, monthly, and yearly (in μg/m^3^). The PM_2.5_ dataset fills spatial gaps in the MODIS MAIAC AOD product using model data, while the O_3_ dataset uses surface solar radiation intensity and air temperature as primary predictors. Both integrate ground-based observations, atmospheric reanalysis, and emission inventories (23–25). Ten-fold cross-validation yields *R*^2^ values of 0.92 and 0.89, with RMSEs of 10.76 μg/m^3^ and 15.77 μg/m^3^ for PM_2.5_ and O_3_, respectively, covering the entire Chinese region, and the data coordinate system is WGS-1984 (23, 24). Data on GDP and baseline mortality rates at both national and provincial levels from 2013 to 2023 are obtained from the national and local statistical yearbooks. Provincial-level mortality rates due to cardiovascular and cerebrovascular diseases are derived from the publicly available Global Burden of Disease (GBD) database, while data on mortality rates from respiratory diseases are retrieved from the China Cause of Death Surveillance Dataset (2013–2023).

### 2.2 Spatial autocorrelation

This study calculates the Moran’s I index using Equations 1, 2 to conduct a global spatial autocorrelation test.


$$I=\frac{\sum_{i=1}^{n}\sum_{j=1}^{n}w_{\mathrm{ij}}(x_{i}-x)(x_{j}-x)}{S^{2}\sum_{i=1}^{n}\sum_{j=1}^{n}w_{\mathrm{ij}}}$$ (1)

$$S^{2}=\frac{1}{n}\sum_{i=1}^{n}{\left(x_{i}-x\right)}^{2}$$ (2)

In Equations 1, 2, I denotes the global Moran’s I index; n represents the total number of areal units within the study area, i, j refer to the *i*th and *j*th areal units, respectively, $w_{\mathrm{ij}}$ is the element value of the spatial weight matrix, $x_{i}$, $x_{j}$ stand for the attribute values of the *i*th and *j*th areal units, x indicates the mean value of attribute values across all areal units, and $S^{2}$ is the variance of attribute values among the areal units. Local spatial autocorrelation analysis was performed using local Moran’s I to examine the local spatial autocorrelation of concentrations. The spatial heterogeneity of the data was characterized through four types of local association patterns: Low–Low (LL), Low–High (LH), High–Low (HL), and High–High (HH). Specifically, the LL and HH patterns indicate positive spatial correlation between neighboring regions, whereas the LH and HL patterns represent negative spatial correlation across adjacent areas. The corresponding formulas are provided as Equations 3–5.


$$I_{i}=\frac{Z_{i}}{S^{2}}\sum_{j\neq i}^{n}w_{\mathrm{ij}}Z_{j}$$ (3)

$$Z_{i}=x_{i}-x$$ (4)

$$Z_{j}=x_{j}-x$$ (5)

In the above formula, $I_{i}$ represents the local Moran’s index, $Z_{i}$ and $Z_{j}$ are the deviation of the observed value of the *i*th and *j*th spatial unit from the global mean.

### 2.3 Health economic cost effect

Health impact assessment consists of three basic steps: first, assessing the spatial distribution of air quality changes; second, determining the population exposure level; finally, calculating the health impacts of exposure changes using exposure-response relationships (26–28). In this research, a typical linear logarithmic function is used to calculate the health impacts. It is selected for its suitability to China’s national air quality monitoring data (covering 31 provinces since 2013) and alignment with related studies (29, 30), providing a robust linear relationship for PM_2.5_ and O_3_ exposure (2013–2023). Compared to the non-linear Integrated Exposure-Response (IER) function, this model offers a practical balance (28, 31).


$$\mathrm{\Delta Y}=Y_{0}(1-e^{-\mathrm{\beta \Delta Χ}})\times \mathrm{Pop}$$ (6)

In Equation 6 ΔY is the health impact estimate of changes in pollutant concentration, $Y_{0}$ shows the baseline incidence rate of health endpoints (i.e., mortality or morbidity), Pop indicates the population affected by changes in air quality, ΔX refers to the air quality, β means the exposure-response coefficient.

The economic benefits of health impacts are evaluated using Equation 7:


E=ΔY×U (7)

In Equation 7, E represents economic benefits, ΔY shows the estimated health impact of changes in pollutant concentration, and U is the economic loss caused by each health effect endpoint.

The exposure-response coefficient β represents the correlation between changes in pollutant concentration and population health. It is a core element in the quantitative assessment of health impact endpoints (32). To mitigate uncertainty from single β sources, we used combined estimates reported in prior studies to address data source variability (28, 33–36). Sensitivity analyses with these β and 95% CI confirmed result robustness.


β=In(HR)/ΔC (8)

$$\beta_{\min}=\beta -(1.96\times \sigma_{\beta })$$ (9)

$$\beta_{\max}=\beta +(1.96\times \sigma_{\beta })$$ (10)

In Equations 8–10, ΔC shows the change in pollutant concentration, and $\sigma_{\beta }$ is the standard deviation of β.

The exposure response relationship coefficient of PM_2.5_ and O_3_, as well as the baseline incidence rate of each health endpoint, are shown in Tables 1–3.

**Table 1:** Exposure-response relationship coefficient of PM_2.5_.

Health ending	Exposure-response coefficient *β* (μg/m^3^, 95%CI)	Source
All-causes mortality	0.000896	(7, 29)
	(−0.0003, 0.00178)	
Respiratory mortality	0.00143	(28, 29)
	(0.00085, 0.00201)	
Cardiovascular mortality	0.00053	(28, 29)
	(0.00015, 0.00090)	

**Table 2:** Exposure-response relationship coefficient of O_3_.

Health ending	Exposure-response coefficient *β* (μg/m^3^, 95%CI)	Source
All-cause mortality	0.00198	(61)
	(0.000995, 0.003922)	
Respiratory mortality	0.000995	(62)
	(0,0.00198)	
Cardiovascular mortality	0.011333	(61)
	(0.007696, 0.014842)	

**Table 3:** Baseline incidence rate of each health endpoint.

Health ending	Baseline incidence rate $Y_{0}$
All-cause mortality	0.00713
Respiratory mortality	0.0007572
Cardiovascular mortality	0.0028481

In this study, the economic value of health effects is quantified using two approaches: willingness to pay (WTP) and adjusted human capital (AHC). In 2012, Beijing’s WTP for reducing mortality risks attributable to air pollution: Specifically, the health economic cost accounting for PM_2.5_ and O_3_ pollution was estimated at $232,000, with the AHC standing at $132,000 (29, 37, 38). Based on this, the present study adjusts these values using the purchasing power parity (PPP) method. Incorporating data on personal disposable income across different years and provinces, the economic value of unit health loss for each province in China from 2013 to 2019 is derived via Equation 11.


$$U_{i,j}=U_{\mathrm{BJ},2012}\times {\left(\frac{\text{Incom}e_{i,j}}{\text{Incom}e_{\mathrm{BJ},2012}}\right)}^{e}$$ (11)

In Equation 11, $U_{i,j}$ denotes the economic value of per capita health loss in province i during year j, $U_{\mathrm{BJ},2012}$ refers to the economic value of per capita health loss in Beijing in 2012. $\text{Incom}e_{i,j}$ represents the personal disposable income in province i during year j, while $\text{Incom}e_{\mathrm{BJ},2012}$ stands for the personal disposable income in Beijing in 2012, the data sourced from the *China Statistical Yearbook*. e denotes the income elasticity coefficient, for which the value of 0.8 recommended by the Organization for Economic Co-operation and Development (OECD) is adopted in this study (39).

**Figure 1:**
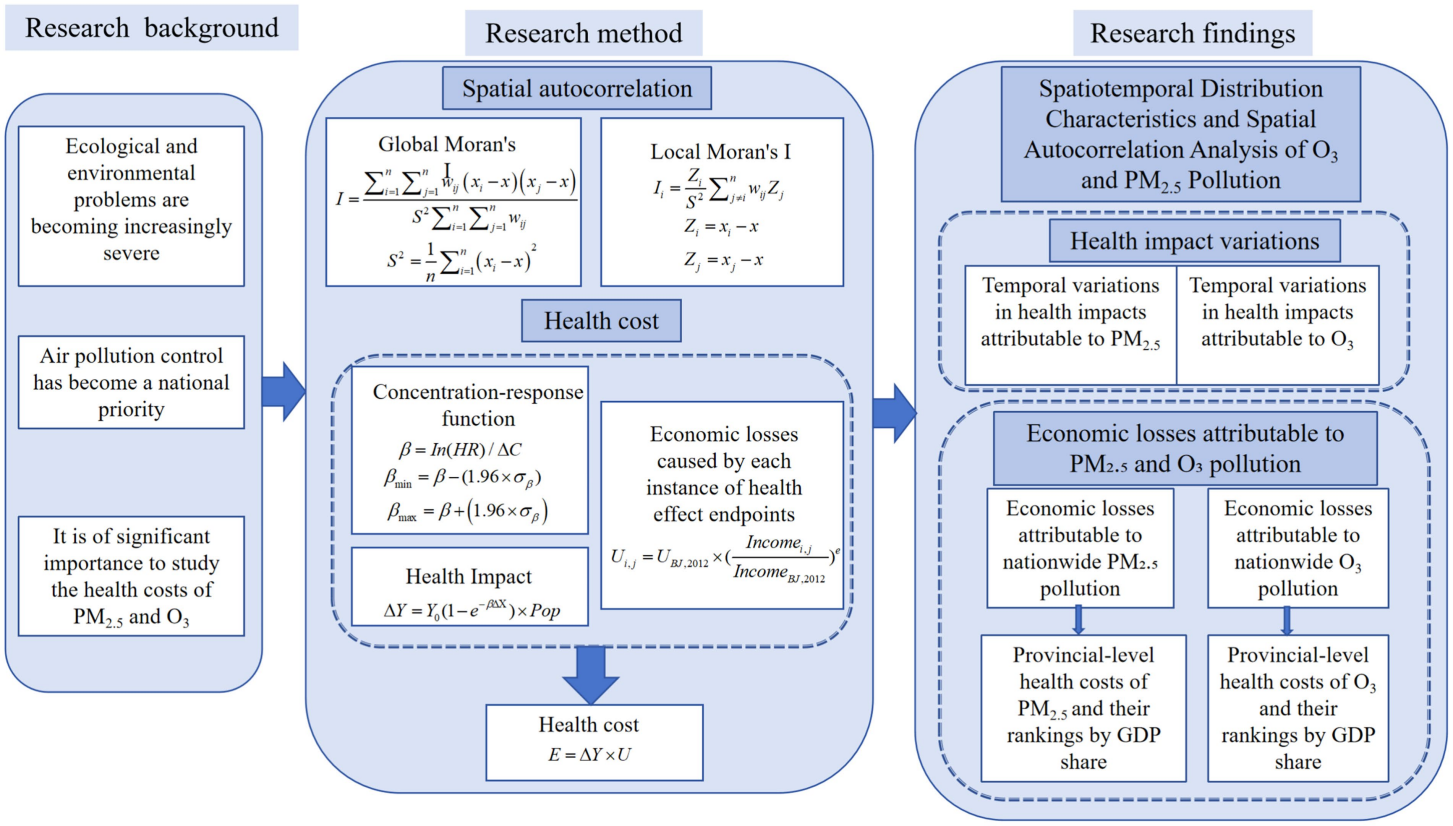
The technical roadmap.

At last, we summarize the technical roadmap in Figure 1.

## 3 Results

### 3.1 Spatial–temporal distribution characteristics and spatial autocorrelation analysis of PM_2.5_ pollution

**Figure 2:**
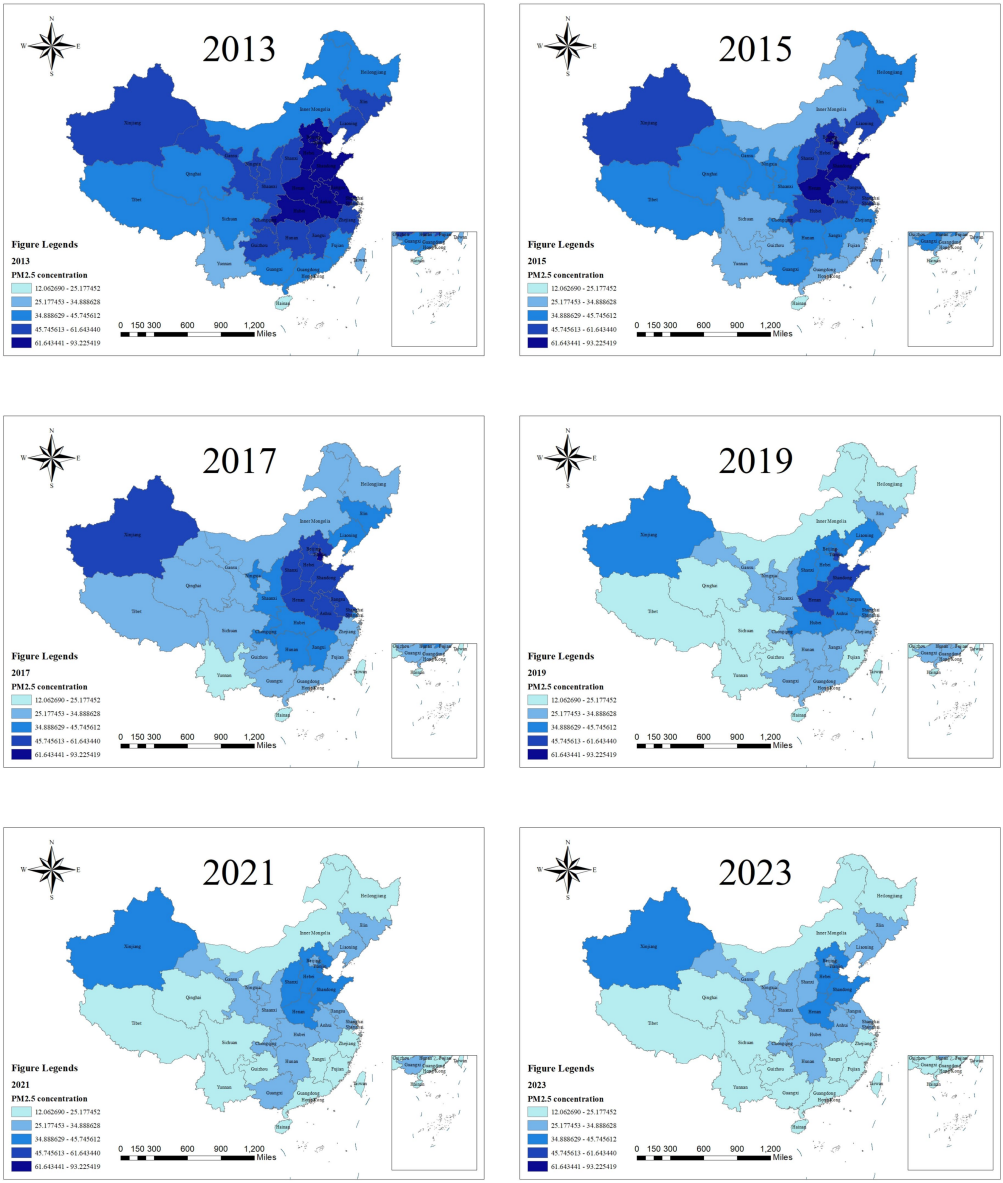
Schematic of PM_2.5_ concentration from 2013 to 2023. Owing to space limitations, not all figures are included in the main text. High-resolution result figures for all study years are available in the Supplementary materials or upon request to the corresponding author, enabling readers to gain a comprehensive understanding of the spatiotemporal evolutionary patterns. In the subsequent text, other figures follow the same processing approach.

As shown in Figure 2, the distribution of PM_2.5_ concentrations in China is characterized by lower levels in the western regions and higher levels in the eastern regions. Specifically, higher PM_2.5_ concentrations are observed in the Beijing–Tianjin–Hebei (BTH) region and its surrounding areas, the Yangtze River Delta (YRD) region, and the Fenwei Plain within the eastern part of the country.

The accelerated development of the BTH region has intensified air pollution, driven by industrial operations, traffic emissions, and urban expansion (40), while PM_2.5_ pollution is also influenced by regional climatic conditions. Cities in BTH that serve as PM_2.5_ transport corridors are categorized into three types: the southwest corridor (from northern Henan via Handan, Shijiazhuang, Baoding to Beijing, contributing ~20% (up to 40% in severe events) to heavy pollution), the southeast corridor (along central Shandong, Cangzhou, Langfang, and southern Tianjin), and the eastern corridor (from Tangshan via northern Tianjin to Beijing). Moreover, meteorological factors such as stagnant weather and high humidity, along with increased winter heating emissions, exacerbate PM_2.5_ accumulation.

In the YRD region, topography and climatic factors (e.g., low wind speeds, frequent temperature inversions) exacerbate PM_2.5_ pollution, compounded by rapid urbanization, dense transport networks, port logistics, and unique geographical conditions (41, 42). In western China, Xinjiang — China’s largest provincial-level region — has high PM_2.5_ concentrations (43). From 2013 to 2016, the overall PM_2.5_ concentration in China showed a significant downward trend, whereas that in Xinjiang exhibited an upward trend. During 2017 to 2019, the national average PM_2.5_ concentration continued to decline steadily. By 2019, basically no regions across the country had PM_2.5_ concentrations that exceeded 70 μg/m^3^.

**Figure 3:**
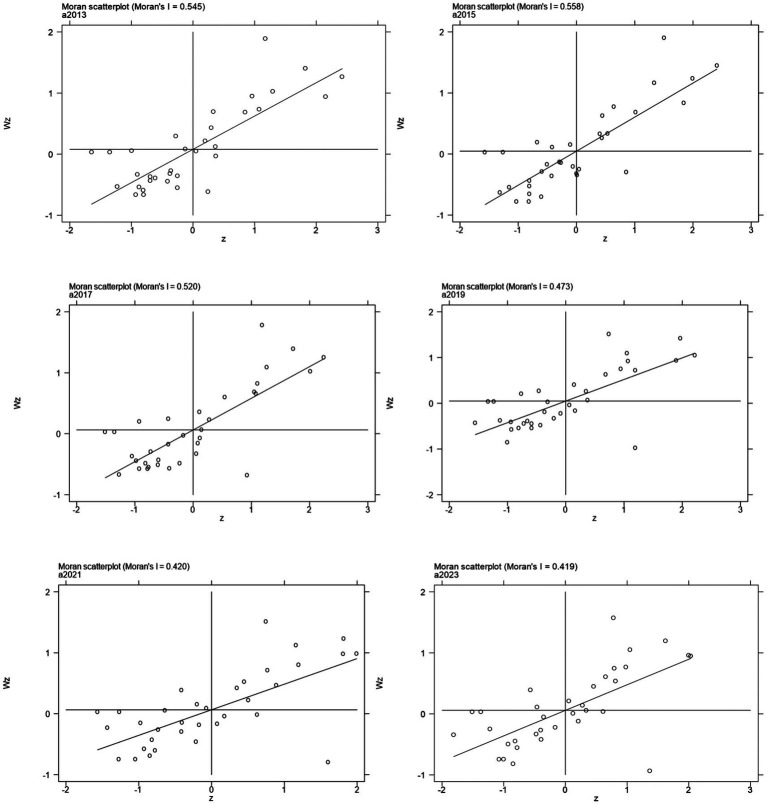
Scatter plot of local Moran’s I index for PM_2.5_.

As revealed in Table 4, the distribution of PM_2.5_ concentrations across China shows consistently positive spatial correlation from 2013 to 2023, with global Moran’s I values ranging from 0.399 to 0.594 (all *p* < 0.001, z-scores >3.5), indicating strong clustering where similar levels are geographically proximate due to shared emissions, meteorology, and economic factors. A slight decline in Moran’s I over time (e.g., 0.594 in 2014 to 0.399 in 2022) suggests weakening clusters, likely from air quality policies reducing eastern disparities. Local Moran’s I analysis reveals spatial heterogeneity: as shown in Figure 3, high–high (HH) clusters dominate in eastern industrialized areas (e.g., Beijing–Tianjin–Hebei, Shandong, Henan) from urban emissions, while low–low (LL) clusters prevail in western regions (e.g., Qinghai, Tibet, Yunnan) with sparse populations and better dispersion. Dominant HH/LL patterns reflect positive dependencies, with rare high–low/low–high outliers marking transitional zones influenced by industrial relocations or topography. This aligns with the “eastern high, western low” PM_2.5_ pattern, informing targeted interventions.

**Table 4:** Global Moran’s I index for PM_2.5_ from 2013 to 2023.

Variables	I	E(I)	sd(I)	z	*p*-value*
2013	0.579	−0.03	0.119	5.109	0
2014	0.594	−0.03	0.120	5.224	0
2015	0.592	−0.03	0.120	5.209	0
2016	0.540	−0.03	0.120	4.753	0
2017	0.553	−0.03	0.120	4.846	0
2018	0.519	−0.03	0.121	4.553	0
2019	0.502	−0.03	0.120	4.428	0
2020	0.400	−0.03	0.120	3.581	0
2021	0.446	−0.03	0.121	3.947	0
2022	0.399	−0.03	0.120	3.570	0
2023	0.445	−0.03	0.120	3.945	0

### 3.2 Spatial–temporal distribution characteristics and spatial autocorrelation analysis of O_3_ pollution

**Figure 4:**
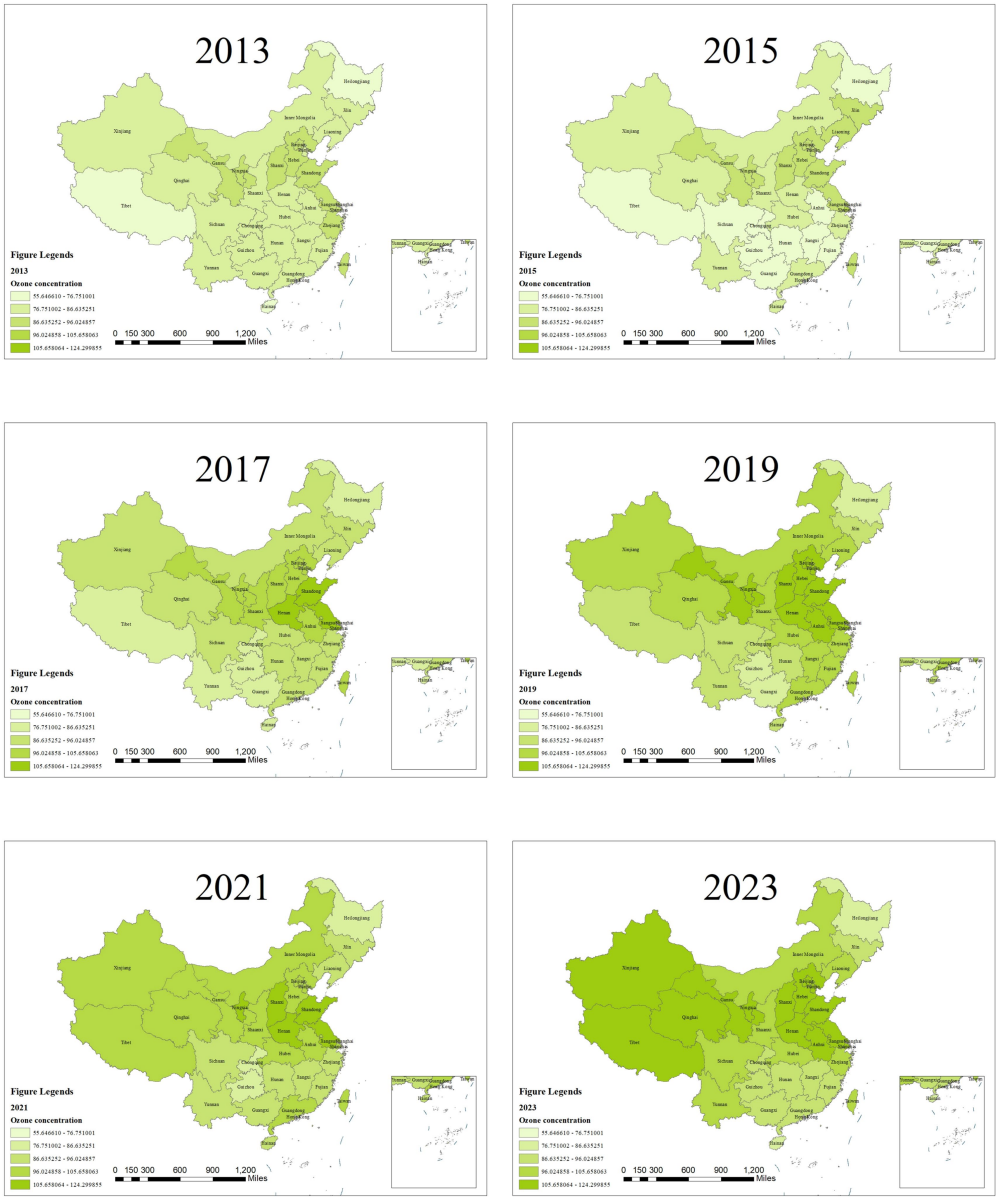
Schematic of O_3_ concentration from 2013 to 2023.

As shown in Figure 4, the distribution of O_3_ concentrations in China also features lower levels in the west and higher levels in the east. Relatively high O_3_ concentrations are observed in Hebei, Shandong, Henan, Hubei and Chongqing in eastern China, as well as in the Jiangsu–Zhejiang region. From 2013 to 2023, the overall O_3_ concentration in China exhibited an upward trend. In 2013, the annual average O_3_ concentrations in all provinces of China basically met the standard, specifically the first level of the national ambient air quality standards, with O_3_ concentrations below 100 μg/m^3^. During 2013–2014, O_3_ concentrations showed an upward trend in Beijing, Hebei Province and Shandong Province in eastern China. However, in regions such as Shanxi Province, Sichuan Province and Anhui Province, O_3_ concentrations presented a downward trend. After 2015, O_3_ concentrations continued to rise.

**Figure 5:**
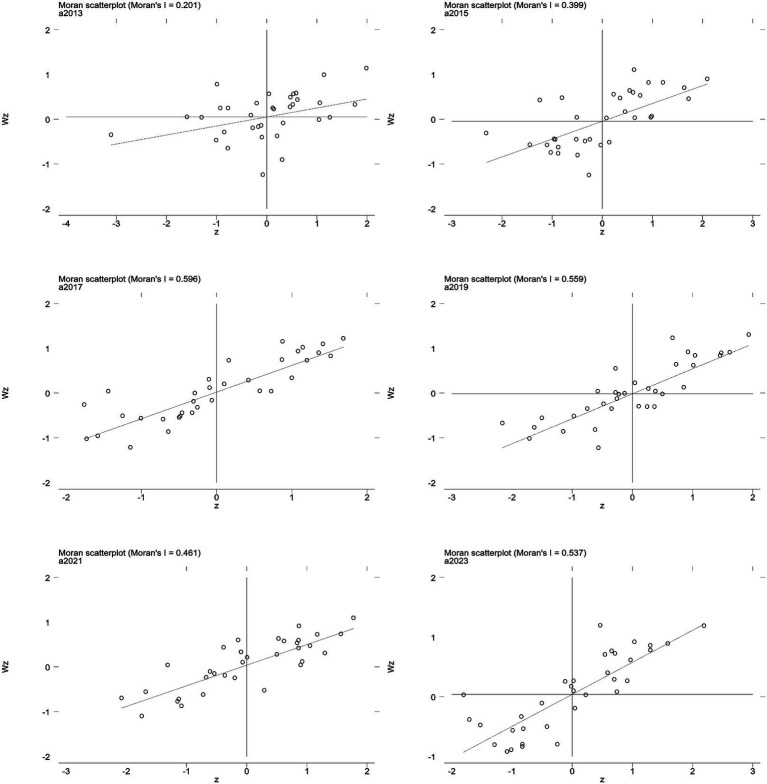
Scatter plot of local Moran’s I index for O_3._

As indicated in Table 5, the distribution of O_3_ concentrations across China exhibits consistently positive spatial correlation from 2013 to 2023, with global Moran’s I values ranging from 0.214 to 0.636 (all *p* < 0.05, z-scores >2.0), signifying strong clustering where similar levels are geographically proximate, influenced by shared precursors (e.g., NOx, VOCs), photochemical processes, and regional meteorology (18, 20). An overall increase in Moran’s I over time (e.g., from 0.214 in 2013 to 0.636 in 2020) reflects intensifying spatial dependencies, likely driven by rising O_3_ trends in eastern urban areas post-2015. Local Moran’s I analysis highlights spatial heterogeneity: as illustrated in Figure 5, high–high (HH) clusters predominate in eastern provinces (e.g., Hebei, Shandong, Henan, Jiangsu) due to high emissions and favorable conditions for O_3_ formation, while low–low (LL) clusters dominate western regions (e.g., Tibet, Qinghai, Xinjiang) with lower precursor loads and dispersion. These HH/LL patterns underscore positive spatial associations, with occasional high–low/low–high outliers indicating transitional areas affected by cross-regional transport or policy variations. This structure mirrors the “eastern high, western low” O_3_ distribution, guiding synergistic control strategies.

**Table 5:** Global Moran’s I index for O_3_ from 2013 to 2023.

Variables	I	E(I)	sd(I)	z	*p*-value*
2013	0.214	−0.030	0.116	2.098	0.018
2014	0.380	−0.030	0.119	3.446	0.000
2015	0.423	−0.030	0.120	3.784	0.000
2016	0.530	−0.030	0.116	4.841	0.000
2017	0.634	−0.030	0.121	5.484	0.000
2018	0.613	−0.030	0.121	5.322	0.000
2019	0.593	−0.030	0.120	5.196	0.000
2020	0.636	−0.030	0.121	5.505	0.000
2021	0.490	−0.030	0.121	4.312	0.000
2022	0.555	−0.030	0.120	4.863	0.000
2023	0.571	−0.030	0.121	4.983	0.000

### 3.3 Changes in the health impacts of PM_2.5_ and O_3_

#### 3.3.1 Changes in the health impacts of PM_2.5_

Based on the commonly used health effect endpoints related to the health impacts of PM_2.5_ and O_3_, as well as the available baseline incidence and mortality data in this research, all-causes mortality, respiratory disease mortality, and cardiovascular disease mortality were selected as the health effect endpoints. This study calculated the total number of national all-causes deaths, cardiovascular disease deaths, and respiratory disease deaths that could be reduced due to excessive PM_2.5_ concentrations when the annual average PM_2.5_ concentrations in each province from 2013 to 2023 were all reduced to 0 μg/m^3^ and the national first-level annual average concentration limit (15 μg/m^3^) (44). The threshold of 0 μg/m^3^ means that any pollution will have health effects (28), and 15 μg/m^3^ is not only a national first-level standard, but also a WHO safety standard (45, 46).

As shown in Table 6, with 0 μg/m^3^ benchmark, the number of all-cause deaths nationwide decreased by 233,174 cases in 2023 compared with 2013, representing a reduction of 47.41%. The number of cardiovascular disease deaths decreased by 56,086 cases, with a reduction of 47.71%, while the number of respiratory disease deaths decreased by 38,519 cases, corresponding to a 46.97% reduction. However, with 15 μg/m^3^ as the benchmark, the number of all-cause deaths nationwide decreased by 240,448 cases in 2023 compared with 2013, a reduction of 65.55%. The number of cardiovascular disease deaths decreased by 57,504 cases, a reduction of 65.75%, and the number of respiratory disease deaths decreased by 40,055 cases, corresponding to a 65.27% reduction.

**Table 6:** Cases of different health effect endpoints caused by PM_2.5_ pollution from 2013 to 2023 with 0 μg/m^3^ and 15 μg/m^3^ benchmark (unit: 10000 cases).

PM_2.5_	0 μg/m^3^			15 μg/m^3^		
	All-cause mortality	Respiratory mortality	Cardiovascular mortality	All-cause mortality	Respiratory mortality	Cardiovascular mortality
2013	49.1804	8.2	11.7549	36.6789	6.1369	8.7458
2014	47.1212	7.864	12.2555	34.503	5.7784	8.2215
2015	40.3931	6.7559	9.6339	27.6183	4.6351	6.5714
2016	37.5177	6.2816	8.9415	24.6166	4.1357	5.8529
2017	35.9181	6.0181	8.556	22.9206	3.8538	5.4467
2018	32.3062	5.4195	7.6891	19.2085	3.2335	4.5609
2019	30.6138	5.138	7.284	17.4454	2.9377	4.1413
2020	27.5338	4.6257	6.5467	14.3038	2.4109	3.3933
2021	26.1598	4.3979	6.217	12.9066	2.1774	3.06
2022	24.6778	4.1502	5.8634	11.4127	1.9257	2.7055
2023	25.863	4.3482	6.1463	12.6341	2.1314	2.9954
2013–2023 difference	23.3174	3.8519	5.6086	24.0448	4.0055	5.7504

**Figure 6:**
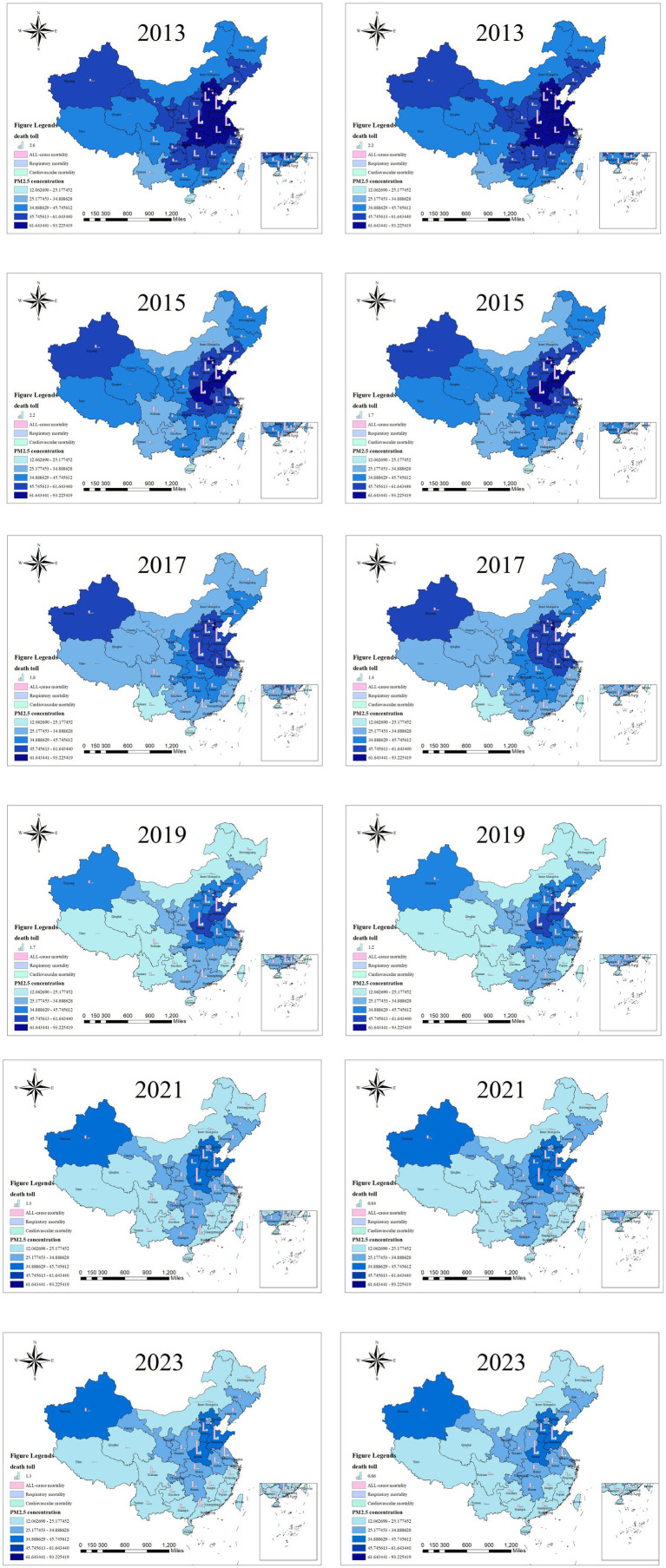
Different health effect endpoints caused by PM_2.5_ pollution from 2013 to 2023 with 0 μg/m^3^ and 15 μg/m^3^ benchmark (unit: 10000 cases). The left column presents different health effect endpoints caused by PM_2.5_ pollution from 2013 to 2023 under the 0 μg/m^3^ benchmark, the right column presents different health effect endpoints caused by PM_2.5_ pollution from 2013 to 2023 under the 15 μg/m^3^ benchmark.

As can be seen from Figure 6, no matter from the 0 μg/m^3^ or 15 μg/m^3^ benchmark perspective, the number of provincially avoidable deaths attributed to PM_2.5_ in China showed an overall downward trend from 2013 to 2023, maintaining the characteristic of being lower in the western regions and higher in the eastern regions. After 2019, the number of provincially avoidable deaths in Xinjiang increased, but it continued to decline after 2019. This phenomenon is linked to the relocation of high-pollution enterprises to western regions driven by policy factors (47). Provinces with relatively high PM_2.5_ concentrations, such as Henan and Shandong, also had a higher number of premature deaths caused by PM_2.5_. However, in provincial-level administrative units with high PM_2.5_ concentrations like Beijing and Tianjin, the number of premature deaths attributed to PM_2.5_ was relatively low due to their smaller total population. Sensitivity with GEMM (26) indicated higher burdens for older populations, addressing age vulnerability and demographic amplification.

#### 3.3.2 Changes in the health impacts of O_3_

For O_3_, 0 μg/m^3^ was chosen under the same no-threshold assumption, while 60 μg/m^3^ corresponds to the World Health Organization’s guideline for peak-season average O_3_ concentration to reflect international health protection benchmarks (48, 49). As shown in Table 7, with 0 μg/m^3^ as the benchmark, the number of all-cause deaths nationwide increased by 365,084 cases in 2023 compared with 2013, representing a rise of 24.25%. The number of cardiovascular disease deaths increased by 80,056 cases, with an increase of 25.43%, while the number of respiratory disease deaths increased by 98,620 cases, corresponding to a 15.46% rise. With 60 μg/m^3^ as the benchmark, the number of all-cause deaths nationwide increased by 372,724 cases in 2023 compared with 2013, a growth of 79.70%. The number of cardiovascular disease deaths increased by 77,497 cases, with a growth of 81.52%, and the number of respiratory disease deaths increased by 163,165 cases, showing an increase of 64.54%.

**Table 7:** Cases of different health effect endpoints caused by O_3_ pollution from 2013 to 2023 with 0 μg/m^3^ and 60 μg/m^3^ benchmark (unit: 10000 cases).

O_3_	0 μg/m^3^			60 μg/m^3^		
	All-cause mortality	Respiratory mortality	Cardiovascular mortality	All-cause mortality	Respiratory mortality	Cardiovascular mortality
2013	150.5394	63.8014	31.4851	46.7631	25.2829	9.5066
2014	152.2225	64.3584	31.848	47.8301	25.7031	9.7305
2015	149.4248	63.6798	31.2322	44.0679	23.8621	8.9577
2016	158.0657	66.1772	33.1115	52.9839	28.1236	10.7938
2017	171.832	69.8409	36.1308	67.7888	34.783	13.8628
2018	174.9081	70.7129	36.8025	70.7754	36.1127	14.4828
2019	183.5999	72.8676	38.7239	80.1185	40.0005	16.4357
2020	178.4347	71.6817	37.5758	74.1151	37.5069	15.1807
2021	178.611	71.7961	37.6082	74.2709	37.6975	15.2067
2022	186.2596	73.5283	39.3118	82.9607	41.1793	17.03
2023	187.0478	73.6634	39.4906	84.0355	41.5994	17.2563
2013–2023 difference	36.5084	9.862	8.0056	37.2724	16.3165	7.7497

**Figure 7:**
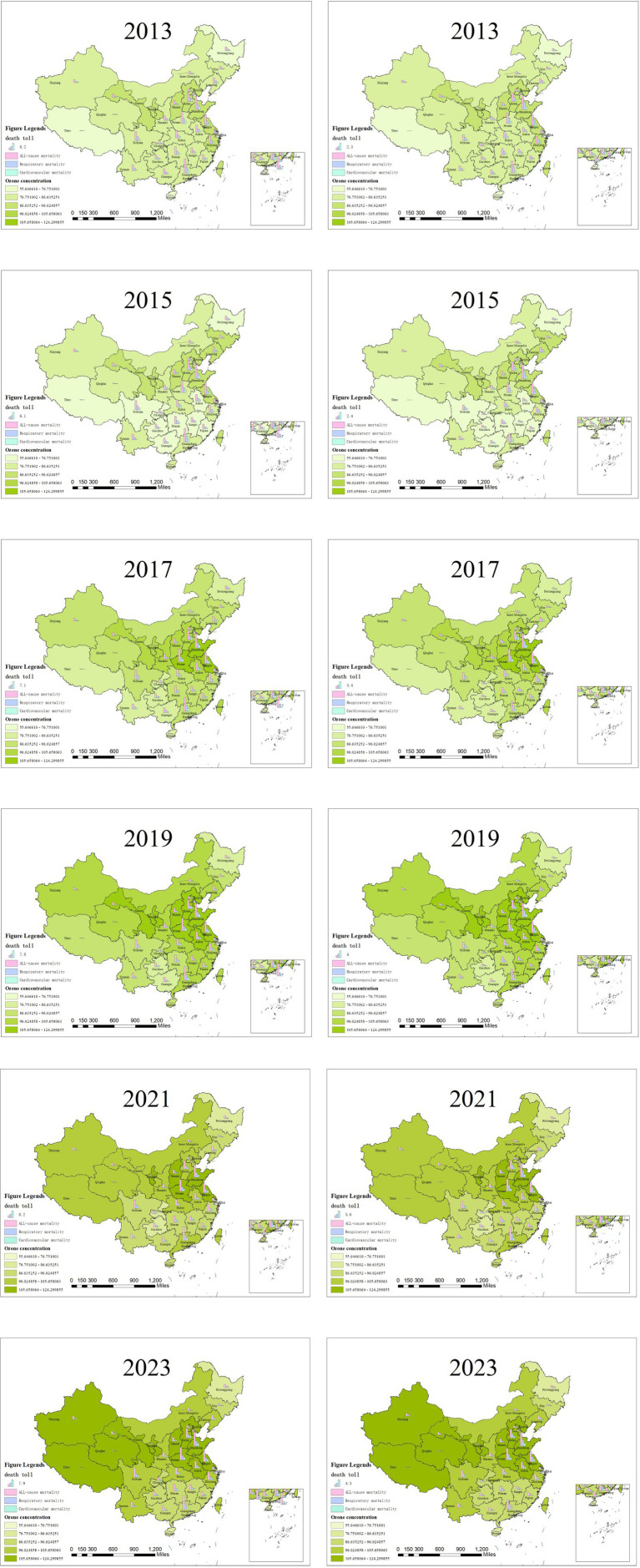
Different health effect endpoints caused by O_3_ pollution from 2013 to 2023 with 0 μg/m^3^ and 60 μg/m^3^ benchmark (unit: 10000 cases). The left column presents different health effect endpoints caused by O_3_ pollution from 2013 to 2023 under the 0 μg/m^3^ benchmark, the right column presents different health effect endpoints caused by O_3_ pollution from 2013 to 2023 under the 60 μg/m^3^ benchmark.

As can be seen from Figure 7, the number of premature deaths caused by O_3_ in China has shown an overall upward trend, with a general pattern of lower in the western and higher in eastern regions. In 2013, Guangdong, Shandong, and Henan Province recorded the highest number of premature deaths attributed to O_3_. Among these, Henan and Shandong Provinces also had a relatively high number of premature deaths caused by PM_2.5_ in 2013.

From 2013 to 2015, the total number of premature deaths caused by O_3_ in China showed a decreasing trend, with the most significant declines observed in Guangdong and Jiangxi Province. Based on a benchmark of 0 μg/m^3^, the number of premature deaths decreased by 9,851 in Guangdong Province and by 4,935 in Jiangxi Province. When using 60 μg/m^3^ as the benchmark, the respective decreases were 12,793 in Guangdong Province and 5,603 in Jiangxi Province. From 2015 to 2021, the total number of premature deaths caused by O_3_ nationwide showed an overall upward trend. With 0 μg/m^3^ benchmark, the increase in the number of such deaths reached 255,342; while based on 60 μg/m^3^ benchmark, the increase stood at 271,345. Among all provinces, Guangdong, Shandong, and Henan witnessed the largest growth in premature deaths attributed to O_3_. From 2021 to 2023, the number of premature deaths caused by O_3_ remained relatively stable at first and then showed an upward trend. This phenomenon is related to the outbreak of the COVID-19 pandemic, which exerted a significant impact on people’s production and lifestyle. Consequently, it altered the concentration of O_3_ in the atmosphere and further affected the number of premature deaths caused by O_3_.

### 3.4 Health economic losses caused by PM_2.5_ and O_3_ pollution

#### 3.4.1 National trends in health economic losses caused by PM_2.5_ pollution

The nominal values of health economic costs in 2013 and 2023 are relatively close, which may be due to the fact that a direct comparison of nominal values can mask changes in actual health economic costs and real GDP. Therefore, in this study, the nominal values of health economic costs are excluded when calculating the GDP share of health economic costs.

As shown is Tables 8, 9, in the assessment of health economic losses caused by PM_2.5_ under the 0 μg/m^3^ benchmark, the nominal values of health economic costs remained relatively stable, while their GDP share showed a significant downward trend. From 2013 to 2023, the economic costs resulting from health impacts caused by PM_2.5_ pollution decreased by 5.389 billion yuan when calculated using the AHC method, and by 9.471 billion yuan using the WTP method; the GDP share in both cases decreased by 0.23%. In the assessment of health economic losses caused by PM_2.5_ under the 15 μg/m^3^ benchmark, both the nominal values of health economic costs and their GDP share exhibited a significant downward trend. From 2013 to 2023, the economic costs arising from health impacts due to PM_2.5_ pollution dropped by 36.591 billion yuan as per the AHC, and by 64.312 billion yuan according to the WTP, with the GDP share decreasing by 0.12% in both scenarios. The above results indicate that the health economic costs associated with PM_2.5_ in China actually showed a downward trend from 2013 to 2023.

**Table 8:** Health economic losses caused by PM_2.5_ pollution in China from 2013 to 2023 with 0 μg/m^3^ and 15 μg/m^3^ benchmark (100 million yuan).

PM_2.5_	0 μg/m^3^						15 μg/m^3^					
	All-cause mortality		Respiratory mortality		Cardiovascular mortality		All-cause mortality		Respiratory mortality		Cardiovascular mortality	
	AHC	WTP	AHC	WTP	AHC	WTP	AHC	WTP	AHC	WTP	AHC	WTP
2013	1298.72	2282.59	216.54	380.58	310.42	545.58	969.79	1704.48	162.26	285.19	231.24	406.42
2014	1345.15	2364.19	224.48	394.54	321.31	564.73	986.51	1733.86	165.21	290.37	235.07	413.16
2015	1239.53	2178.57	207.3	364.34	295.65	519.62	850.53	1494.87	142.73	250.87	202.38	355.7
2016	1222.54	2148.7	204.69	359.76	291.36	512.09	802.72	1410.84	134.86	237.03	190.86	335.44
2017	1254.61	2205.08	210.21	369.47	298.86	525.26	801.47	1408.64	134.76	236.85	190.45	334.74
2018	1203.83	2115.82	201.96	354.95	286.51	503.57	715.39	1257.35	120.43	211.67	169.86	298.54
2019	1209.98	2126.63	203.07	356.92	287.89	505.99	691.65	1215.62	116.47	204.71	164.18	288.56
2020	1135.51	1995.75	190.78	335.31	269.97	474.5	586.6	1,031	98.88	173.79	139.15	244.57
2021	1154.48	2029.08	194.1	341.15	274.35	482.2	564.58	992.3	95.25	167.42	133.85	235.25
2022	1127.75	1982.1	189.68	333.38	267.93	470.9	513.96	903.33	86.73	152.44	121.83	214.12
2023	1244.83	2187.88	209.3	367.86	295.82	519.92	603.88	1061.36	101.88	179.07	143.16	251.62

**Table 9:** GDP share of national PM_2.5_ health economic costs based on 0 μg/m^3^ and 15 μg/m^3^ benchmark from 2013 to 2023.

PM_2.5_	0 μg/m^3^		15 μg/m^3^	
	AHC proportion	WTP proportion	AHC proportion	WTP proportion
	%	%	%	%
2013	0.23	0.4	0.17	0.3
2014	0.21	0.37	0.16	0.27
2015	0.18	0.32	0.13	0.22
2016	0.16	0.29	0.11	0.19
2017	0.15	0.27	0.1	0.17
2018	0.13	0.24	0.08	0.14
2019	0.12	0.21	0.07	0.12
2020	0.11	0.2	0.06	0.1
2021	0.1	0.18	0.05	0.09
2022	0.09	0.16	0.04	0.07
2023	0.1	0.17	0.05	0.08

#### 3.4.2 Inter-provincial differences in PM_2.5_ health economic losses

**Figure 8:**
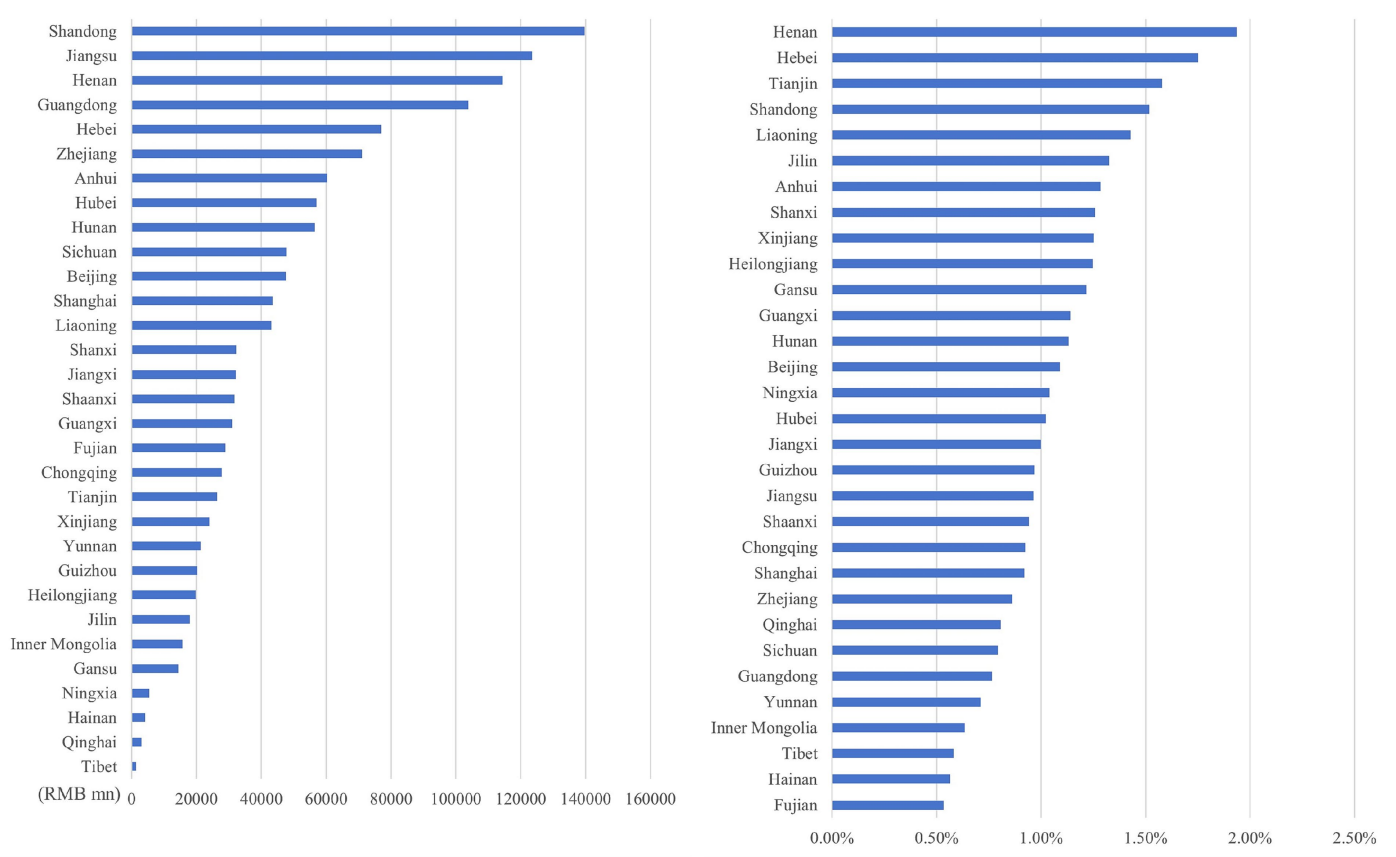
Ranking of PM_2.5_ health economic costs and their GDP shares based on the 0 μg/m^3^ benchmark using the AHC method.

**Figure 9:**
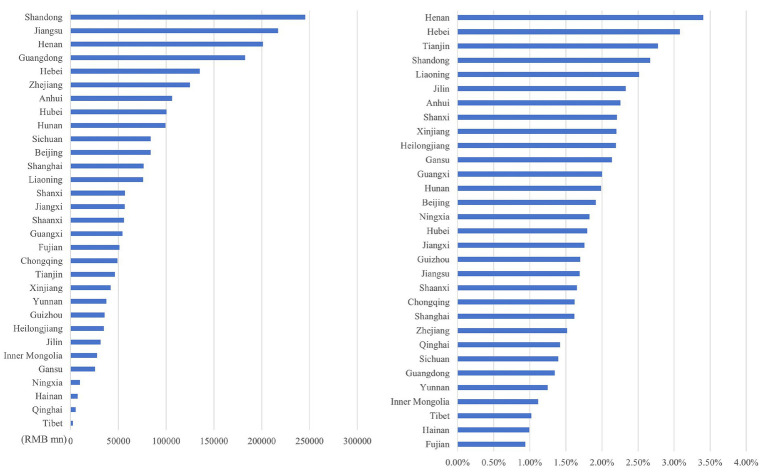
Ranking of PM_2.5_ health economic costs and their GDP shares based on the 0 μg/m^3^ benchmark using the WTP method.

**Figure 10:**
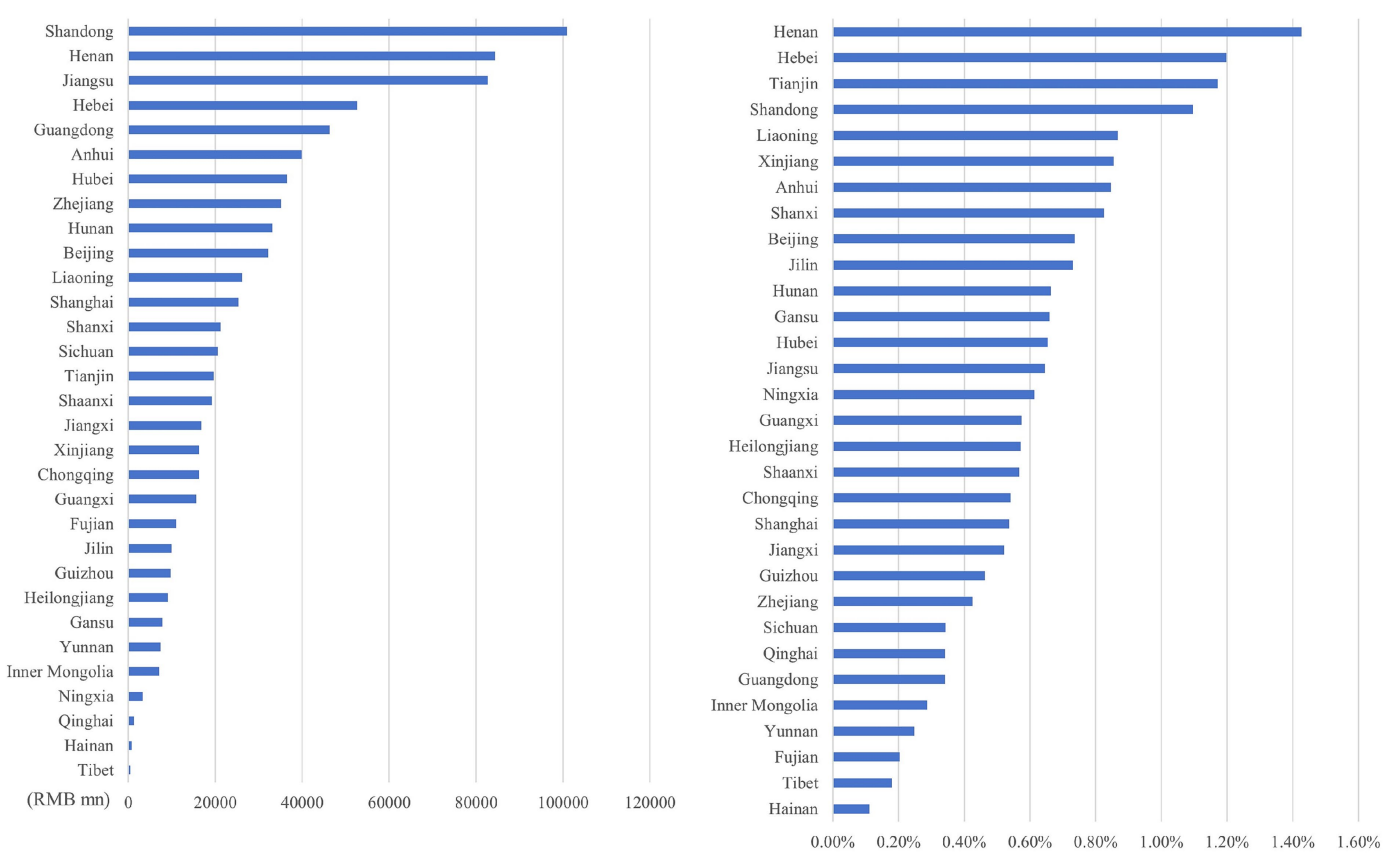
Ranking of PM_2.5_ health economic costs and their GDP shares based on the 15 μg/m^3^ benchmark using the AHC method.

**Figure 11:**
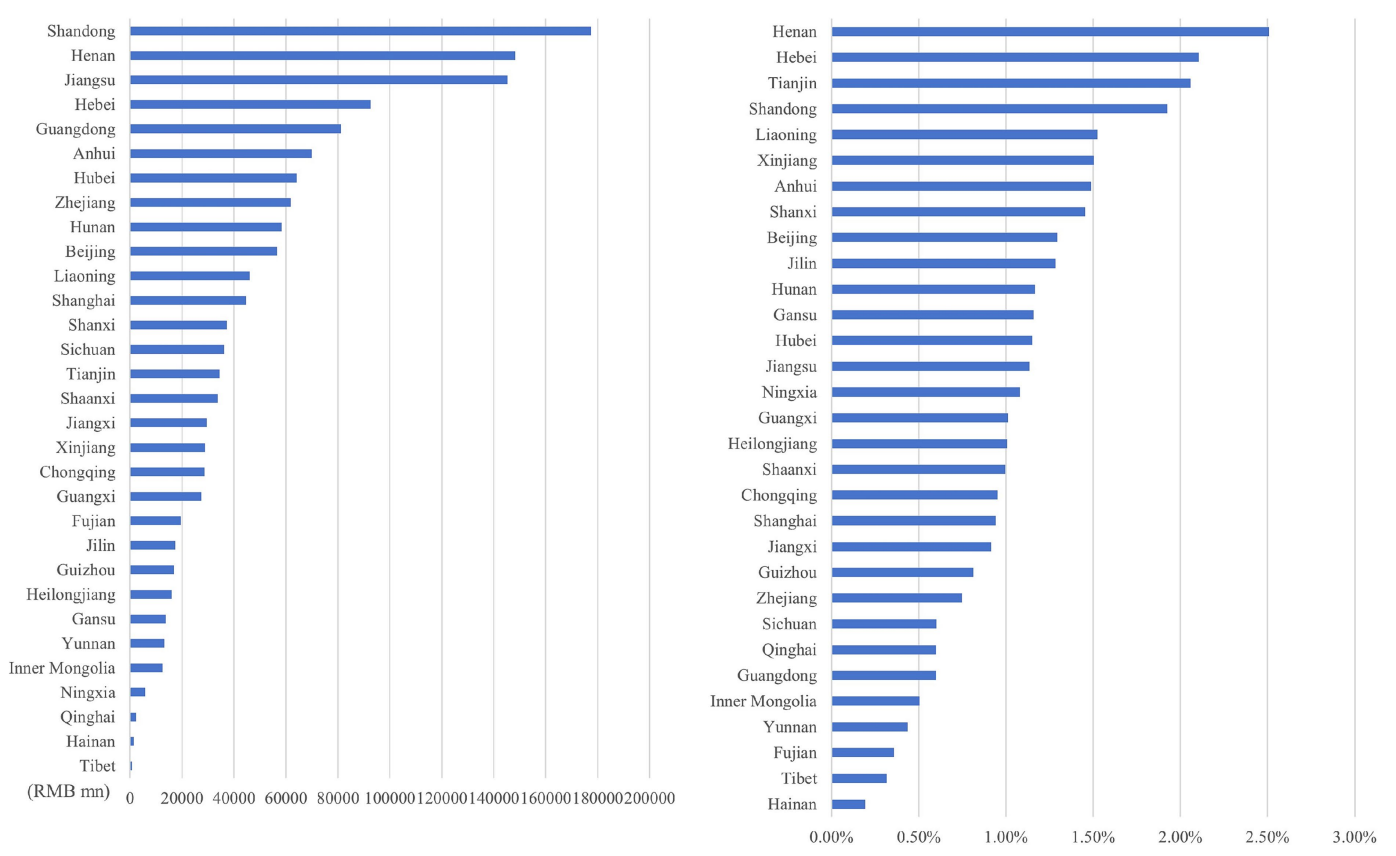
Ranking of PM_2.5_ health economic costs and their GDP shares based on the 15 μg/m^3^ benchmark using the WTP method.

Through the ranking the health economic costs caused by PM_2.5_ pollution and the proportion of their total amount in the local GDP of 2023 across provinces in China (excluding Hong Kong, Macao, and Taiwan) from 2013 to 2023, the results presented from Figures 8–11 are obtained.

In terms of spatial distribution, both the health economic costs of PM_2.5_ and their GDP shares show a pattern of being higher in eastern China and lower in western China. Under both 0 μg/m^3^ and 15 μg/m^3^ benchmarks, Shandong, Henan, and Jiangsu Provinces rank top three in terms of health economic costs caused by PM_2.5_, and these three provinces also have relatively high PM_2.5_ concentrations in the country. By comparing the ratio of PM_2.5_ related health economic costs to GDP across provinces, the health economic losses incurred during the economic development process of each province can be analyzed. Meanwhile, from the rankings of health economic costs and their GDP shares in provinces such as the three northeastern provinces, Shanxi, and Xinjiang, it can be observed that there are differences between the ranking of PM_2.5_ pollution-related health economic costs and that of their GDP shares. This indicates that although the PM_2.5_ concentrations in these regions are relatively low, the health losses caused per unit of economic GDP are greater.

#### 3.4.3 National trends in health economic losses caused by O_3_ pollution

As can be seen from Tables 10, 11, both the nominal value of health economic costs caused by O_3_ and its proportion in GDP show a significant upward trend. Taking 0 μg/m^3^ benchmark as an example, the health economic costs resulting from health impacts caused by O_3_ pollution increased from 2013 to 2023 under the AHC method. However, under the WTP method the proportion in GDP rising by only 0.1%. With 15 μg/m^3^ benchmark, the health economic costs from O_3_ pollution induced health impacts also saw an increase between 2013 and 2023: the AHC method showed a rise of 283.531 billion yuan, while the WTP method indicated an increase of 498.327 billion yuan, accompanied by a 0.18% growth in the GDP proportion. These figures indicate that China’s health economic costs associated with O_3_ actually presented an upward trend from 2013 to 2023.

**Table 10:** Health economic losses caused by O_3_ pollution in China from 2013 to 2023 with 0 μg/m^3^ and 60 μg/m^3^ benchmark (100 million yuan).

O_3_	0 μg/m^3^						60 μg/m^3^					
	All-cause mortality		Respiratory mortality		Cardiovascular mortality		All-cause mortality		Respiratory mortality		Cardiovascular mortality	
	AHC	WTP	AHC	WTP	AHC	WTP	AHC	WTP	AHC	WTP	AHC	WTP
2013	3988.37	7009.87	1686.32	2963.83	834.41	1466.54	1260.79	2215.93	679.8	1194.81	256.39	450.62
2014	4378.75	7695.99	1843.5	3240.1	916.6	1,611	1417.55	2491.44	758.15	1332.51	288.53	507.12
2015	4590.41	8067.99	1950.31	3427.82	959.84	1686.99	1386.13	2436.23	747.8	1314.32	281.87	495.42
2016	5168.84	9084.62	2160.35	3796.98	1082.99	1903.44	1752.32	3079.84	928.57	1632.02	357.05	627.54
2017	6032.12	10601.91	2445.58	4298.28	1268.76	2229.94	2409.69	4235.22	1233.44	2167.87	492.91	866.33
2018	6546.33	11505.68	2643.16	4645.55	1377.63	2421.3	2666.11	4685.89	1358.9	2388.38	545.63	958.99
2019	7264.22	12767.41	2877.54	5057.49	1532.49	2693.47	3195.05	5615.55	1592.38	2798.73	655.57	1152.21
2020	7445.38	13085.82	2984.46	5245.41	1568.32	2756.44	3123.39	5489.59	1577.4	2772.4	639.89	1124.66
2021	7973.96	14014.83	3201.48	5626.84	1679.23	2951.38	3334.08	5859.89	1690.48	2971.15	682.72	1199.93
2022	8655.06	15211.92	3410.62	5994.42	1827.14	3211.34	3881.74	6822.46	1923.46	3380.62	796.99	1400.77
2023	9083.46	15964.86	3573.75	6281.13	1917.99	3371.01	4096.1	7199.2	2025.65	3560.23	841.21	1478.49

**Table 11:** GDP share of national O_3_ health economic costs based on 0 μg/m^3^ and 60 μg/m^3^ benchmark from 2013 to 2023.

O_3_	0 μg/m^3^		60 μg/m^3^	
	AHC proportion	WTP proportion	AHC proportion	WTP proportion
	%	%	%	%
2013	0.70	1.23	0.22	0.39
2014	0.69	1.21	0.22	0.39
2015	0.68	1.19	0.2	0.36
2016	0.69	1.22	0.24	0.41
2017	0.73	1.28	0.29	0.51
2018	0.73	1.28	0.3	0.52
2019	0.73	1.29	0.32	0.57
2020	0.73	1.29	0.31	0.54
2021	0.70	1.23	0.29	0.51
2022	0.72	1.26	0.32	0.56
2023	0.72	1.27	0.32	0.57

#### 3.4.4 Inter-provincial differences in O_3_ health economic losses

**Figure 12:**
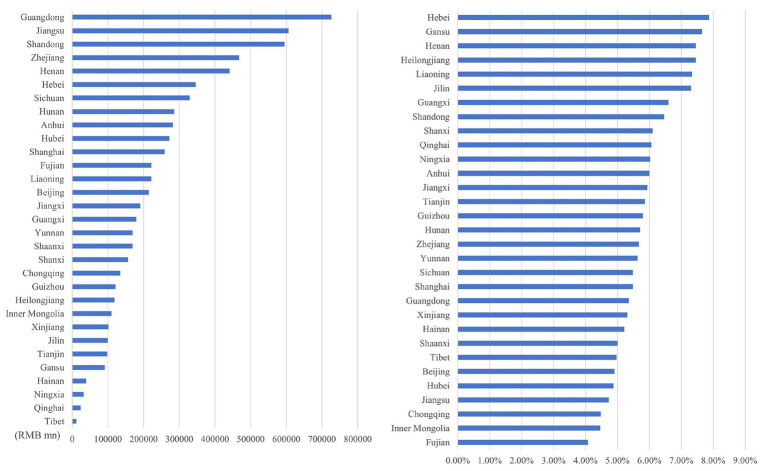
Ranking of O_3_ health economic costs and their GDP shares based on the 0 μg/m^3^ benchmark using the AHC method.

**Figure 13:**
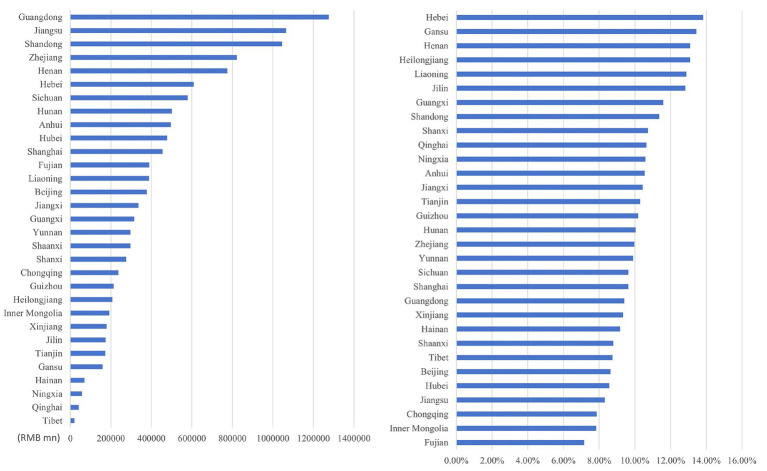
Ranking of O_3_ health economic costs and their GDP shares based on the 0 μg/m^3^ benchmark using the WTP method.

**Figure 14:**
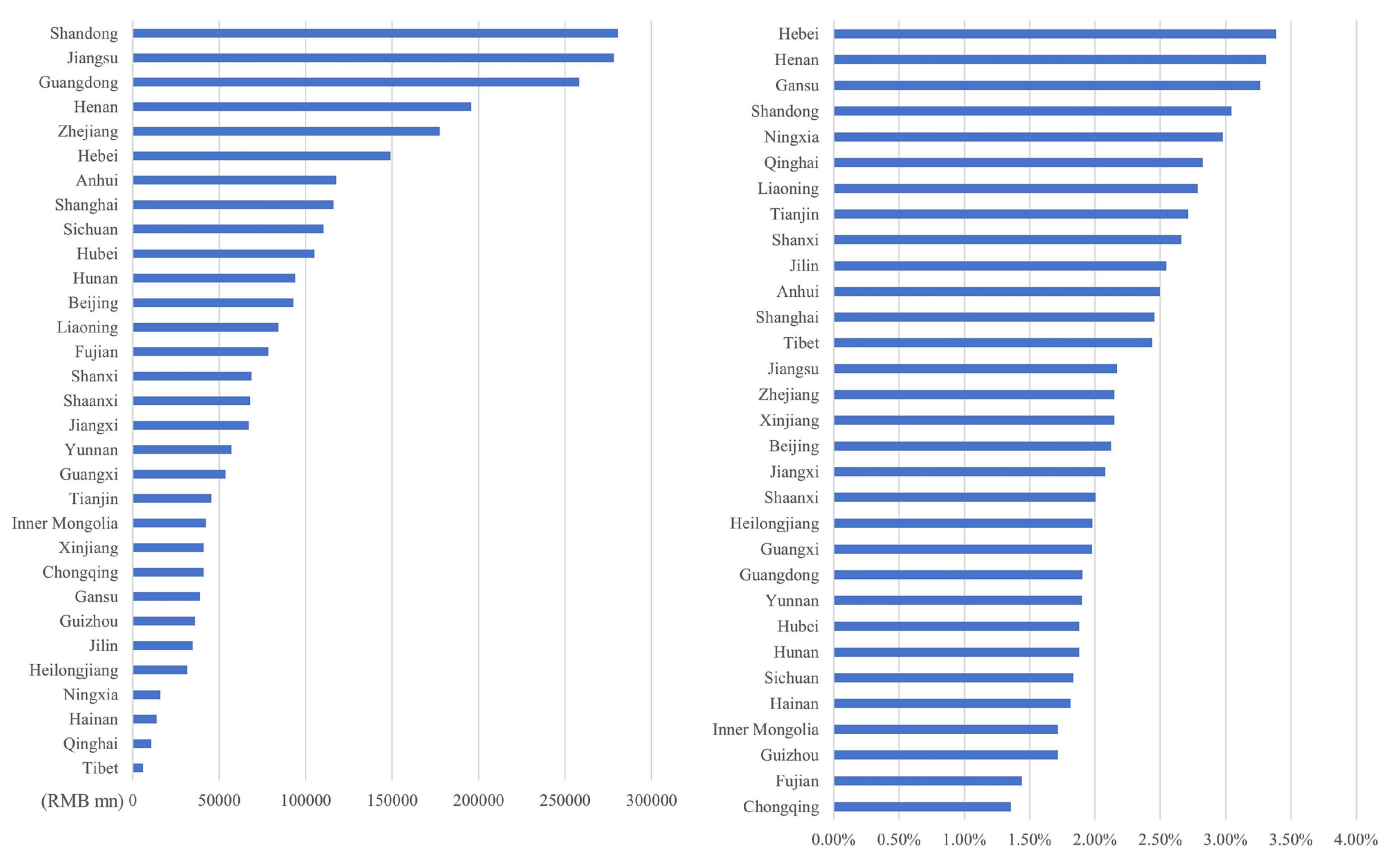
Ranking of O_3_ health economic costs and their GDP shares based on the 60 μg/m^3^ benchmark using the AHC method.

**Figure 15:**
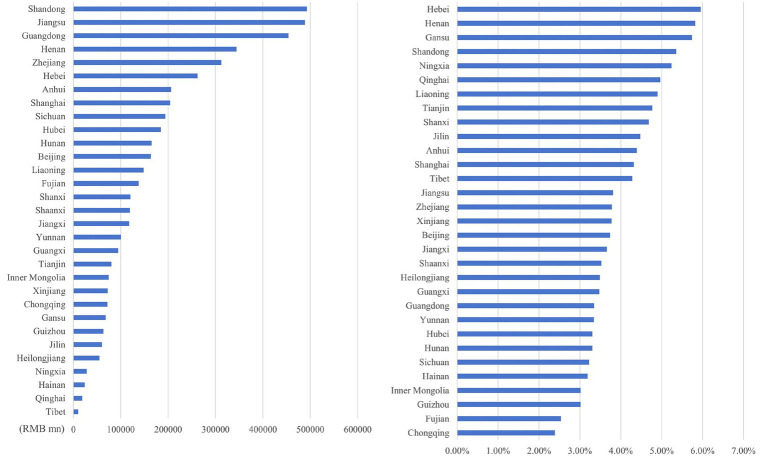
Ranking of O_3_ health economic costs and their GDP shares based on the 60 μg/m^3^ benchmark using the WTP method.

As illustrated in Figures 12–15, in terms of spatial distribution, both the health economic costs caused by O_3_ and their proportion in GDP show a pattern of being higher in the eastern regions and lower in the western regions. Meanwhile, there is a discrepancy between the rankings of health economic costs and those of health economic costs as a percentage of GDP.

Under both 0 μg/m^3^ and 60 μg/m^3^ benchmarks, the top three provinces with the highest health economic costs caused by O_3_ are Guangdong, Jiangsu, and Shandong provinces, which corresponds to the provinces with relatively high O_3_ concentrations. However, under both benchmarks, the top three provinces in terms of the proportion of O_3_ induced health economic costs in GDP are Hebei, Henan, and Gansu provinces. Among them, Hebei and Henan provinces have a relatively large number of high-energy-consumption and high-pollution enterprises. As one of the provinces supported by the national western development strategy, Gansu is also affected by this factor in terms of its O_3_-related health economic costs and their proportion in GDP. The higher the health loss per unit of GDP, the more significant the burden that health economic costs impose on the overall economic situation.

## 4 Discussion

This study quantifies the health economic burdens associated with PM_2.5_ and O_3_ pollution in mainland China (excluding Hong Kong, Macao, and Taiwan) from 2013 to 2023, revealing divergent temporal trends. Nationally, PM_2.5_-related health economic burdens exhibited a consistent downward trajectory, while O_3_-related costs increased substantially. These trends align with findings from previous research (50–52), despite discrepancies in absolute cost estimates. Such variations may arise from differences in data sources and methodological choices, particularly threshold settings for exposure-response functions.

In contrast to most existing studies, which typically employ a single threshold, this analysis incorporated multiple scenarios to account for emerging epidemiological evidence indicating health risks at low pollutant concentrations (53, 54). For PM_2.5_, costs were estimated under a “0 μg/m^3^ threshold” (assuming harm at any exposure level) and a “15 μg/m^3^ threshold” (aligned with China’s Grade I standard). For O_3_, scenarios included a “0 μg/m^3^ threshold” and a “60 μg/m^3^ threshold” (based on the World Health Organization’s guideline). This difference might result in significantly higher health economic costs under the “0 μg/ m^3^ and 60 μg/m^3^ threshold” scenarios compared to traditional studies. Comparative results indicated that the comparability of health economic cost studies was highly dependent on threshold selection, and policy formulation should integrate local standards with the latest scientific evidence (55).

The observed trends reaffirm the effectiveness of China’s air pollution control measures, such as the Air Pollution Prevention and Control Action Plan and the Three-Year Action Plan for Winning the Blue Sky Defense Battle (2018–2020), which targeted industrial emissions, coal consumption, and vehicle standards (16, 17). These initiatives successfully reduced primary PM_2.5_ precursors (e.g., SO_2_ and NOx), resulting in nationwide declines in PM_2.5_ concentrations and associated mortality, particularly in eastern hotspots like the Beijing–Tianjin–Hebei (BTH) and Yangtze River Delta (YRD) regions. However, the concurrent rise in O_3_ levels reflects complex atmospheric chemistry interactions. Reduced PM_2.5_ aerosols weaken the scavenging of hydroperoxyl radicals (HO₂), thereby promoting O_3_ formation via photochemical reactions involving shared precursors like NOx and volatile organic compounds (VOCs) (18, 20). Climatic factors, including elevated temperatures and solar radiation in southern provinces such as Guangdong, further exacerbate O_3_ production (56).

Spatial autocorrelation analyses identified regionally coherent patterns, characterized by an “eastern high, western low” distribution influenced by industrialization, urbanization, and meteorological conditions. High–High clusters in provinces like Shandong, Henan, and Jiangsu indicate persistent pollution hotspots driven by dense populations and high emissions, whereas Low–Low clusters in western regions reflect lower economic activity and favorable atmospheric dispersion (41). These spatial disparities amplify demographic vulnerabilities, including aging populations in eastern areas that heighten risks of cardiovascular and respiratory diseases (28, 36). Moreover, higher GDP ratios in provinces such as Henan and Hebei underscore socioeconomic inequalities, where pollution burdens disproportionately impact less affluent regions with limited healthcare resources.

These findings provide evidence-based insights for targeted interventions, emphasizing the need for coordinated PM_2.5_ and O_3_ management to mitigate health economic burdens and promote equitable air quality improvements. In the context of China’s carbon peak and neutrality goals, projections of emission scenarios—particularly those estimating population health burdens and economic costs—can offer practical guidance (57). Without enhanced controls, China could face substantial losses, including $4.2 billion in economic impacts and $285 billion in life-value losses by 2030 (30). The study highlights the imperative for synergistic governance, as declining PM_2.5_ levels may inadvertently exacerbate O_3_ pollution if unmanaged. Regional collaboration is essential, given overlapping polluted areas for both pollutants, though O_3_ affects broader regions. This warrants expanding national priority zones to include border areas like Jiangsu–Anhui–Shandong–Henan, establishing state-guided regions, and implementing inter-provincial joint prevention mechanisms.

Globally, O_3_ health risks are often underestimated (58), consistent with this study’s observation that O_3_ has increasingly surpassed PM_2.5_ as a dominant health hazard (59). As air pollution dynamics evolve, revising ambient air quality standards is crucial to balance environmental protection with economic development (60). The feasibility and cost-effectiveness of control measures must be evaluated to minimize adverse impacts on industry and employment (60). Macro-level analyses comparing multiple pollutants across regions and time periods, as conducted here, furnish a scientific foundation for informed policy-making. Sensitivity analyses further indicate that variations in pollutant concentrations primarily drive mortality estimates, rather than baseline mortality rates or population changes (59). Future research should explore refined exposure models and long-term projections to address these challenges. Notably, factors such as improvements in healthcare, strengthening of public health policies, and lifestyle changes (e.g., smoking rates) have also exerted impacts on mortality and morbidity. These could be further incorporated into future studies to enable a more thorough discussion.

## 5 Conclusion

Air pollution is a more important population health risk factor than previously thought (26). This study provides a comprehensive spatiotemporal analysis of PM_2.5_ and O_3_ pollution in China from 2013 to 2023, quantifying their associated health economic costs and trends across 31 provinces. Our findings reveal divergent trajectories in the two pollutants: PM_2.5_ concentrations exhibited a substantial nationwide decline, accompanied by significant reductions in attributable mortality, while O_3_ levels showed a persistent upward trend, leading to increased health burdens. Both pollutants displayed pronounced spatial agglomeration, with higher concentrations and impacts predominantly in eastern regions.

Specifically, PM_2.5_-related all-cause mortality decreased by 47.41% (233,173 fewer cases) and 65.55% (240,448 fewer cases) relative to reference concentrations of 0 μg/m^3^ and 15 μg/m^3^, respectively. Cardiovascular disease mortality declined by 47.71% (56,086 cases) and 65.75% (57,504 cases), and respiratory disease mortality by 46.97% (38,519 cases) and 65.27% (40,055 cases). In contrast, O_3_-attributable all-cause mortality rose by 24.25% (365,084 additional cases) and 79.70% (372,724 additional cases) at 0 μg/m^3^ and 60 μg/m^3^ benchmarks, with cardiovascular mortality increasing by 25.43% (80,056 cases) and 81.52% (77,497 cases), and respiratory mortality by 15.46% (98,620 cases) and 64.54% (163,165 cases).

Spatially, health economic costs and their GDP proportions for both pollutants followed an “eastern high, western low” pattern. For PM_2.5_, Shandong, Henan, and Jiangsu provinces incurred the highest absolute costs, while Henan, Hebei, and Tianjin showed the largest GDP shares. For O_3_, Guangdong, Jiangsu, and Shandong ranked highest in absolute costs, with Henan, Hebei, and Gansu exhibiting the greatest GDP proportions.

These results underscore the success of China’s PM_2.5_ control policies while highlighting the emerging challenge of O_3_ pollution, driven by synergistic precursor interactions and regional disparities. Policymakers should prioritize integrated strategies for co-controlling PM_2.5_ and O_3_, particularly in eastern high-burden areas, to mitigate escalating health and economic impacts. Future research could incorporate finer-scale data and climate change projections to enhance predictive accuracy and inform adaptive measures.

## Data Availability

Publicly available datasets were analyzed in this study. This data can be found at: https://data.tpdc.ac.cn/home.
