# Supplementary figures and images for: Divergent trends and regional disparities in PM2.5 and O3 health economic burdens in China, 2013–2023: an integrated assessment with policy implications
Source: Front Public Health. 2025 Nov 13;13:1683415. doi: 10.3389/fpubh.2025.1683415 (PMC12657416; doi:10.3389/fpubh.2025.1683415)

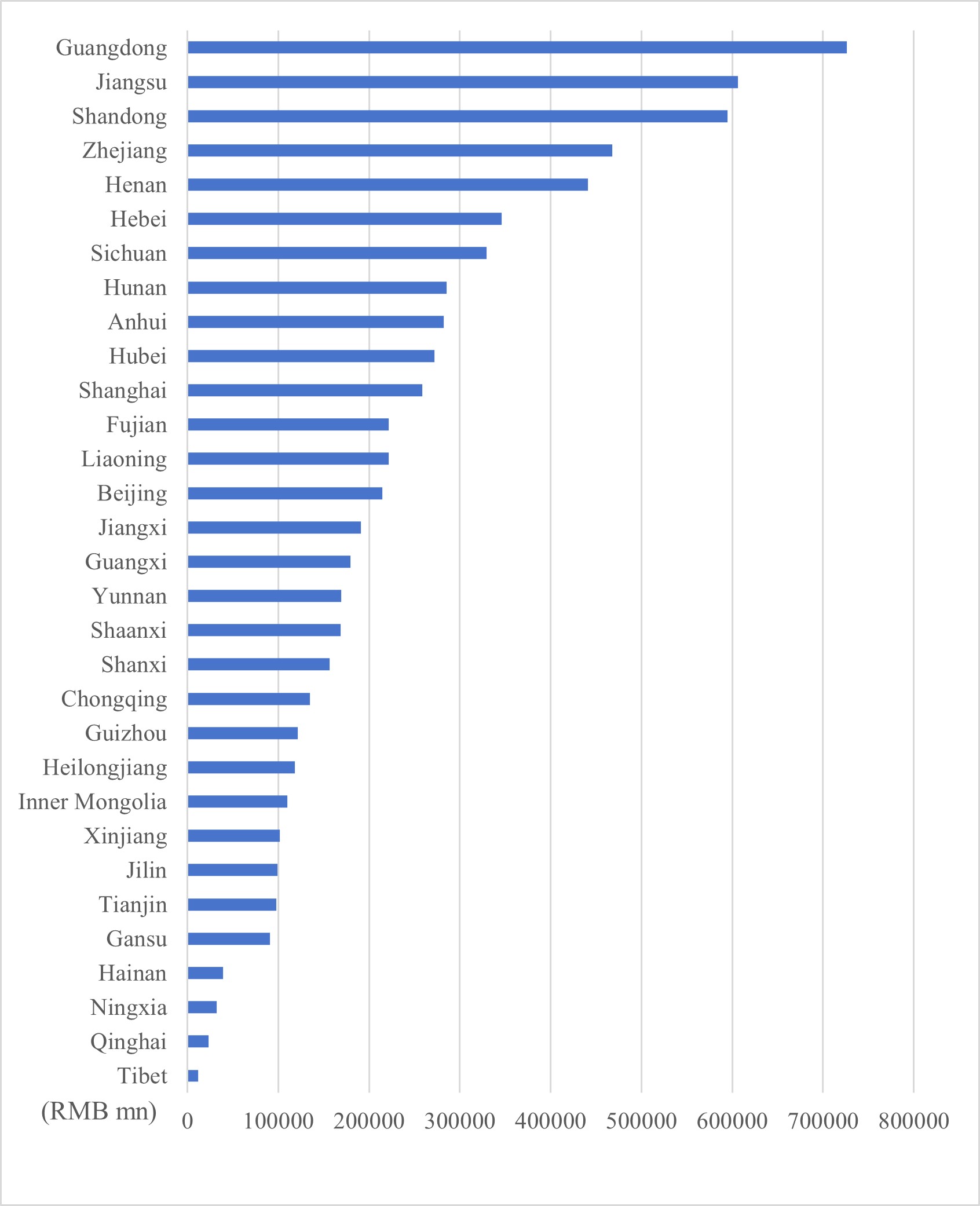

Supplement: Supplementary file 1 [file Data_Sheet_1.zip › High definition image materials-2 (including all sample years)/Health Economic Losses Caused by PM2.5 and O3 Pollution/AHC/O3 0 Health Economic Losses.jpg]

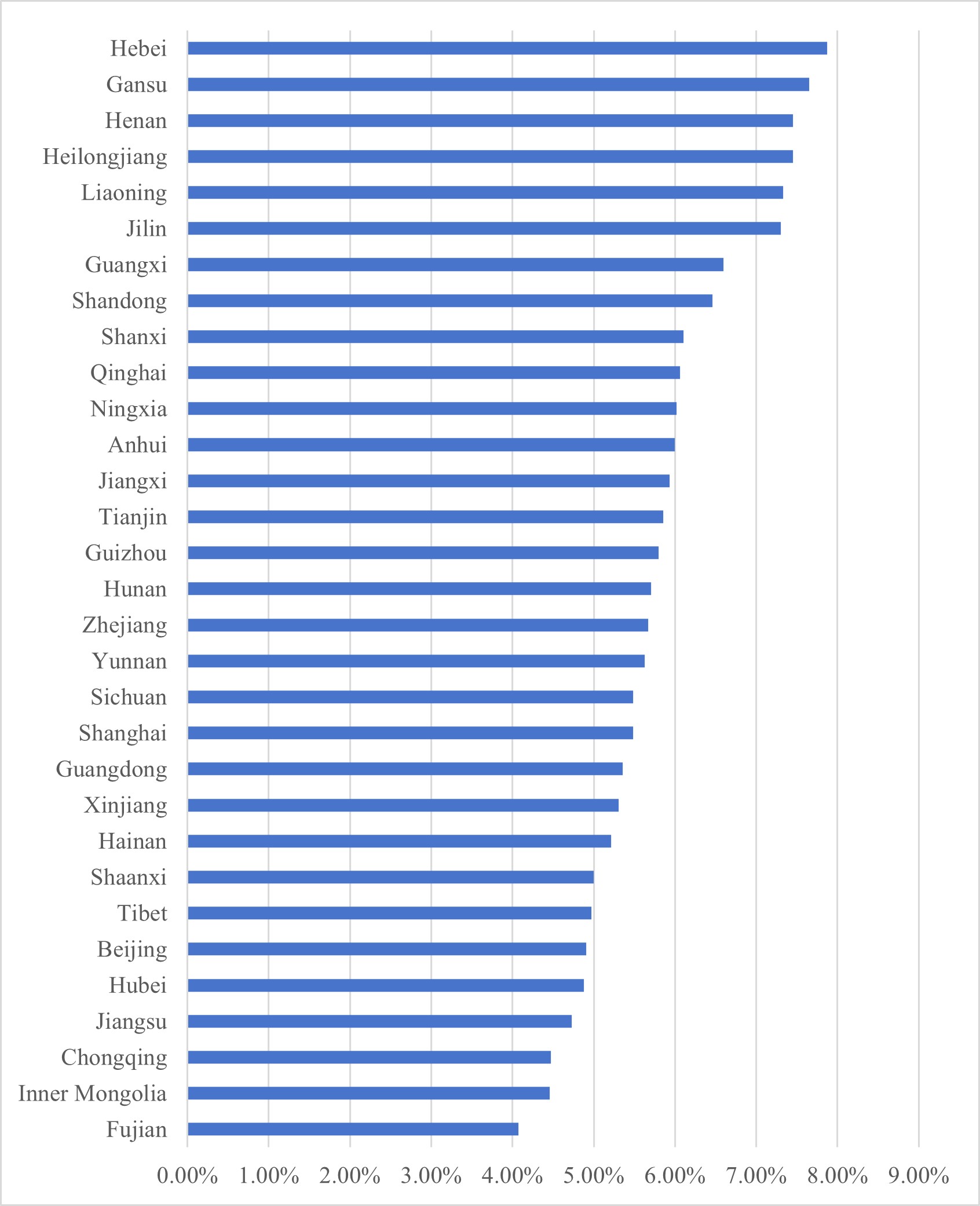

Supplement: Supplementary file 1 [file Data_Sheet_1.zip › High definition image materials-2 (including all sample years)/Health Economic Losses Caused by PM2.5 and O3 Pollution/AHC/O3 0 Ranking of Cost to GDP Ratio.jpg]

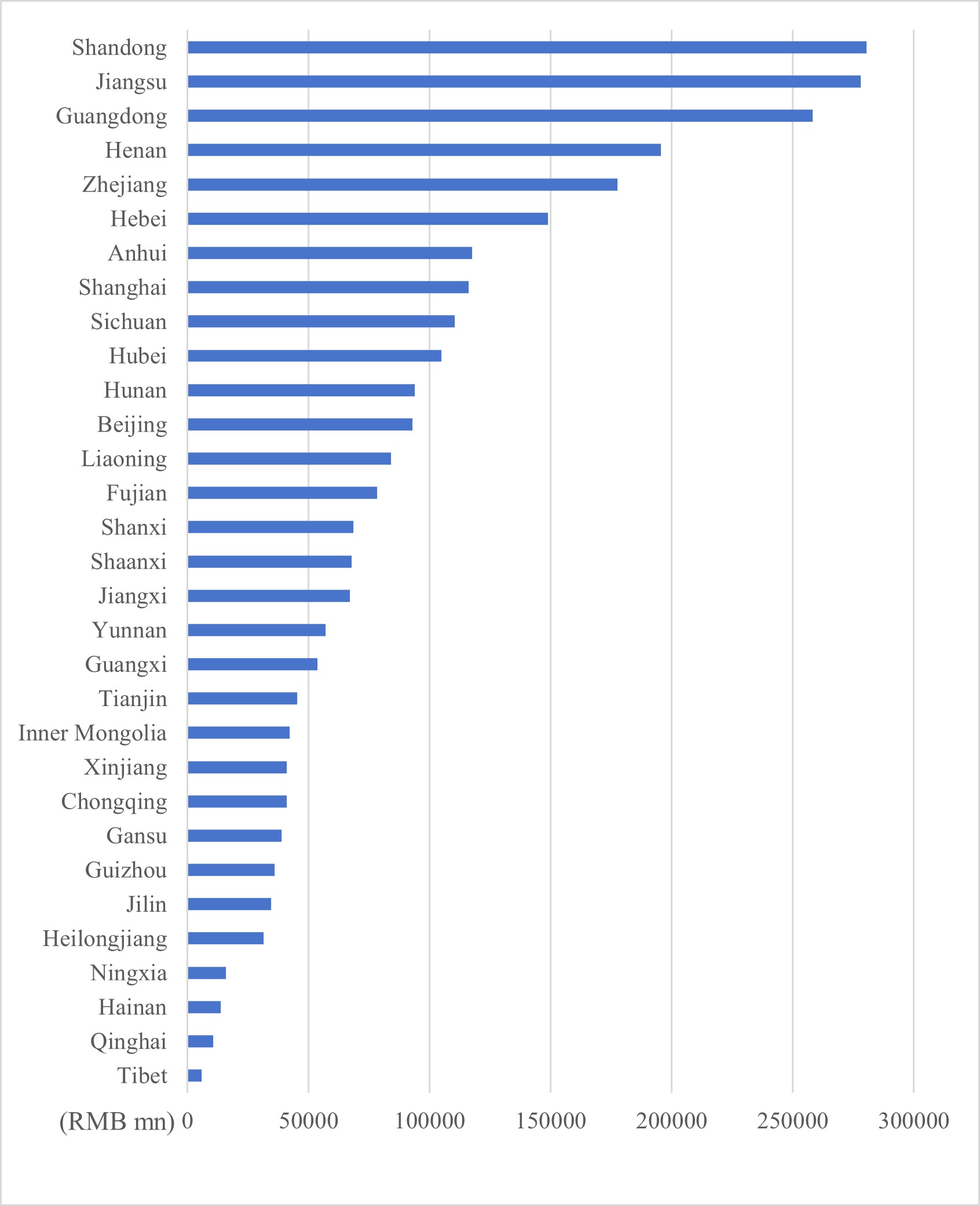

Supplement: Supplementary file 1 [file Data_Sheet_1.zip › High definition image materials-2 (including all sample years)/Health Economic Losses Caused by PM2.5 and O3 Pollution/AHC/O3 60 Health Economic Losses.jpg]

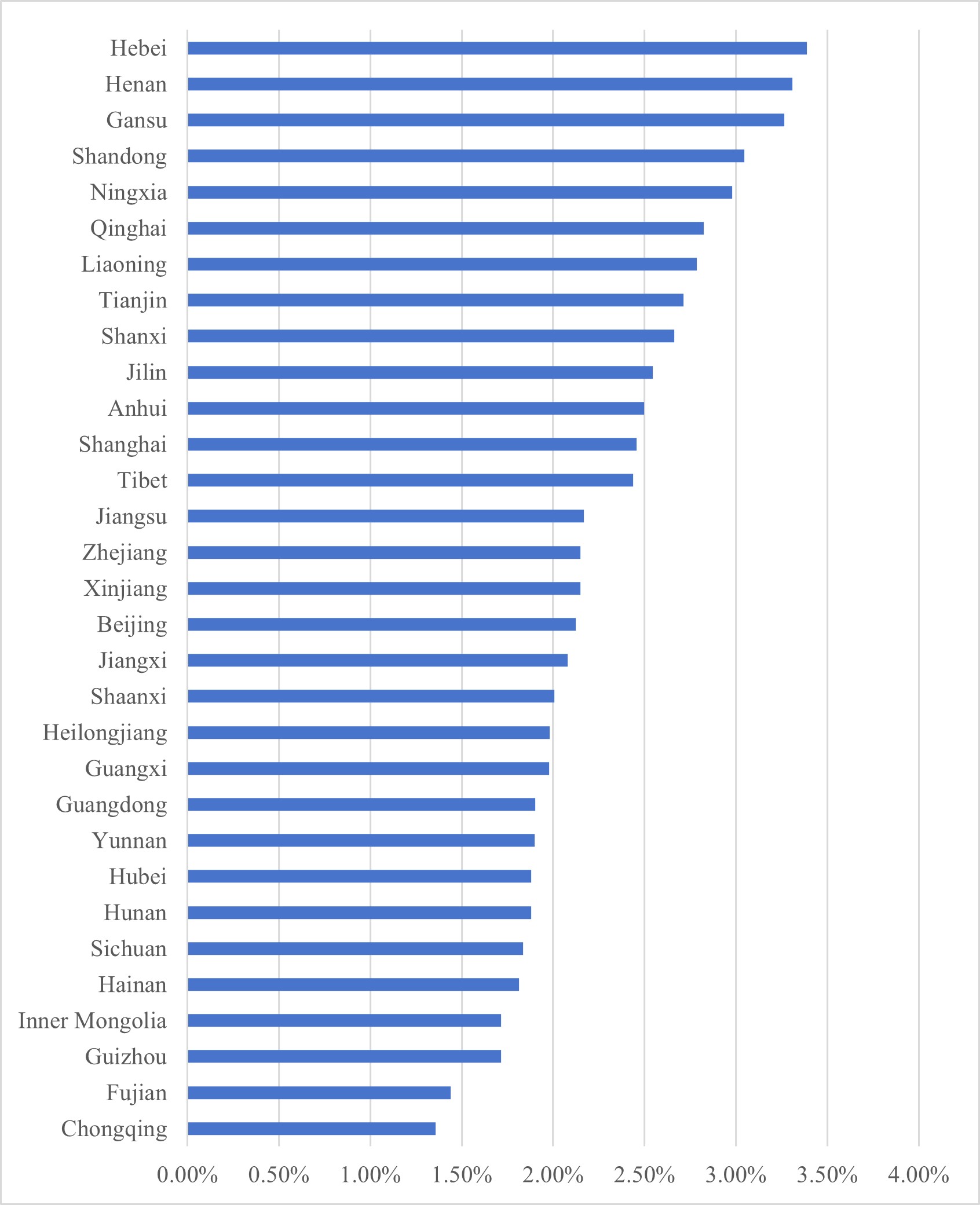

Supplement: Supplementary file 1 [file Data_Sheet_1.zip › High definition image materials-2 (including all sample years)/Health Economic Losses Caused by PM2.5 and O3 Pollution/AHC/O3 60 Ranking of Cost to GDP Ratio.jpg]

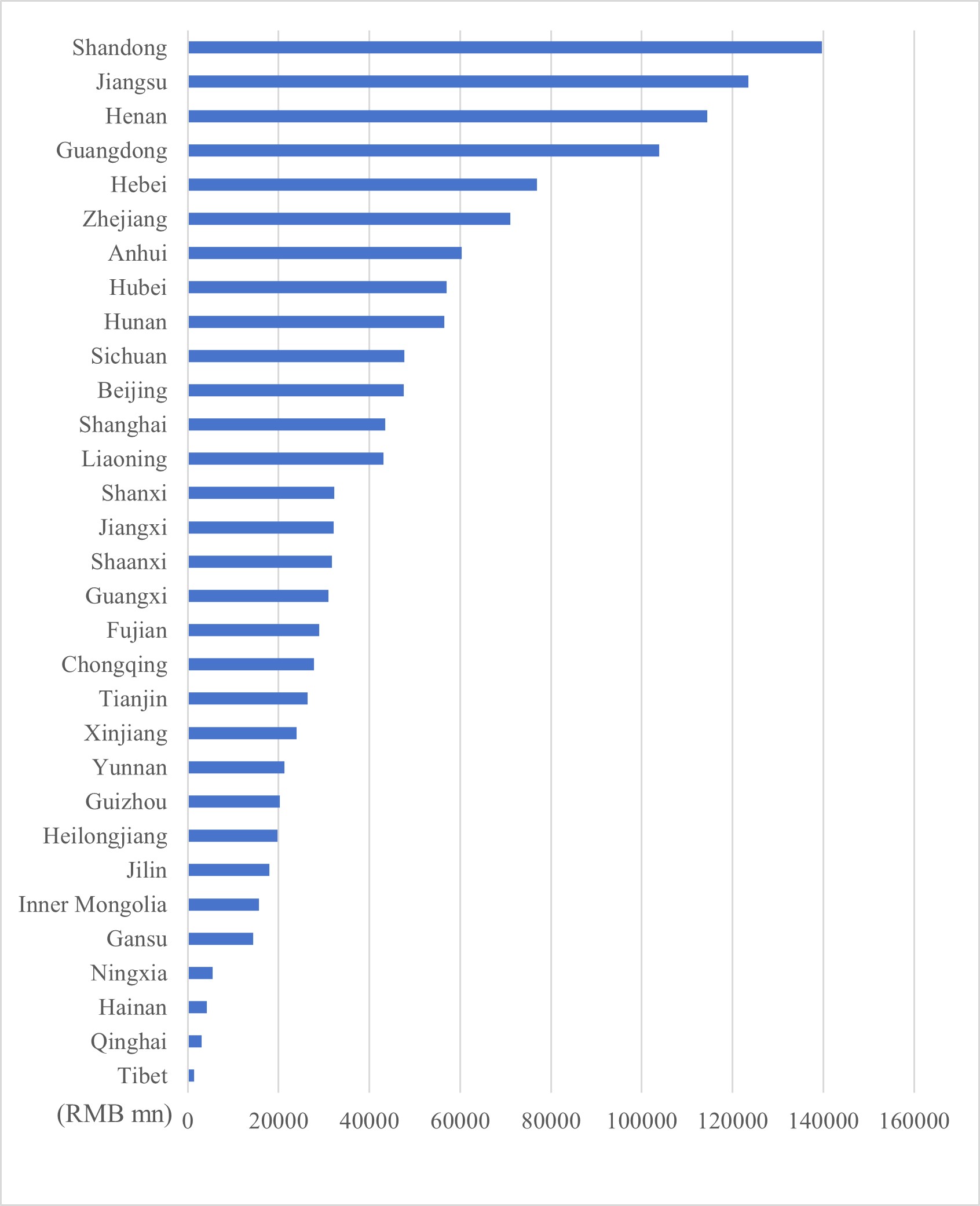

Supplement: Supplementary file 1 [file Data_Sheet_1.zip › High definition image materials-2 (including all sample years)/Health Economic Losses Caused by PM2.5 and O3 Pollution/AHC/PM2.5 0 Health Economic Losses.jpg]

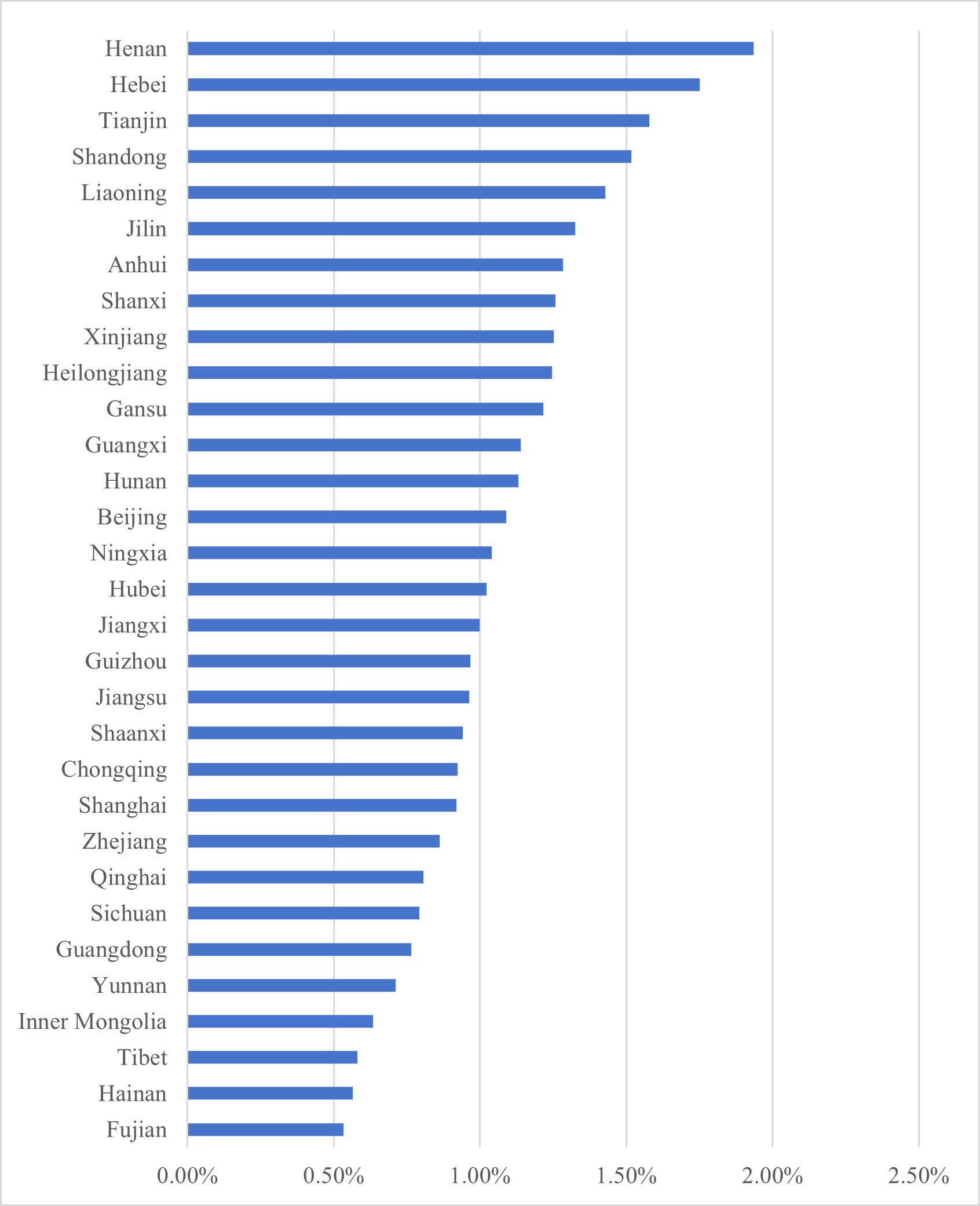

Supplement: Supplementary file 1 [file Data_Sheet_1.zip › High definition image materials-2 (including all sample years)/Health Economic Losses Caused by PM2.5 and O3 Pollution/AHC/PM2.5 0 Ranking of Cost to GDP Ratio.jpg]

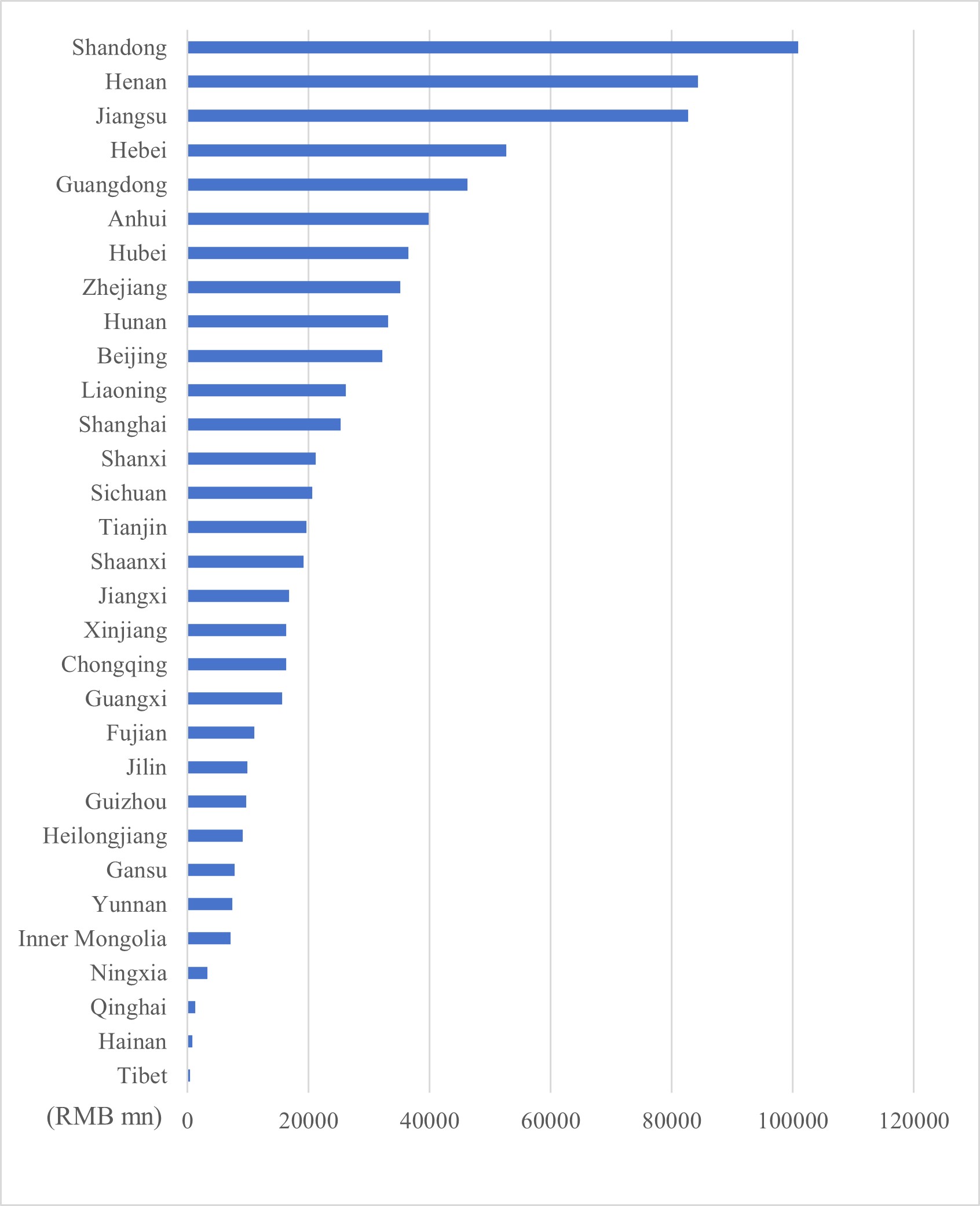

Supplement: Supplementary file 1 [file Data_Sheet_1.zip › High definition image materials-2 (including all sample years)/Health Economic Losses Caused by PM2.5 and O3 Pollution/AHC/PM2.5 15 Health Economic Losses.jpg]

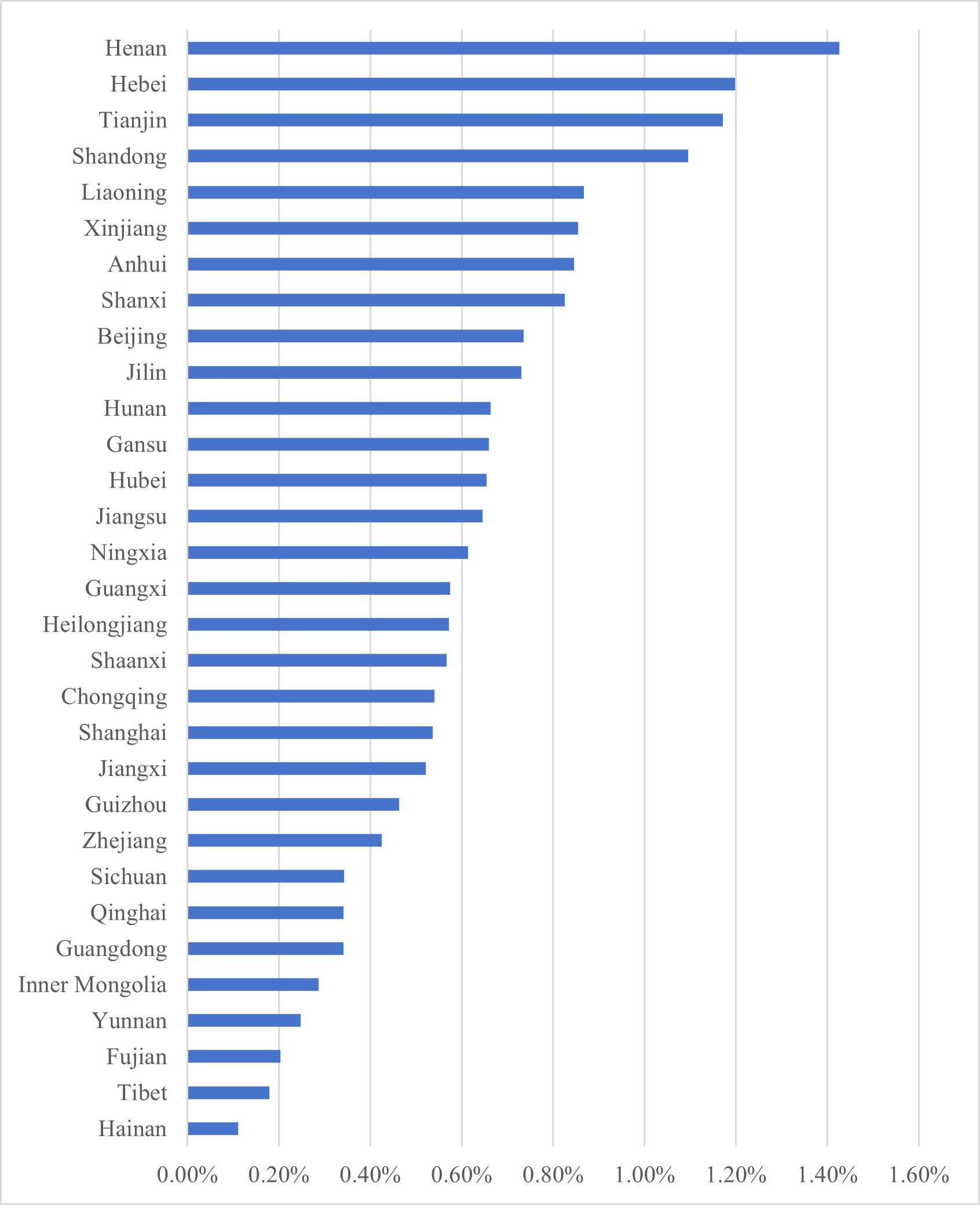

Supplement: Supplementary file 1 [file Data_Sheet_1.zip › High definition image materials-2 (including all sample years)/Health Economic Losses Caused by PM2.5 and O3 Pollution/AHC/PM2.5 15 Ranking of Cost to GDP Ratio.jpg]

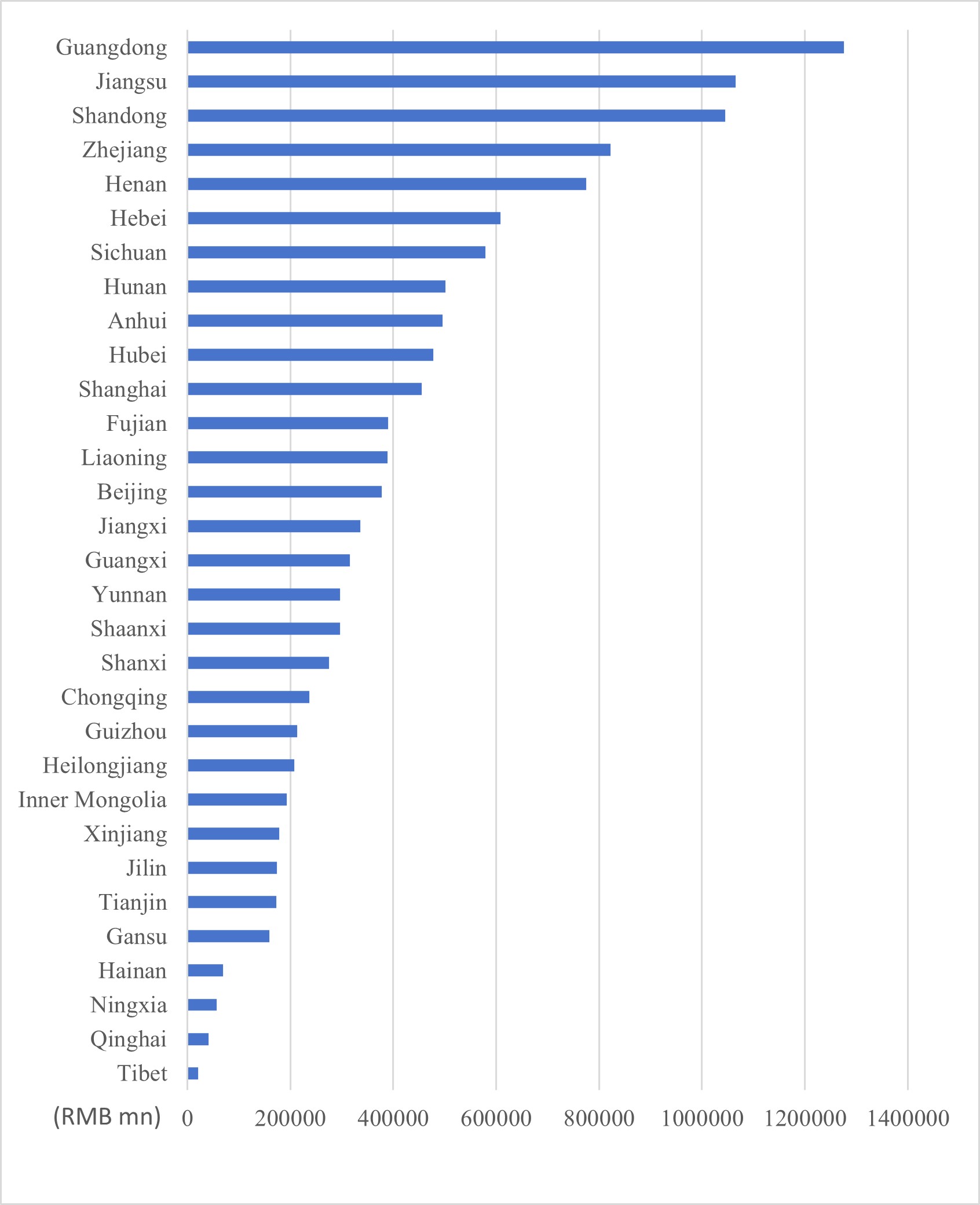

Supplement: Supplementary file 1 [file Data_Sheet_1.zip › High definition image materials-2 (including all sample years)/Health Economic Losses Caused by PM2.5 and O3 Pollution/WTP/O3 0 Health Economic Losses.jpg]

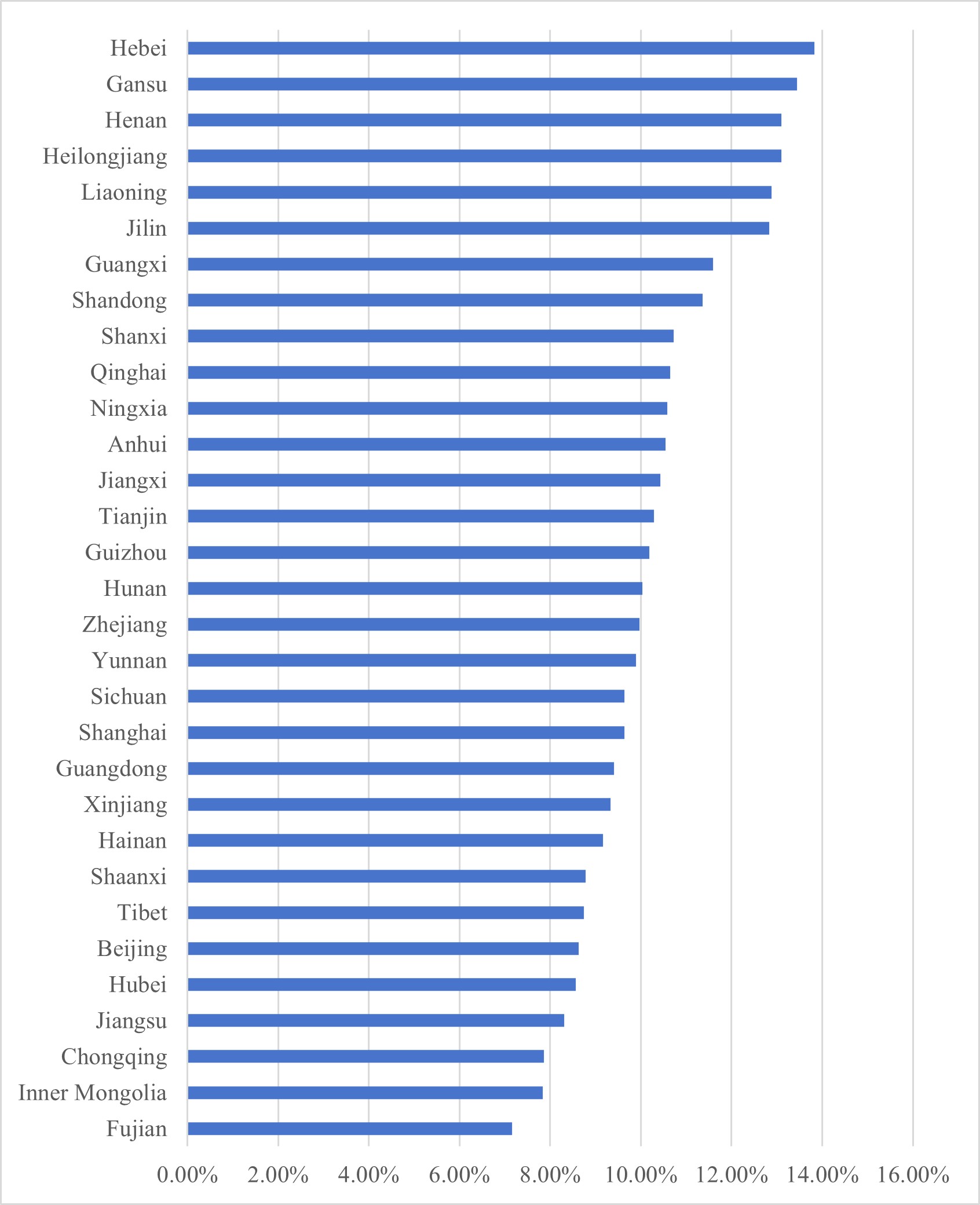

Supplement: Supplementary file 1 [file Data_Sheet_1.zip › High definition image materials-2 (including all sample years)/Health Economic Losses Caused by PM2.5 and O3 Pollution/WTP/O3 0 Ranking of Cost to GDP Ratio.jpg]

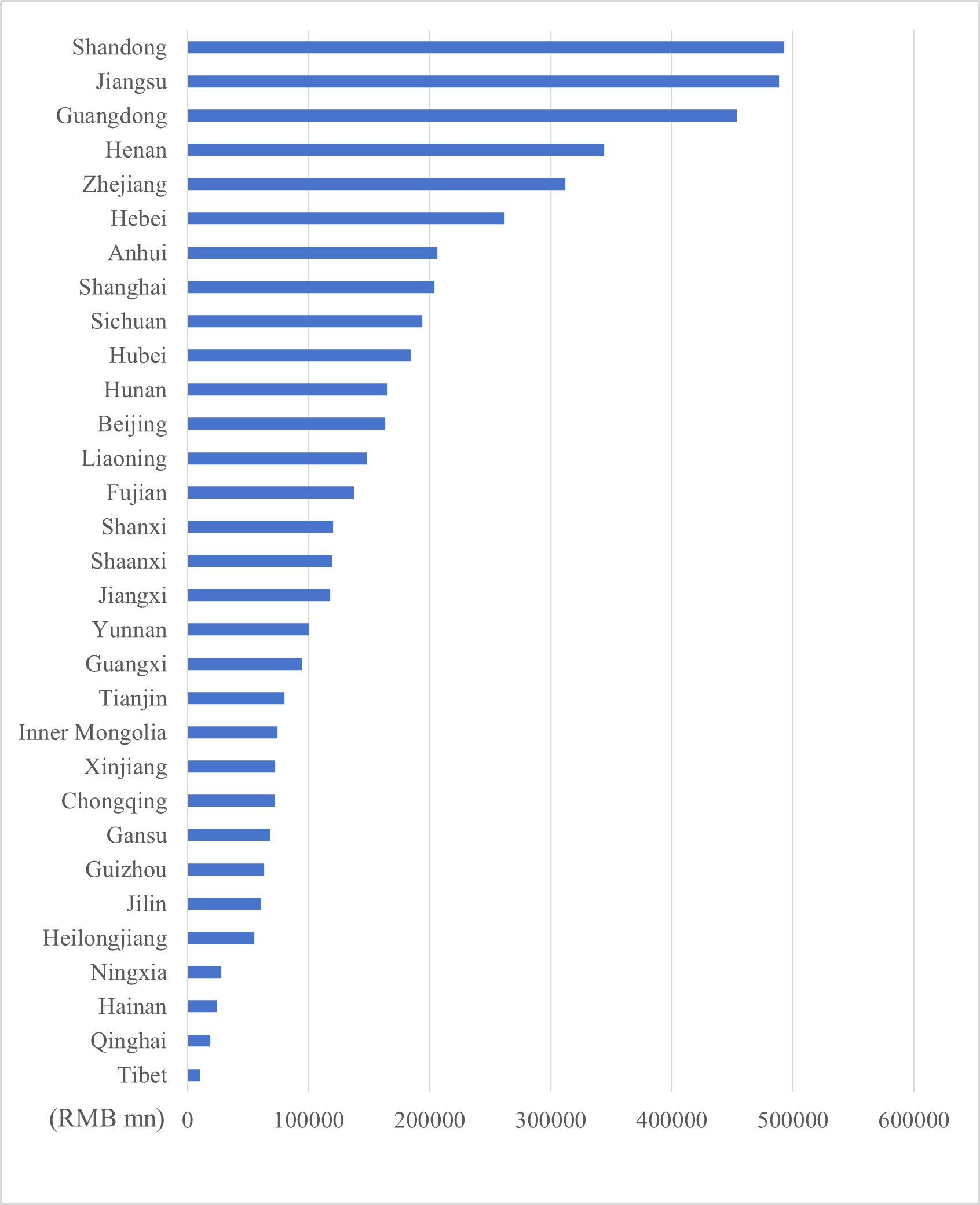

Supplement: Supplementary file 1 [file Data_Sheet_1.zip › High definition image materials-2 (including all sample years)/Health Economic Losses Caused by PM2.5 and O3 Pollution/WTP/O3 60 Health Economic Losses.jpg]

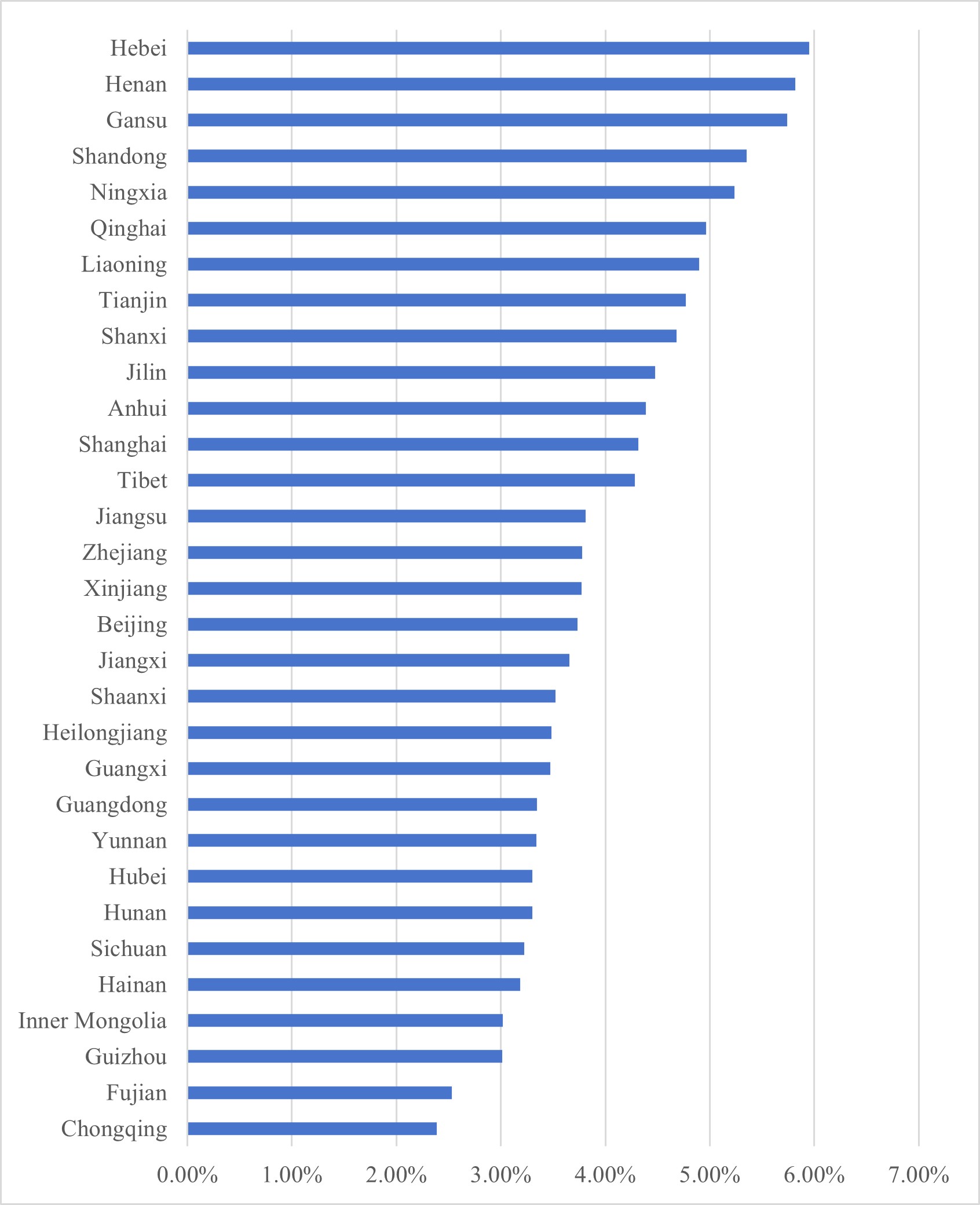

Supplement: Supplementary file 1 [file Data_Sheet_1.zip › High definition image materials-2 (including all sample years)/Health Economic Losses Caused by PM2.5 and O3 Pollution/WTP/O3 60 Ranking of Cost to GDP Ratio.jpg]

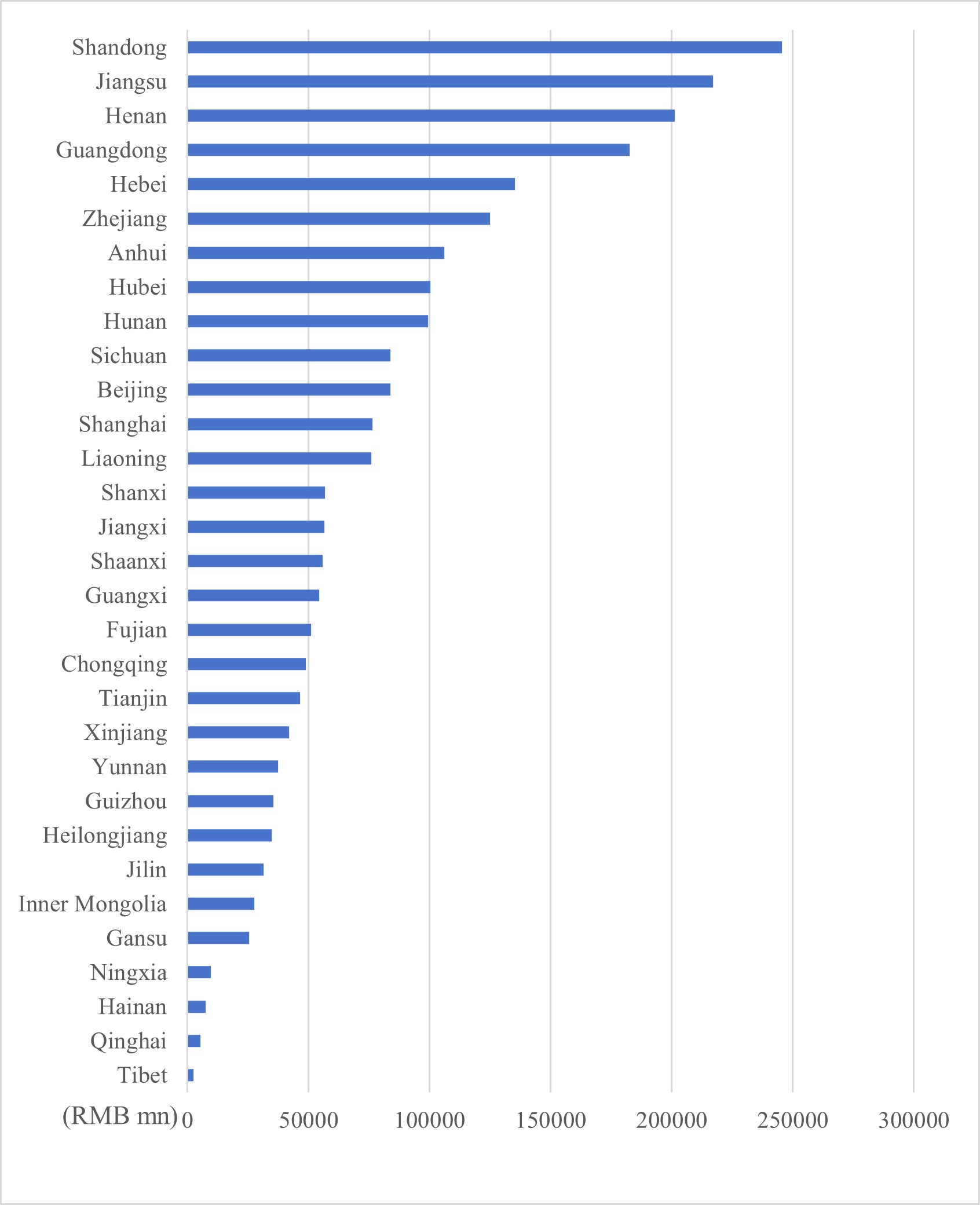

Supplement: Supplementary file 1 [file Data_Sheet_1.zip › High definition image materials-2 (including all sample years)/Health Economic Losses Caused by PM2.5 and O3 Pollution/WTP/PM2.5 0 Health Economic Losses.jpg]

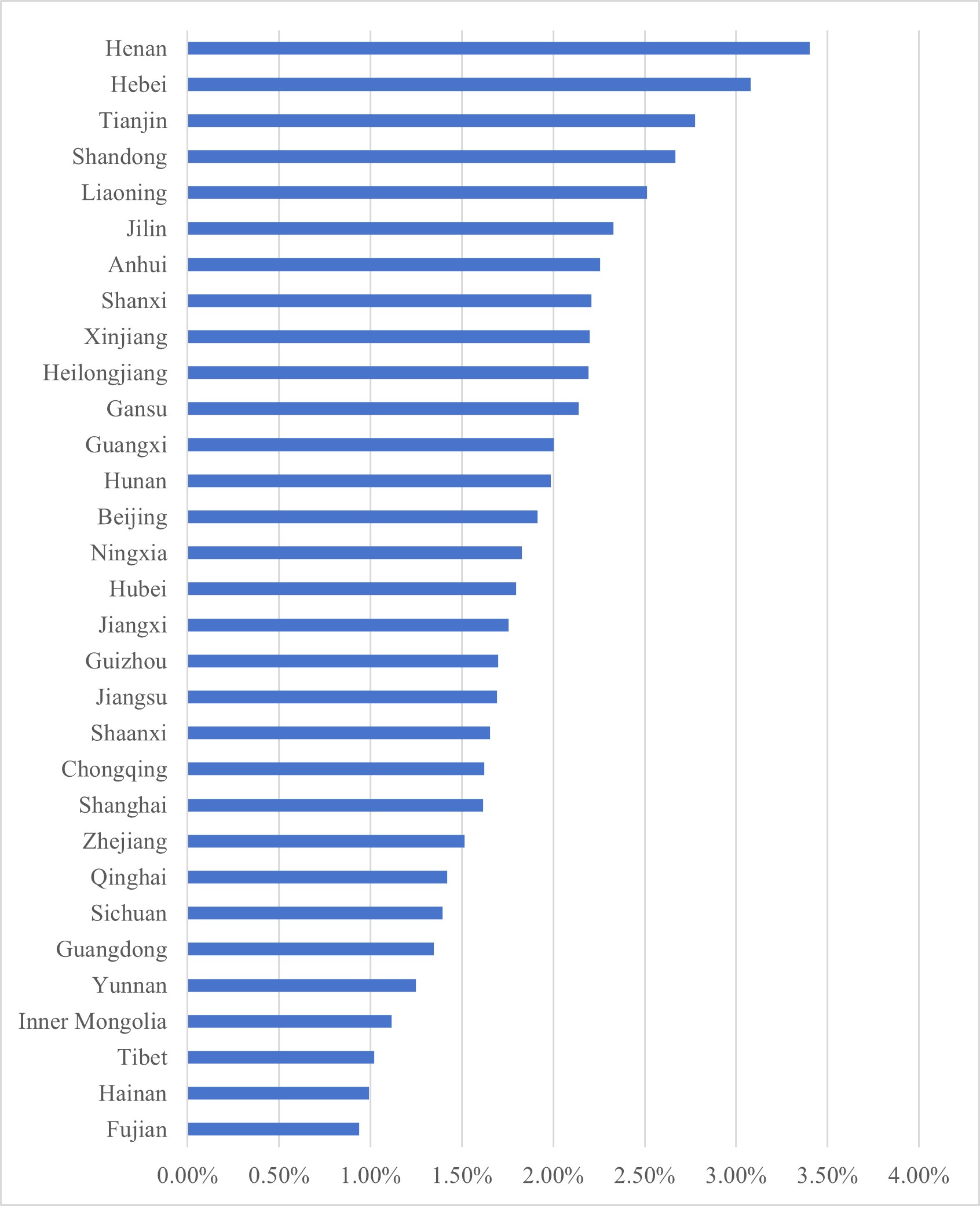

Supplement: Supplementary file 1 [file Data_Sheet_1.zip › High definition image materials-2 (including all sample years)/Health Economic Losses Caused by PM2.5 and O3 Pollution/WTP/PM2.5 0 Ranking of Cost to GDP Ratio.jpg]

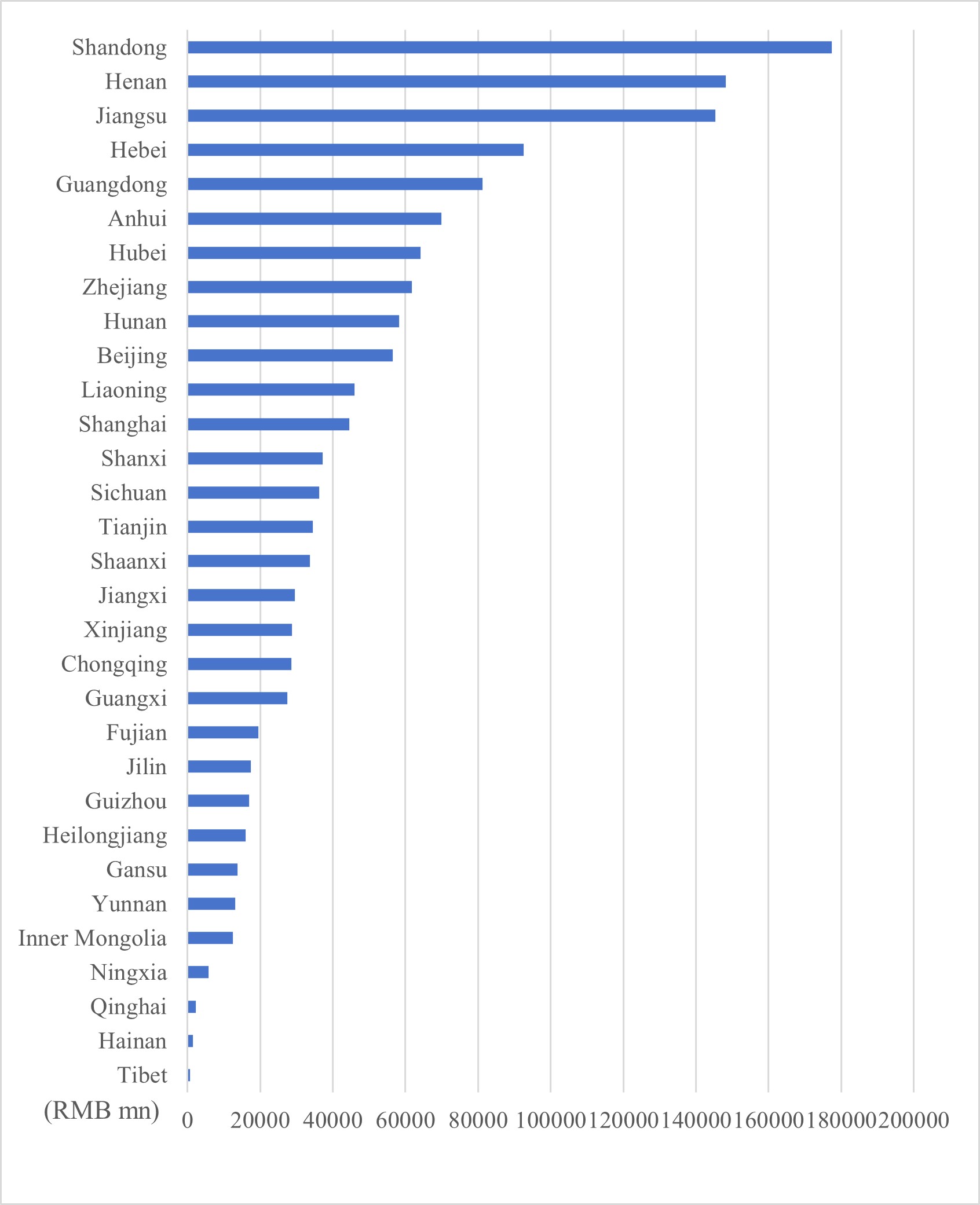

Supplement: Supplementary file 1 [file Data_Sheet_1.zip › High definition image materials-2 (including all sample years)/Health Economic Losses Caused by PM2.5 and O3 Pollution/WTP/PM2.5 15 Health Economic Losses.jpg]

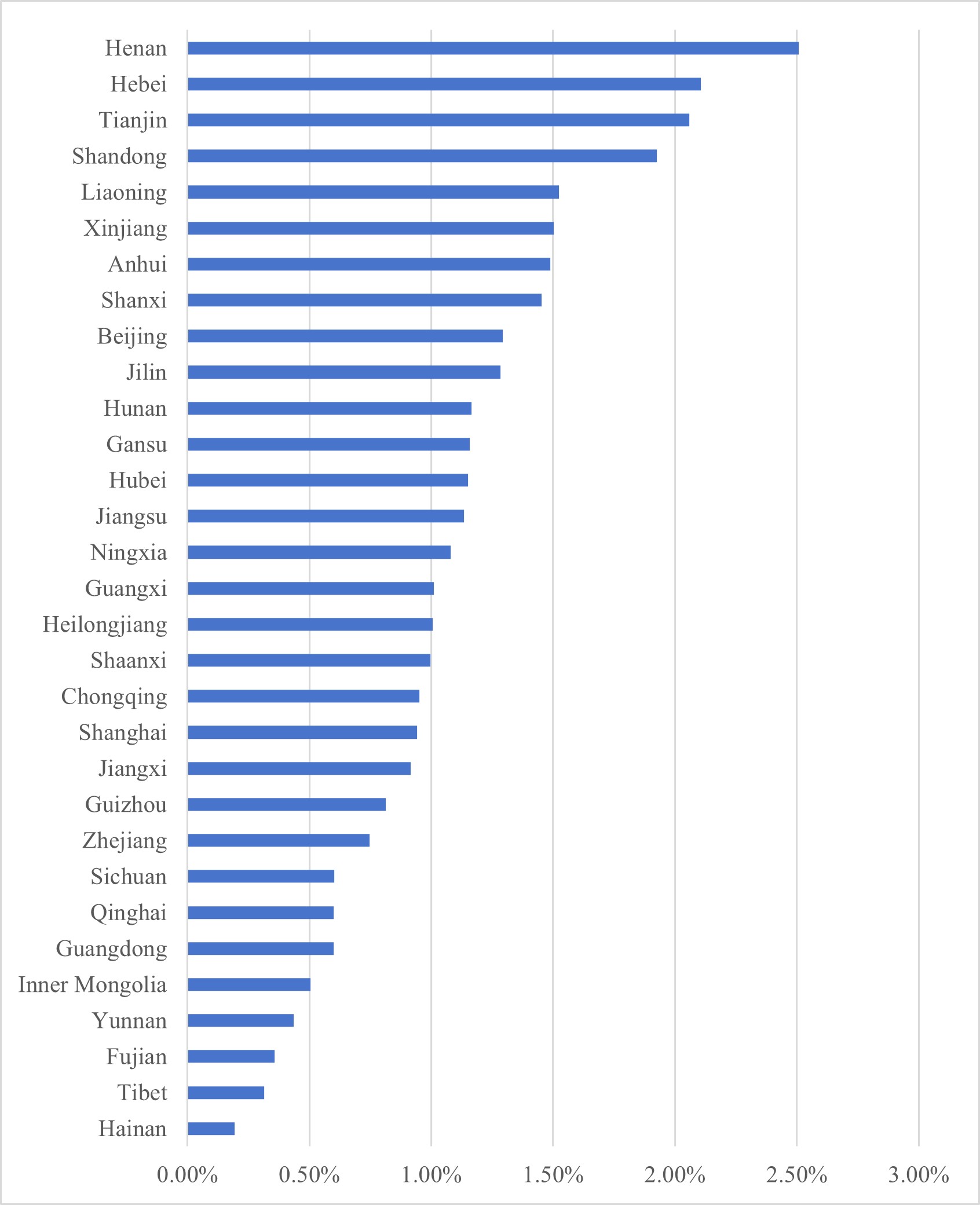

Supplement: Supplementary file 1 [file Data_Sheet_1.zip › High definition image materials-2 (including all sample years)/Health Economic Losses Caused by PM2.5 and O3 Pollution/WTP/PM2.5 15 Ranking of Cost to GDP Ratio.jpg]

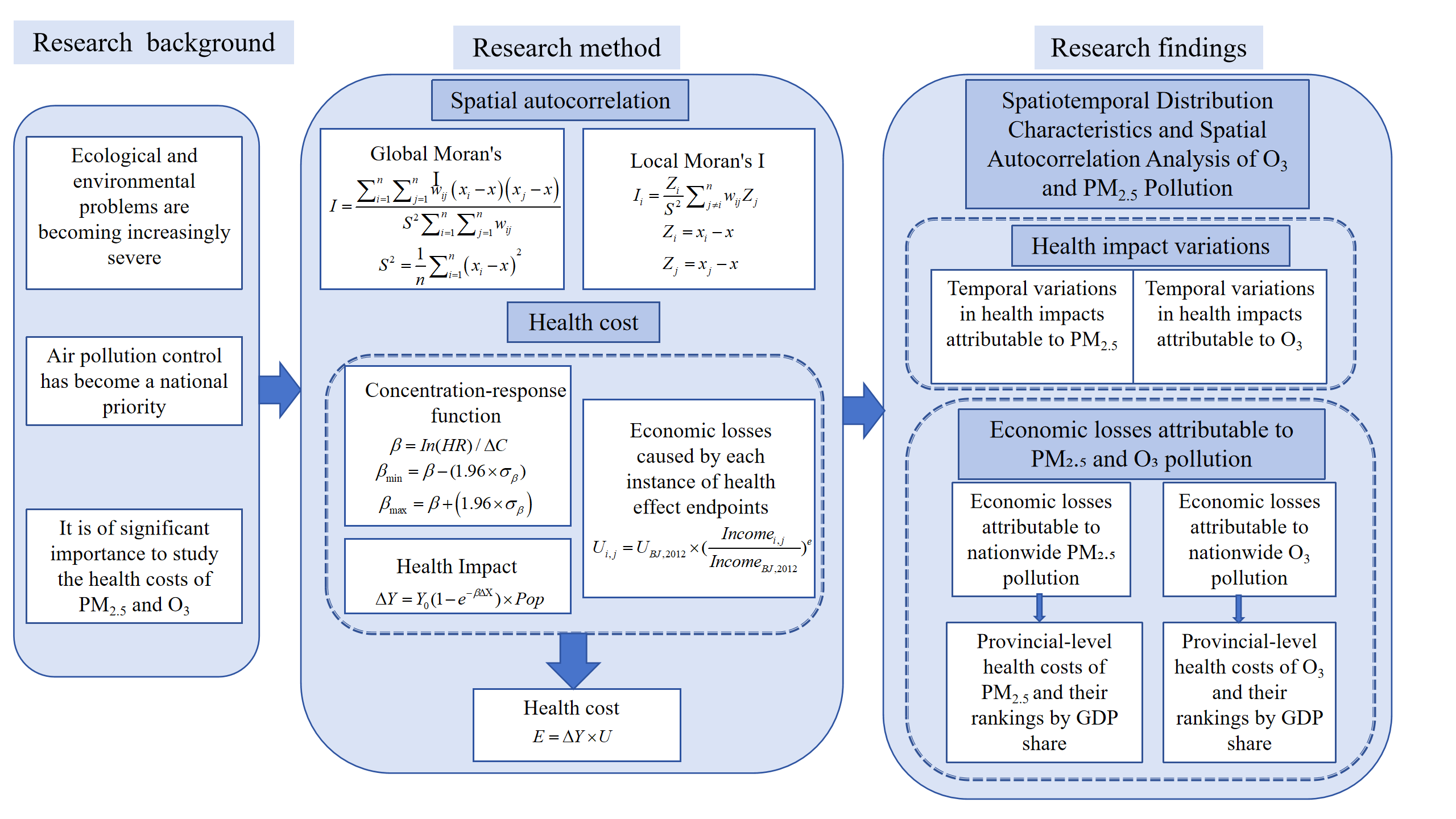

Supplement: Supplementary file 1 [file Data_Sheet_1.zip › High definition image materials-2 (including all sample years)/The technical roadmap.png]

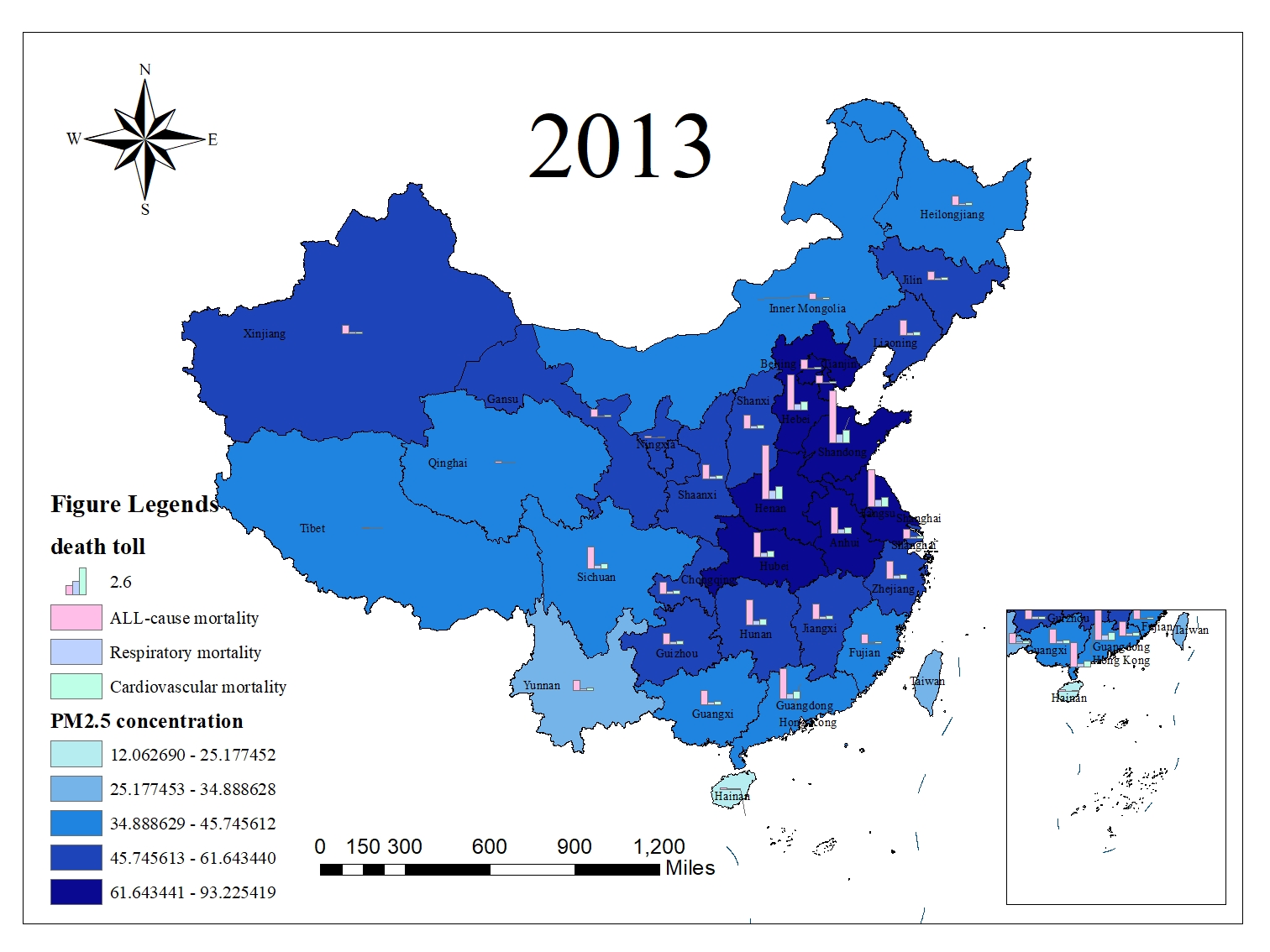

Supplement: Supplementary file 2 [file Data_Sheet_2.zip › PM2.5/PM2.5 0μgm3/2013.jpg]

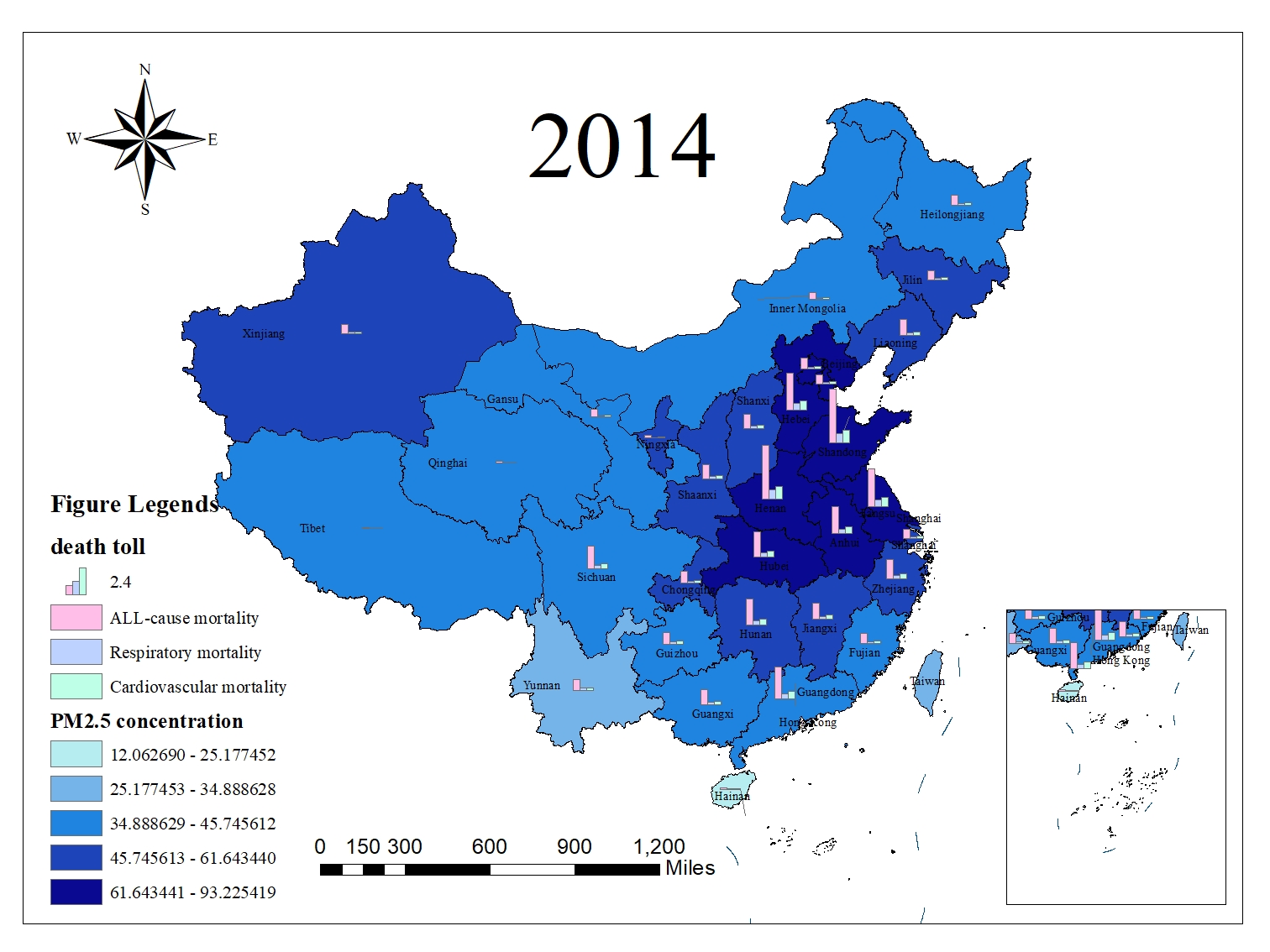

Supplement: Supplementary file 2 [file Data_Sheet_2.zip › PM2.5/PM2.5 0μgm3/2014.jpg]

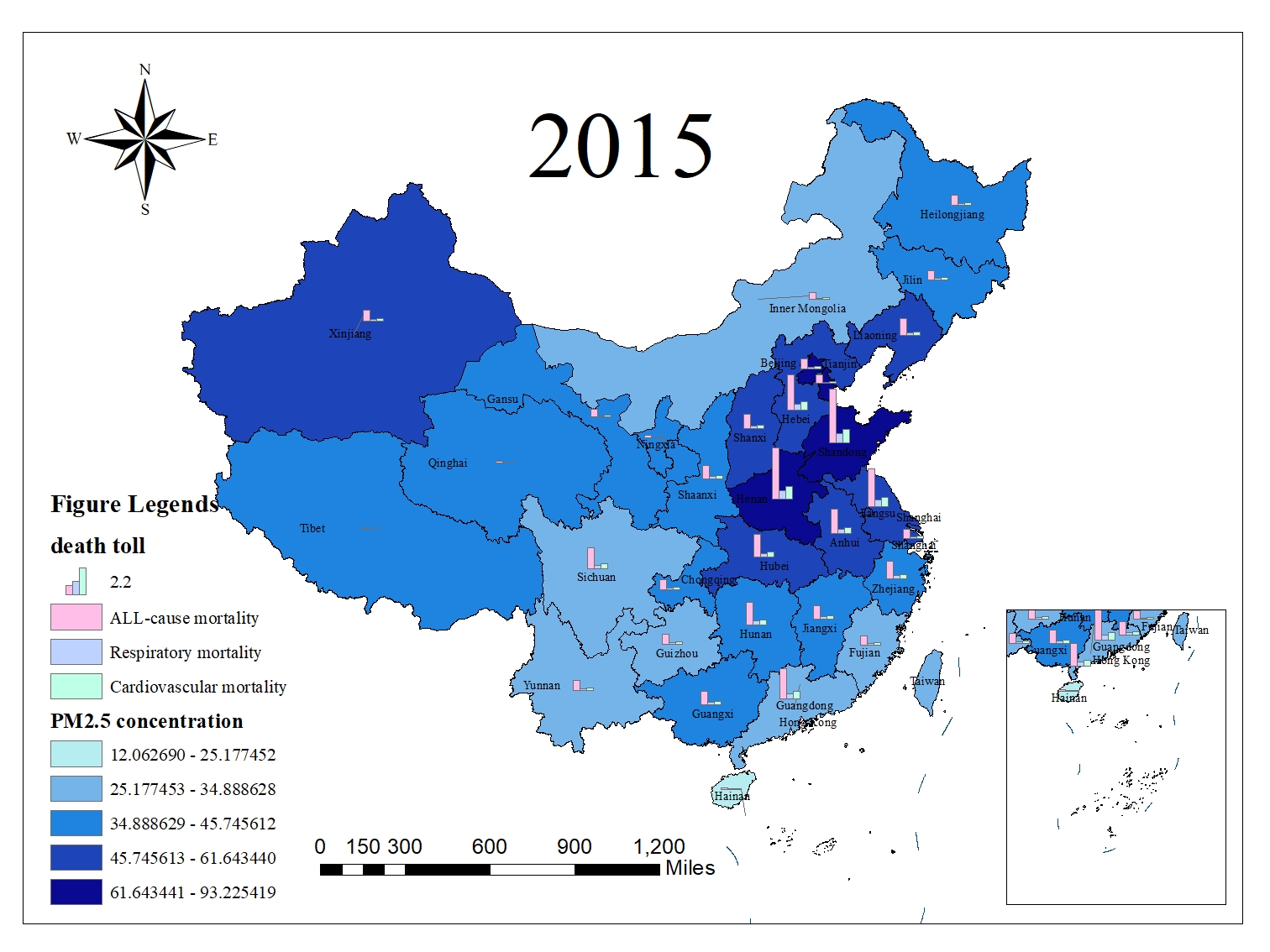

Supplement: Supplementary file 2 [file Data_Sheet_2.zip › PM2.5/PM2.5 0μgm3/2015.jpg]

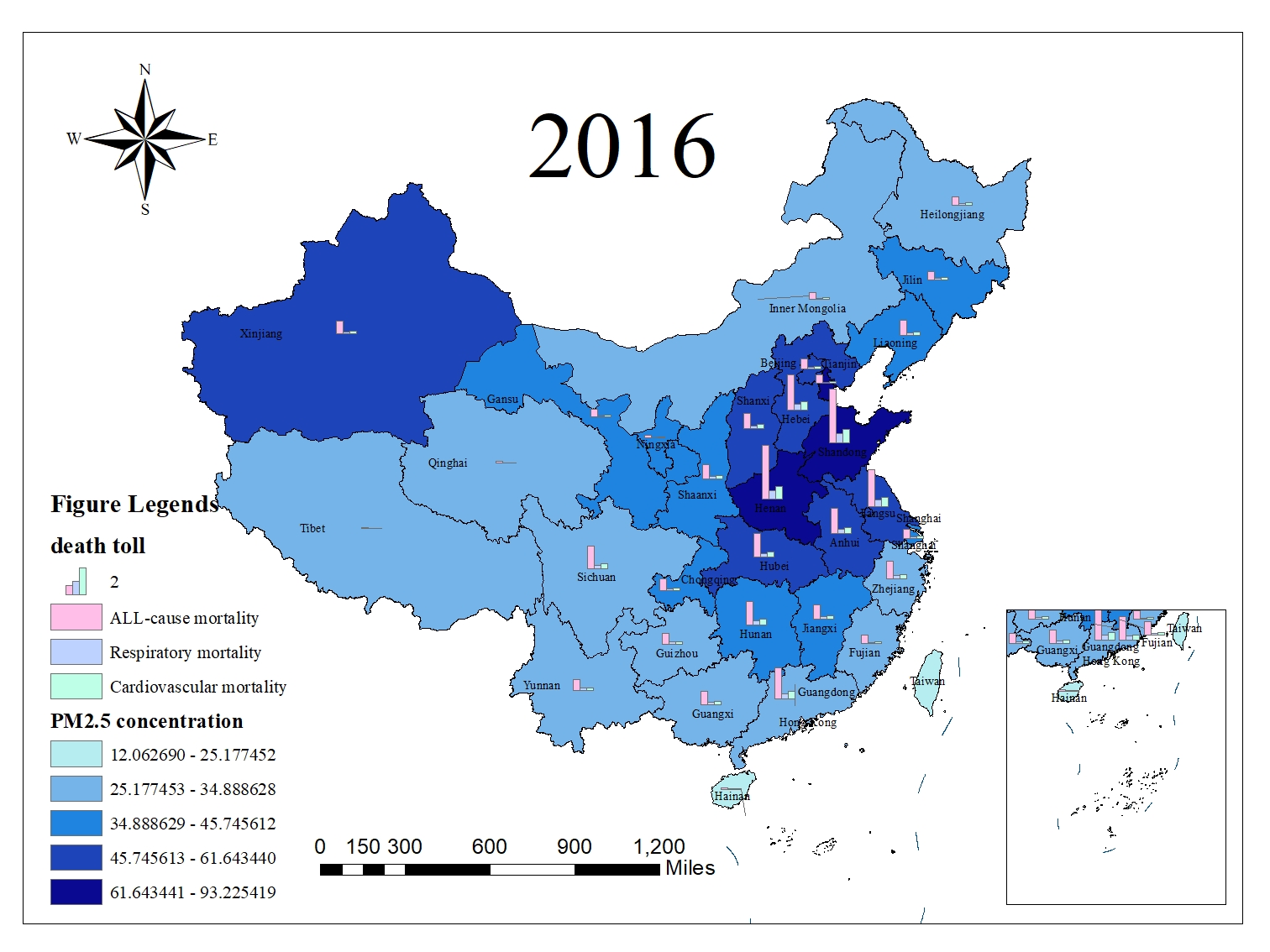

Supplement: Supplementary file 2 [file Data_Sheet_2.zip › PM2.5/PM2.5 0μgm3/2016.jpg]

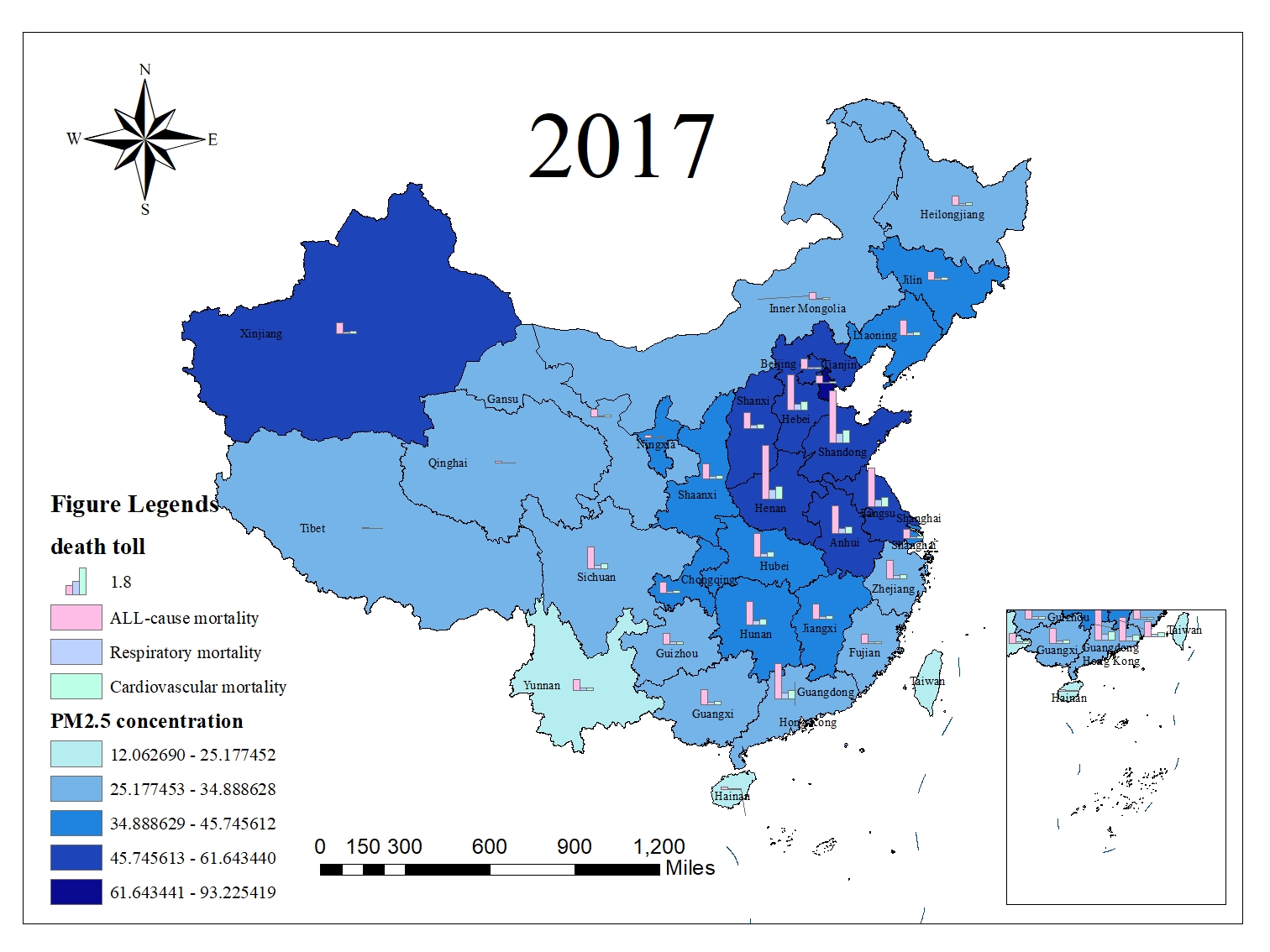

Supplement: Supplementary file 2 [file Data_Sheet_2.zip › PM2.5/PM2.5 0μgm3/2017.jpg]

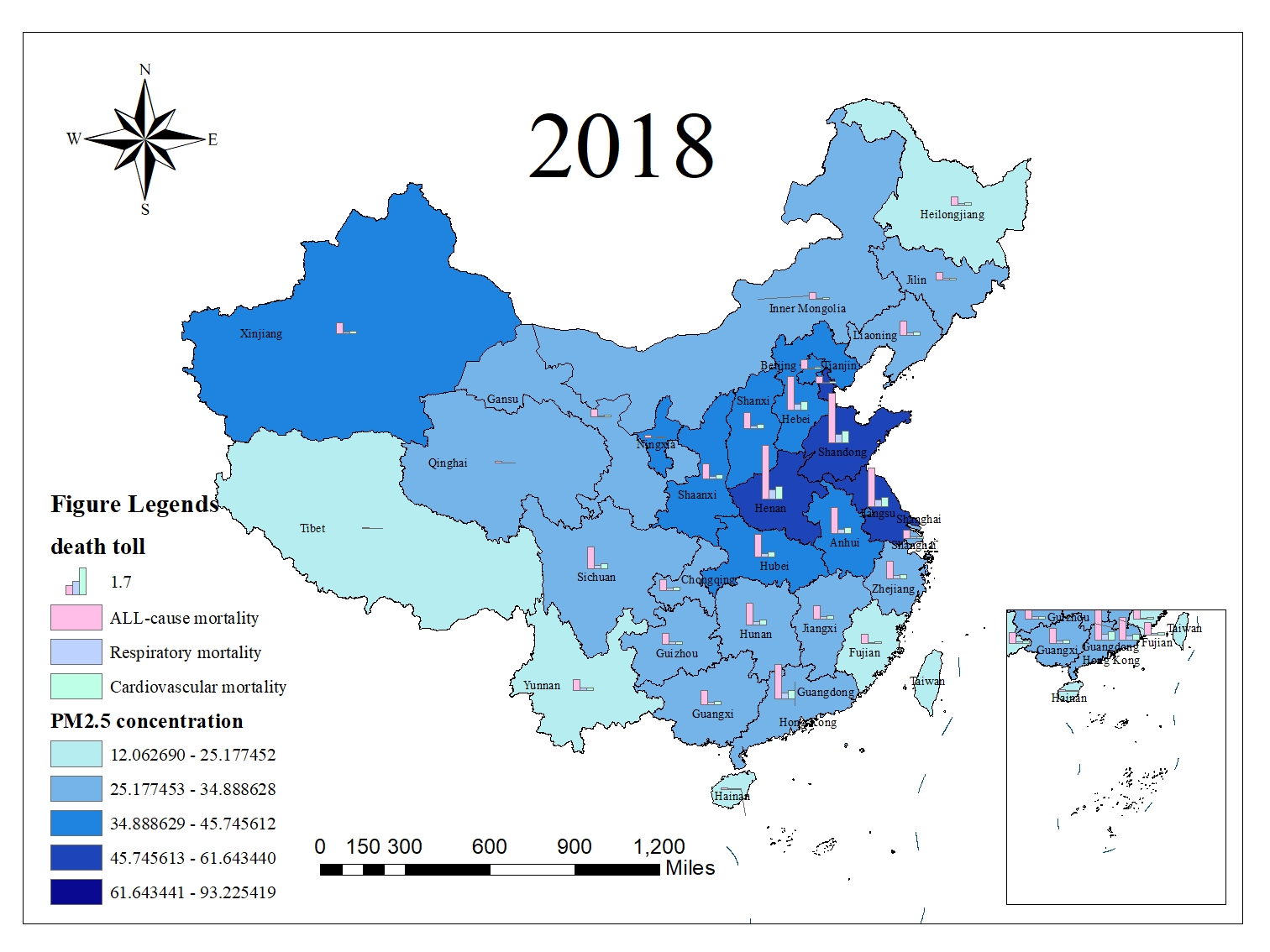

Supplement: Supplementary file 2 [file Data_Sheet_2.zip › PM2.5/PM2.5 0μgm3/2018.jpg]

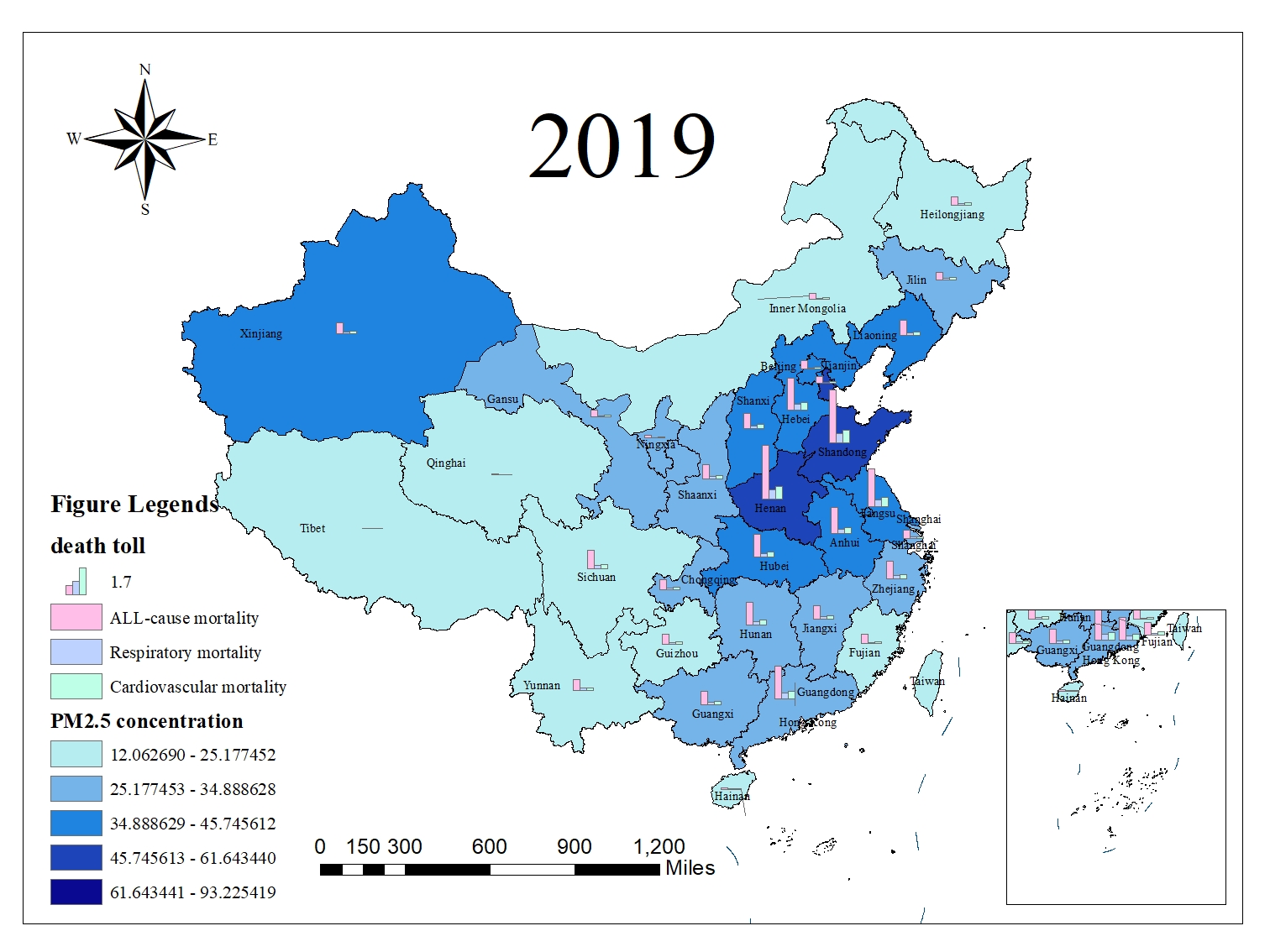

Supplement: Supplementary file 2 [file Data_Sheet_2.zip › PM2.5/PM2.5 0μgm3/2019.jpg]

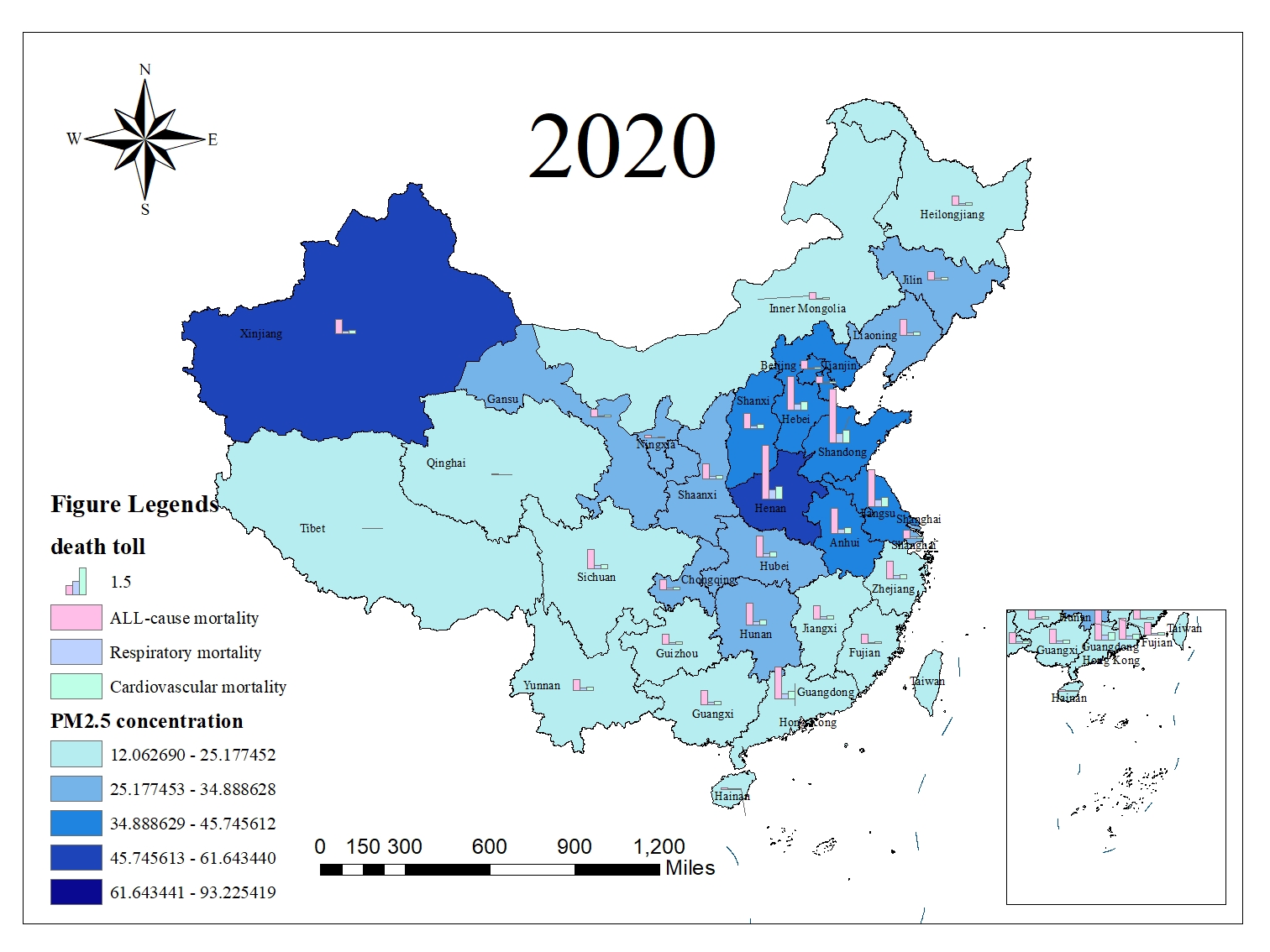

Supplement: Supplementary file 2 [file Data_Sheet_2.zip › PM2.5/PM2.5 0μgm3/2020.jpg]

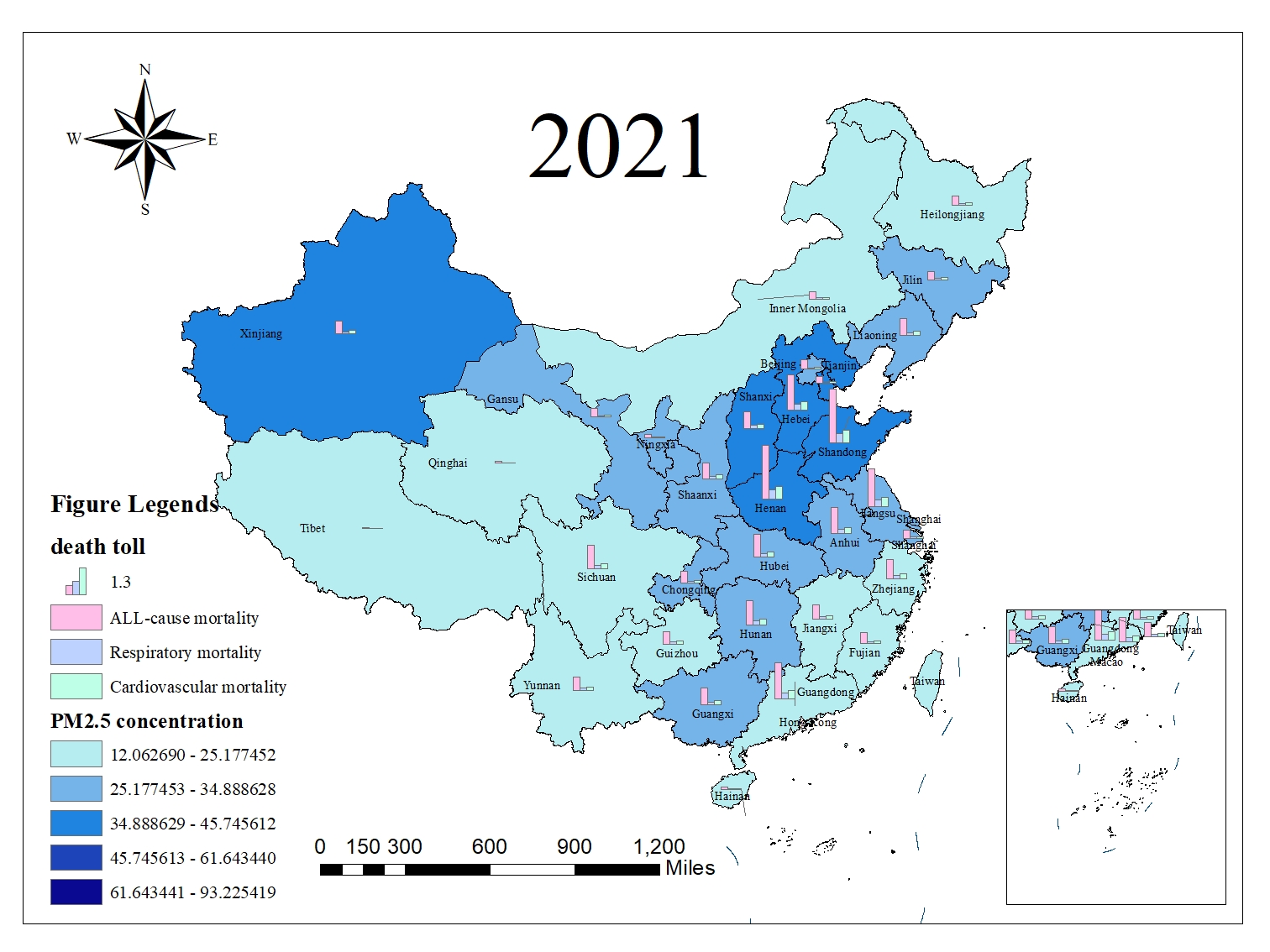

Supplement: Supplementary file 2 [file Data_Sheet_2.zip › PM2.5/PM2.5 0μgm3/2021.jpg]

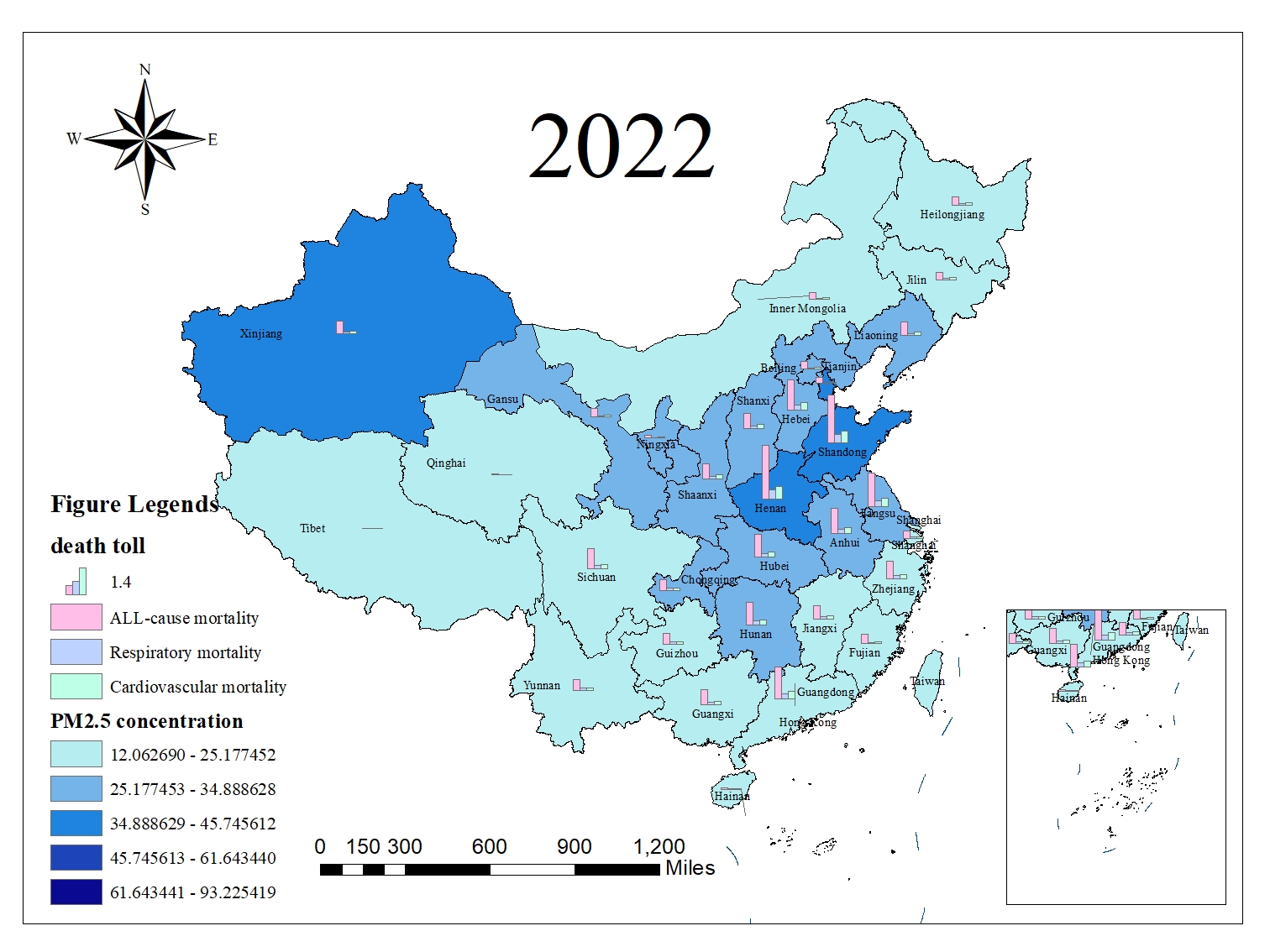

Supplement: Supplementary file 2 [file Data_Sheet_2.zip › PM2.5/PM2.5 0μgm3/2022.jpg]

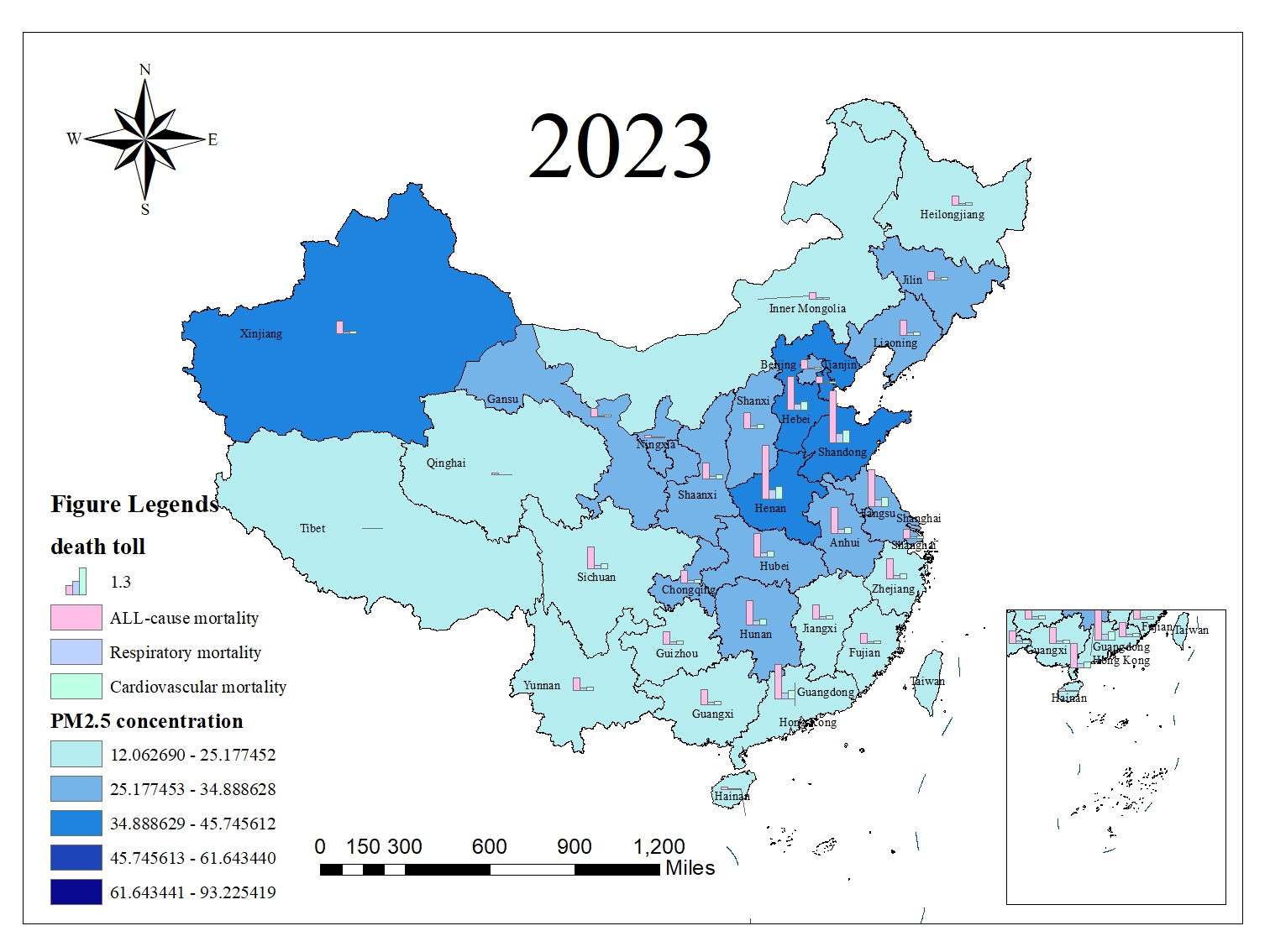

Supplement: Supplementary file 2 [file Data_Sheet_2.zip › PM2.5/PM2.5 0μgm3/2023.jpg]

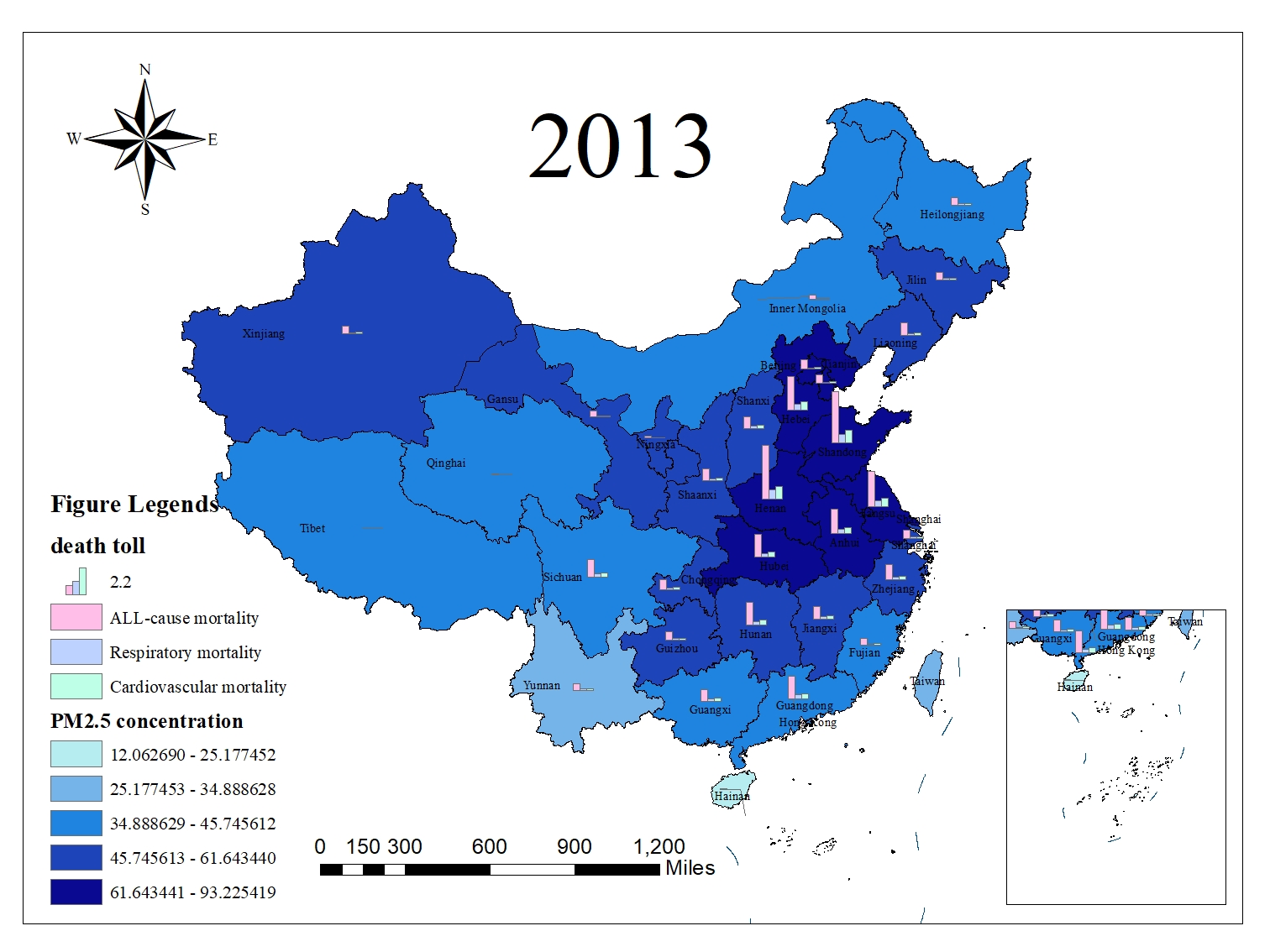

Supplement: Supplementary file 2 [file Data_Sheet_2.zip › PM2.5/PM2.5 15μgm3/2013.jpg]

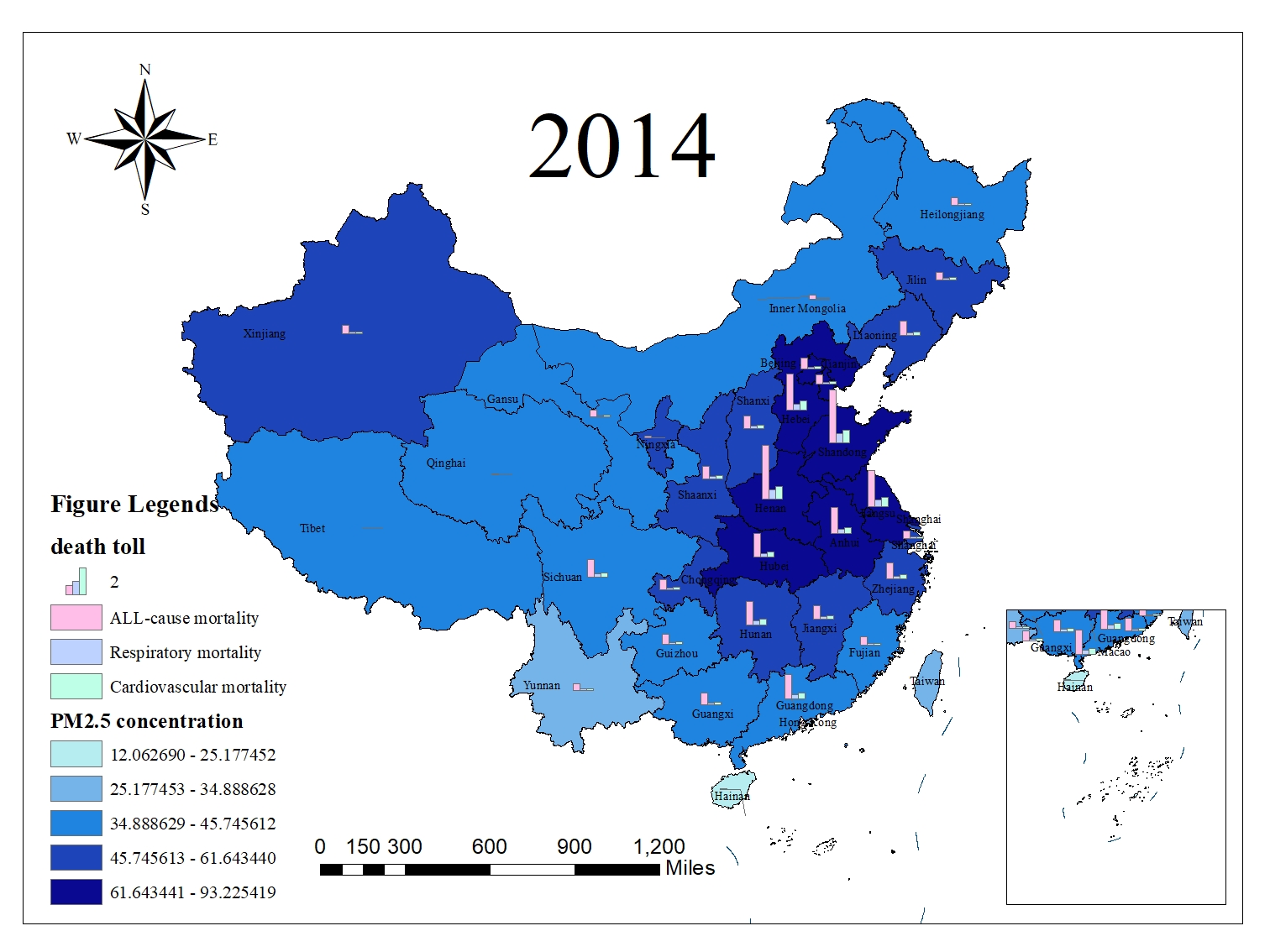

Supplement: Supplementary file 2 [file Data_Sheet_2.zip › PM2.5/PM2.5 15μgm3/2014.jpg]

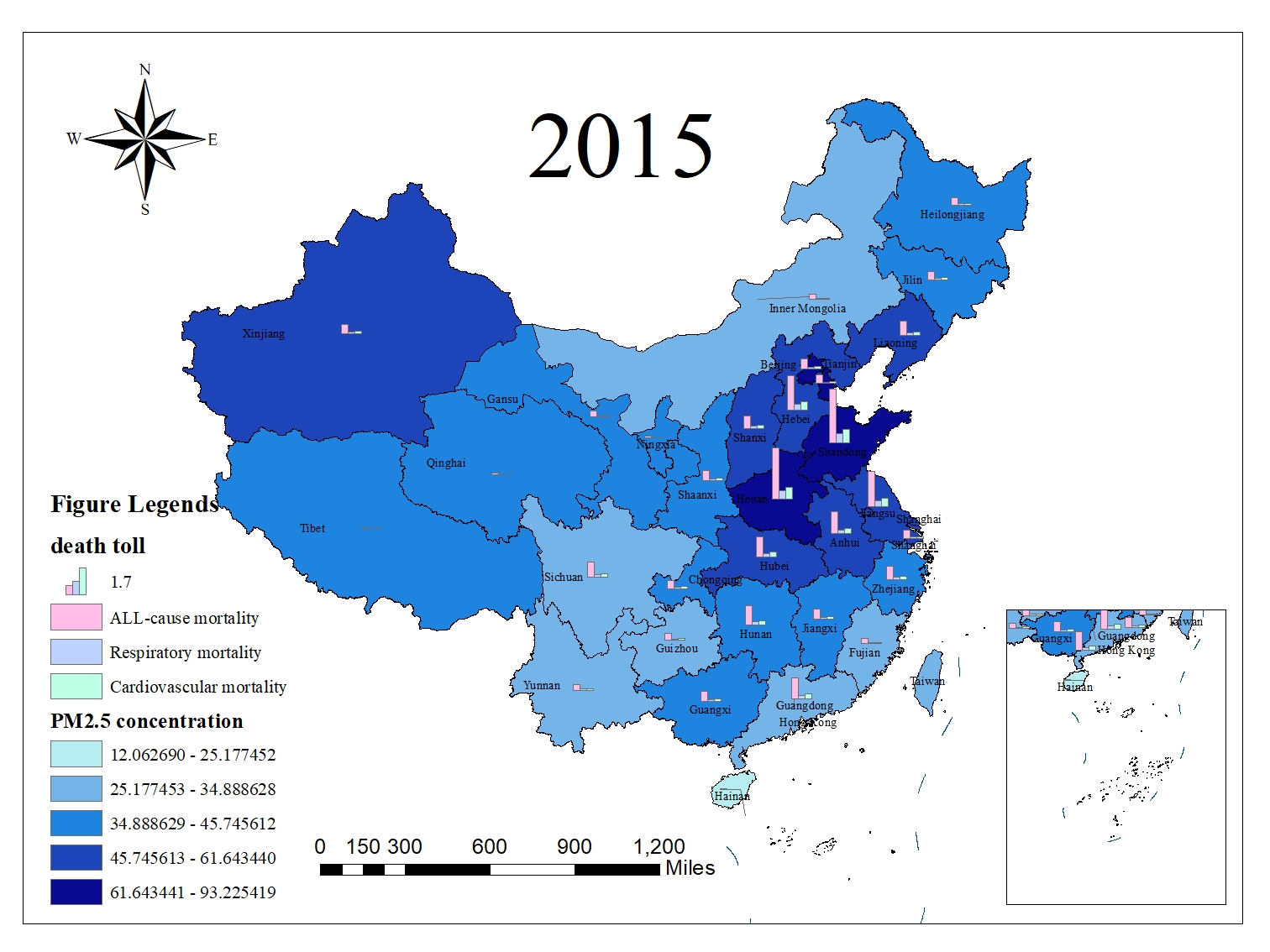

Supplement: Supplementary file 2 [file Data_Sheet_2.zip › PM2.5/PM2.5 15μgm3/2015.jpg]

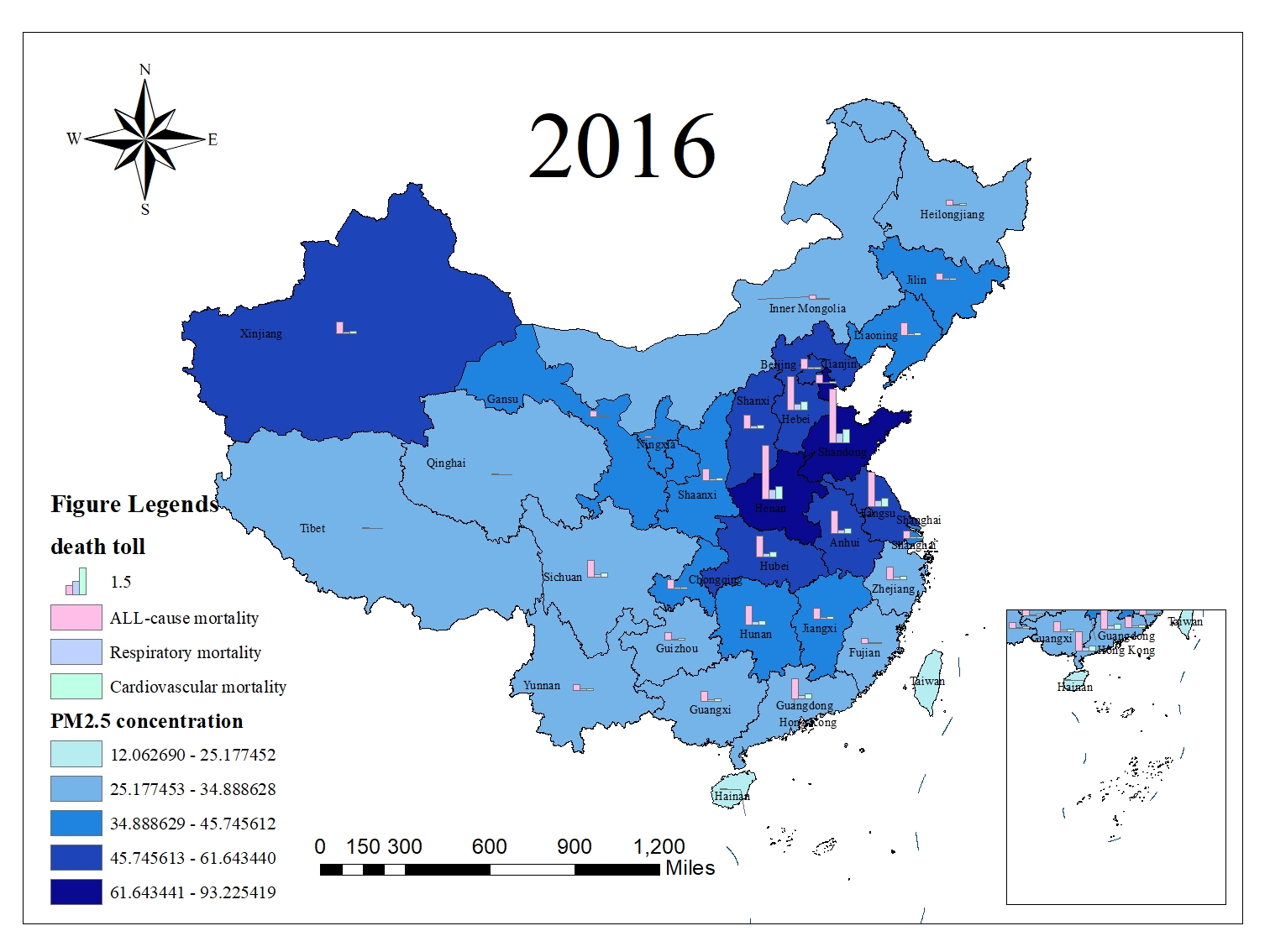

Supplement: Supplementary file 2 [file Data_Sheet_2.zip › PM2.5/PM2.5 15μgm3/2016.jpg]

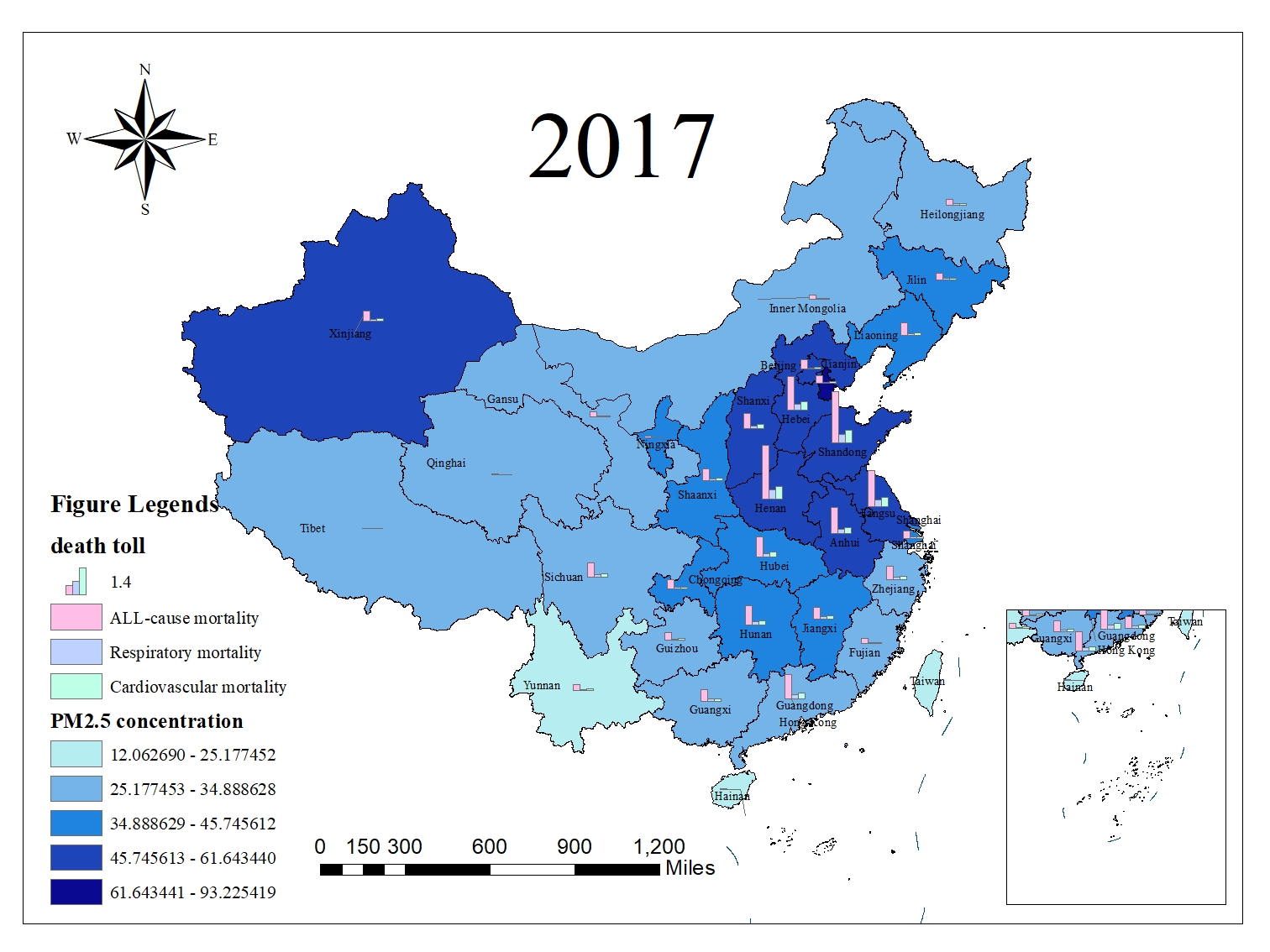

Supplement: Supplementary file 2 [file Data_Sheet_2.zip › PM2.5/PM2.5 15μgm3/2017.jpg]

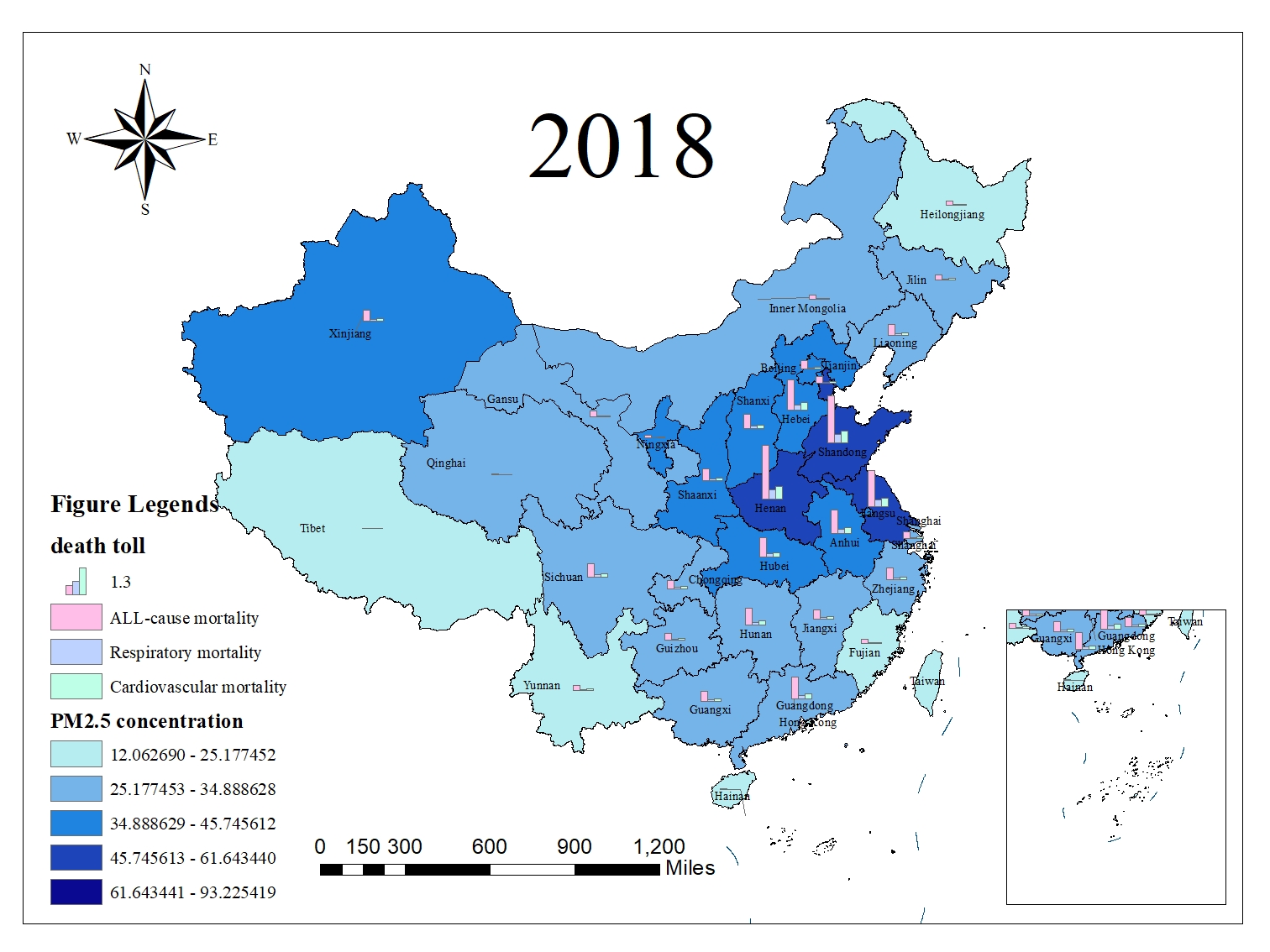

Supplement: Supplementary file 2 [file Data_Sheet_2.zip › PM2.5/PM2.5 15μgm3/2018.jpg]

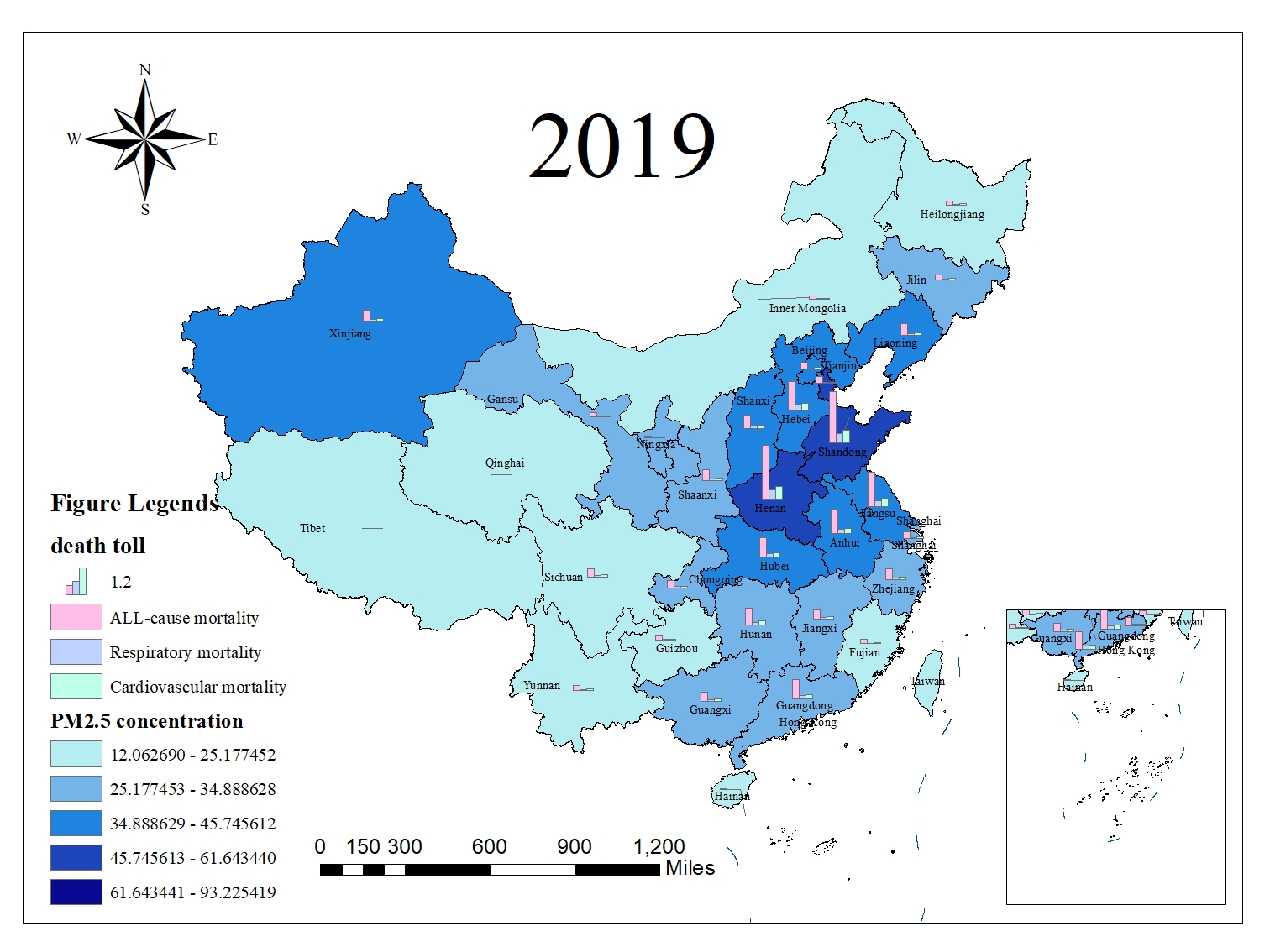

Supplement: Supplementary file 2 [file Data_Sheet_2.zip › PM2.5/PM2.5 15μgm3/2019.jpg]

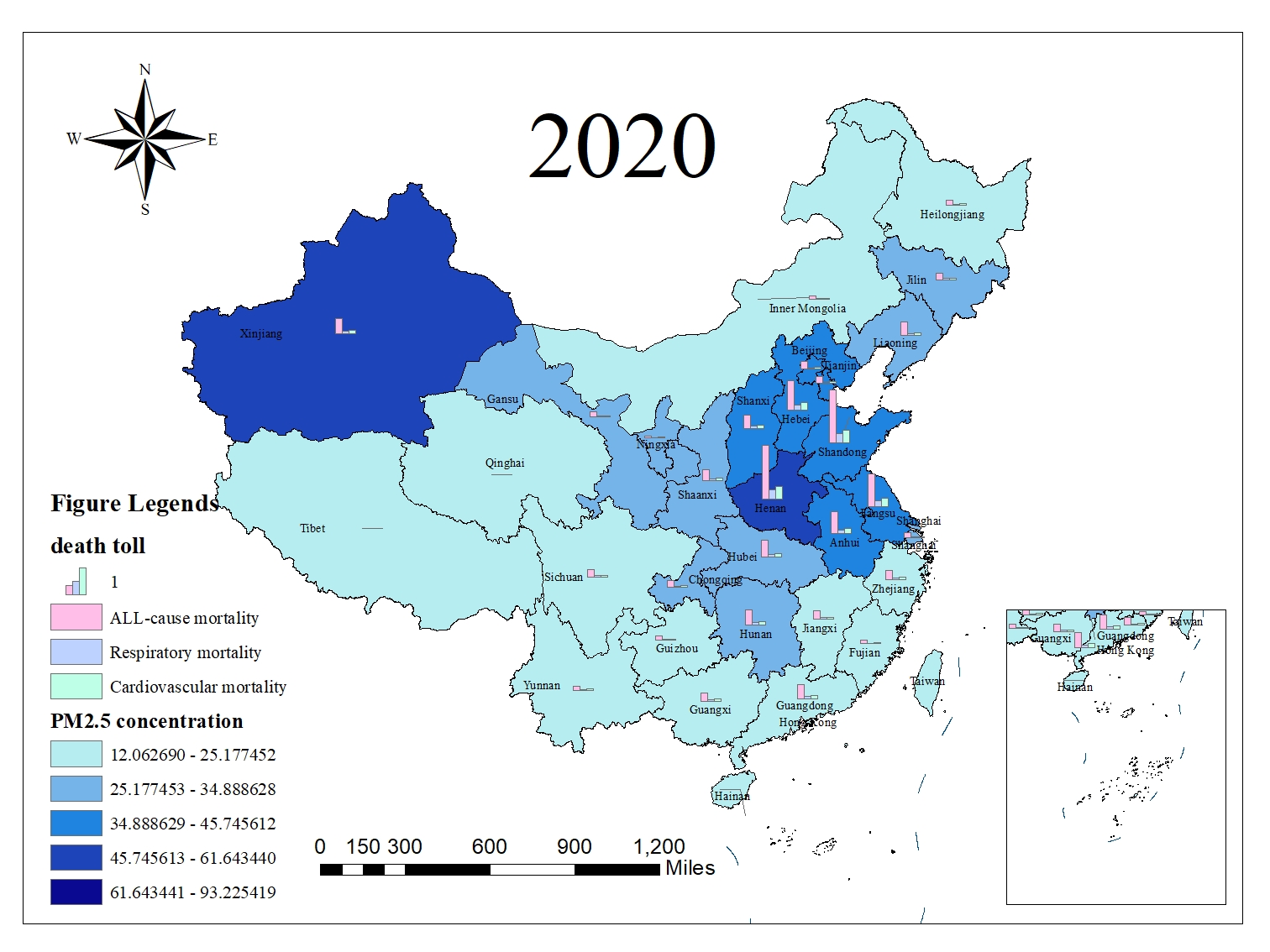

Supplement: Supplementary file 2 [file Data_Sheet_2.zip › PM2.5/PM2.5 15μgm3/2020.jpg]

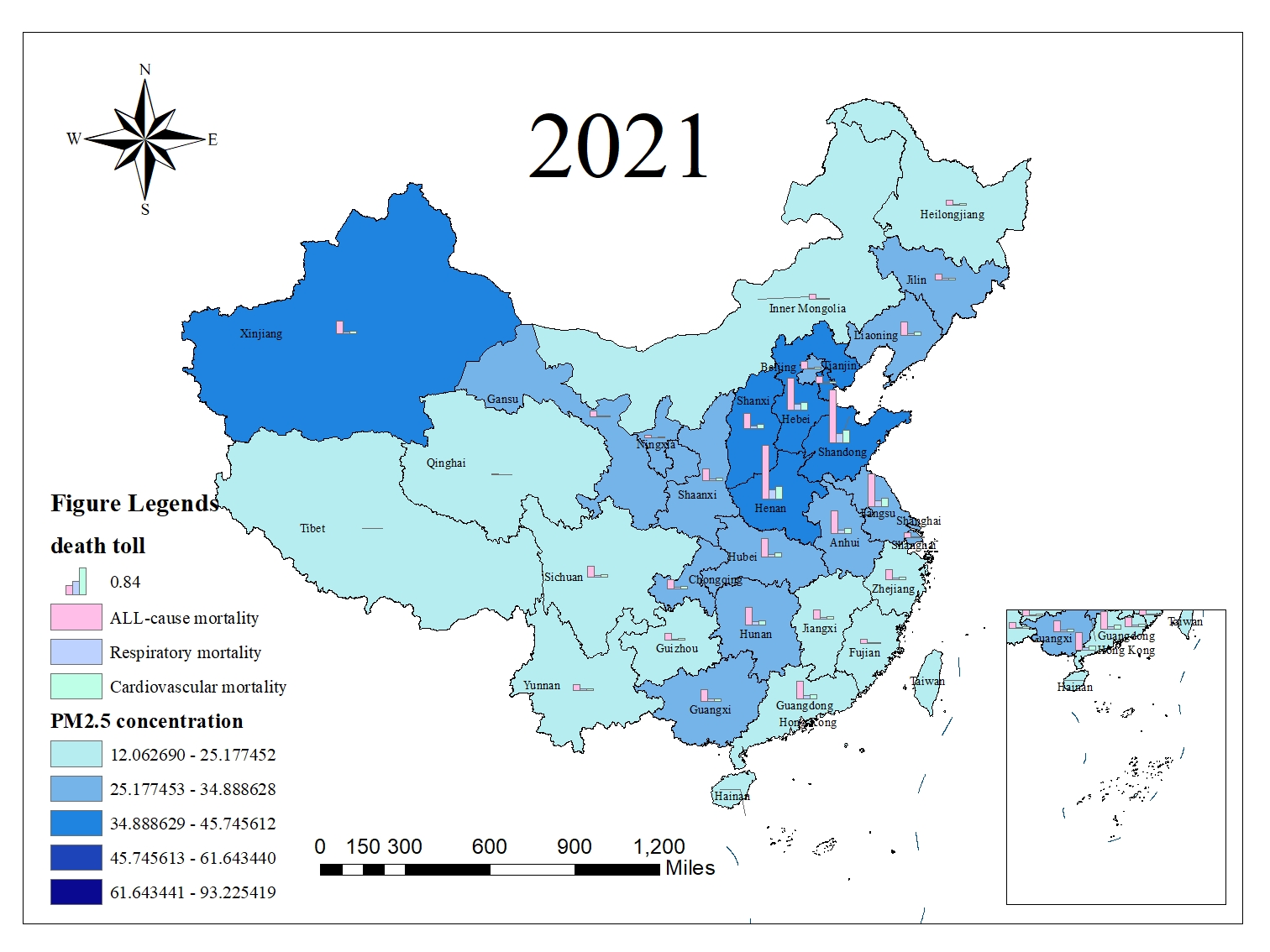

Supplement: Supplementary file 2 [file Data_Sheet_2.zip › PM2.5/PM2.5 15μgm3/2021.jpg]

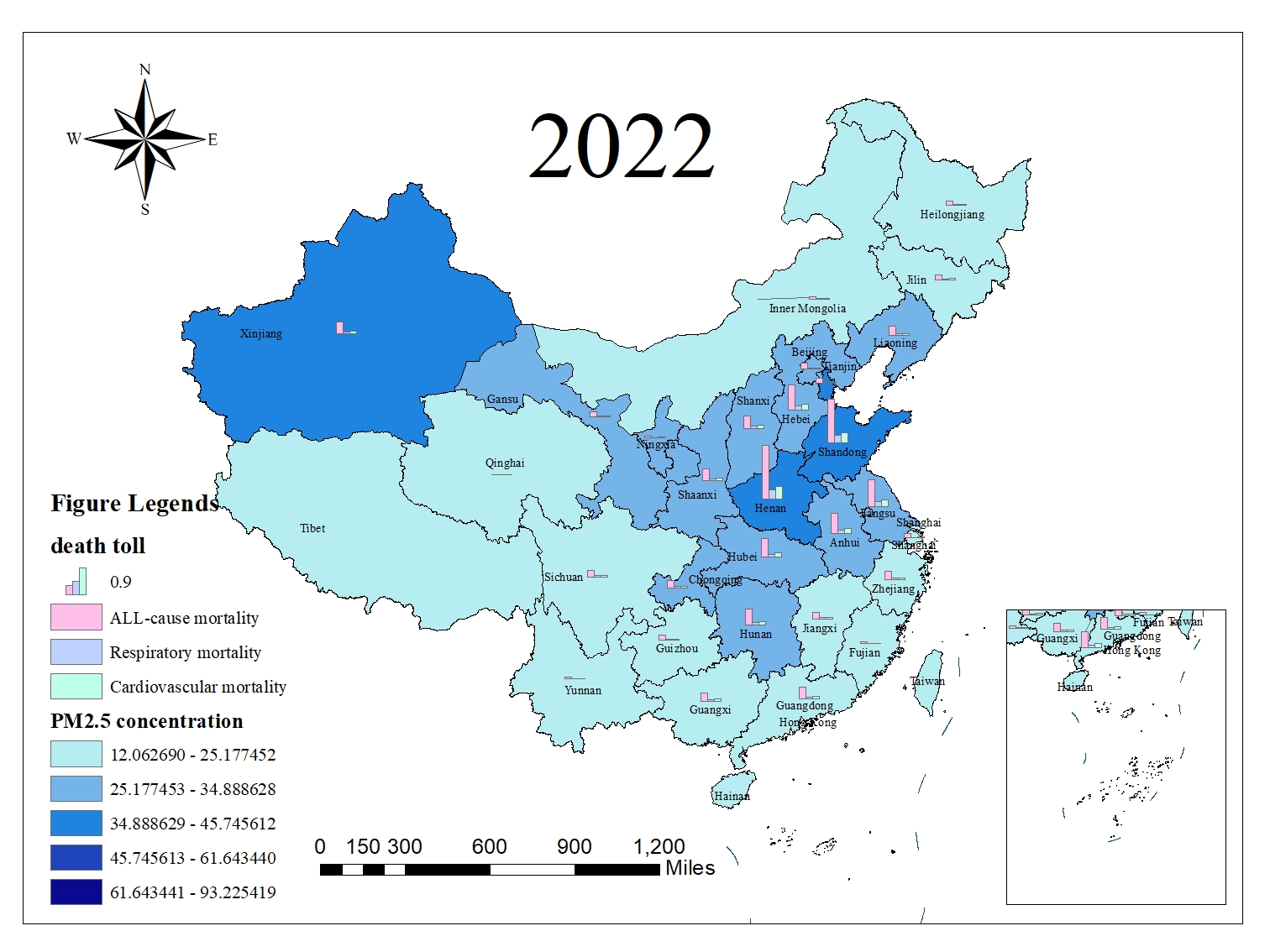

Supplement: Supplementary file 2 [file Data_Sheet_2.zip › PM2.5/PM2.5 15μgm3/2022.jpg]

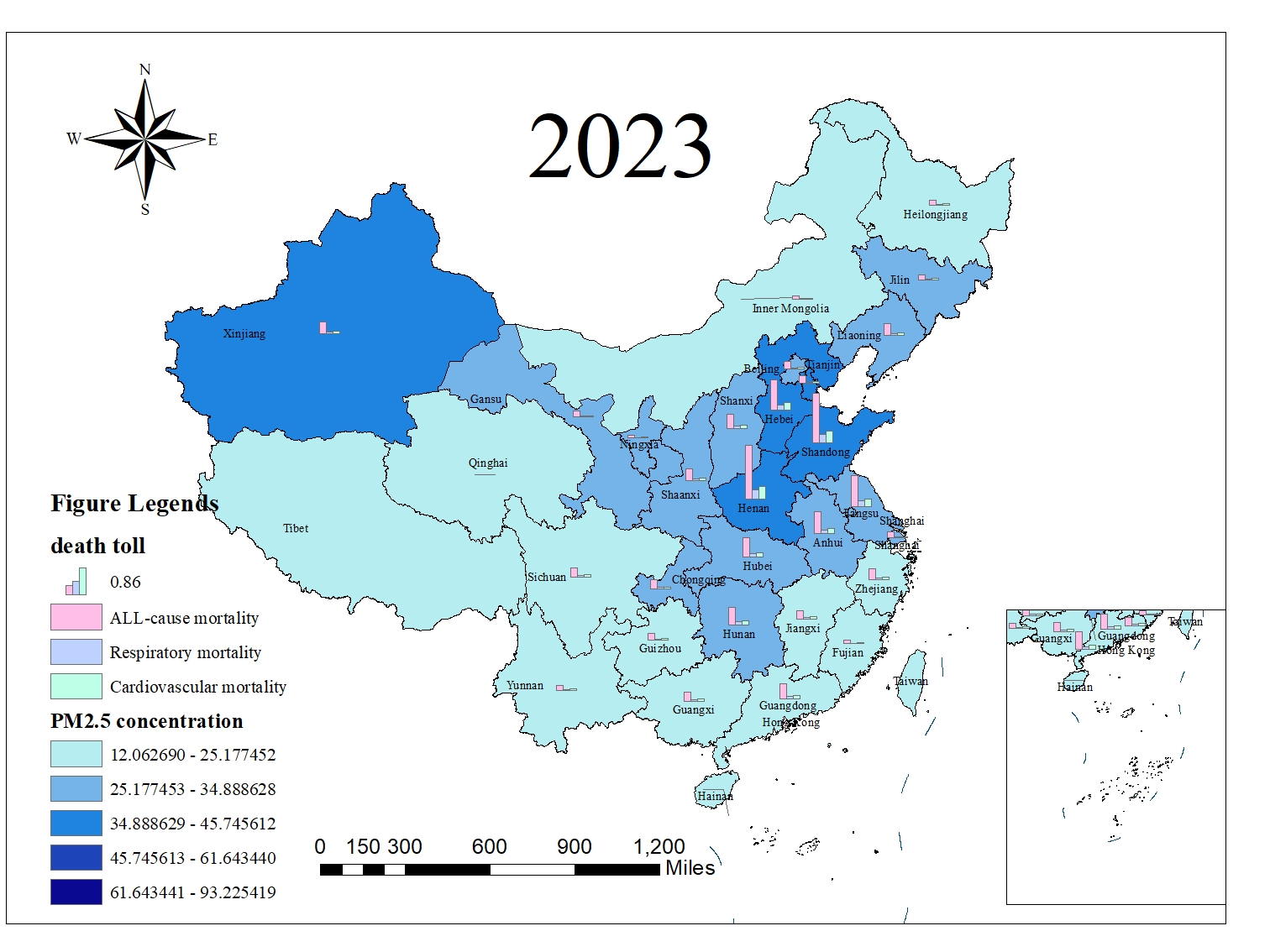

Supplement: Supplementary file 2 [file Data_Sheet_2.zip › PM2.5/PM2.5 15μgm3/2023.jpg]

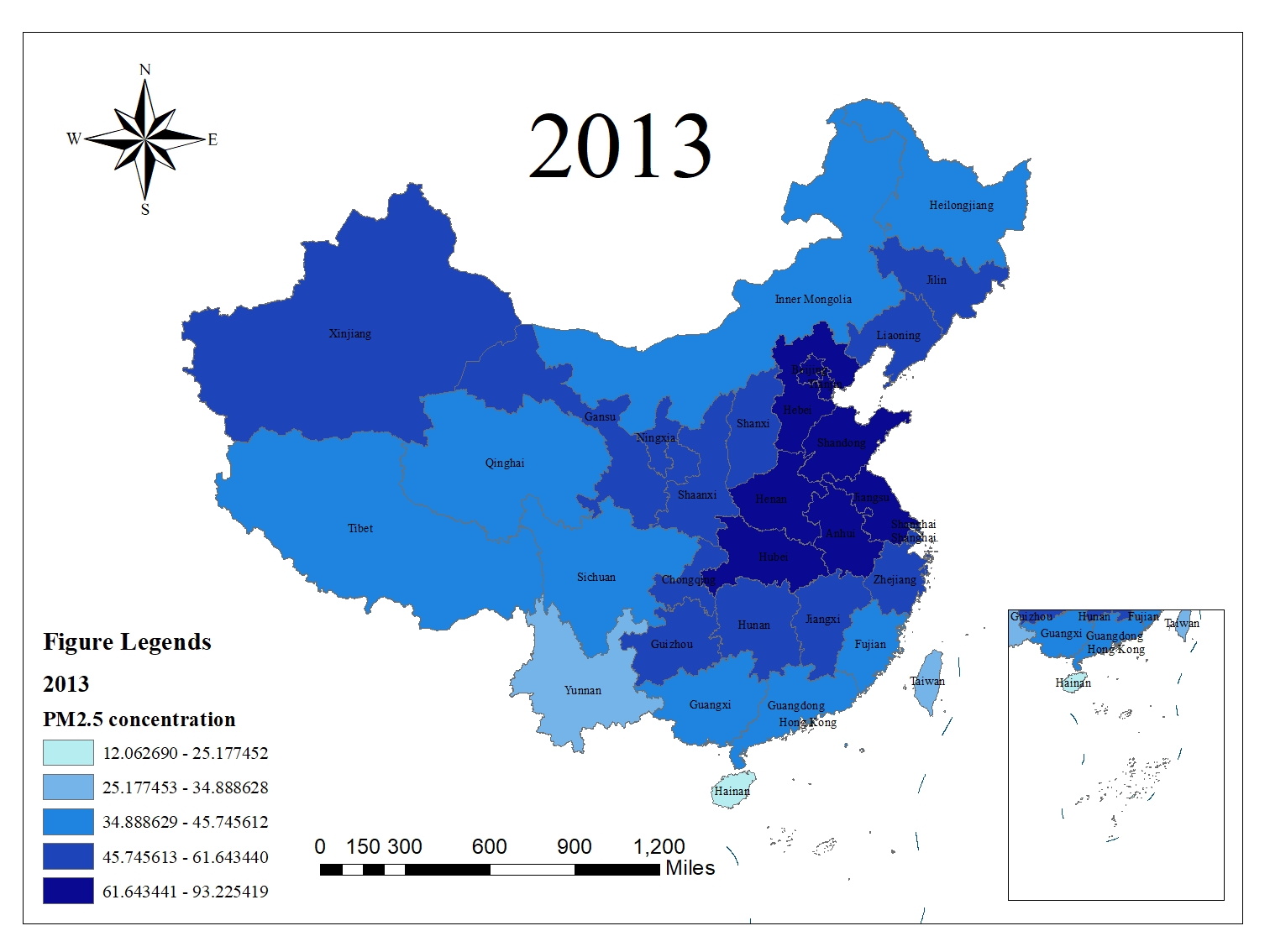

Supplement: Supplementary file 2 [file Data_Sheet_2.zip › PM2.5/PM2.5 concentration/2013.jpg]

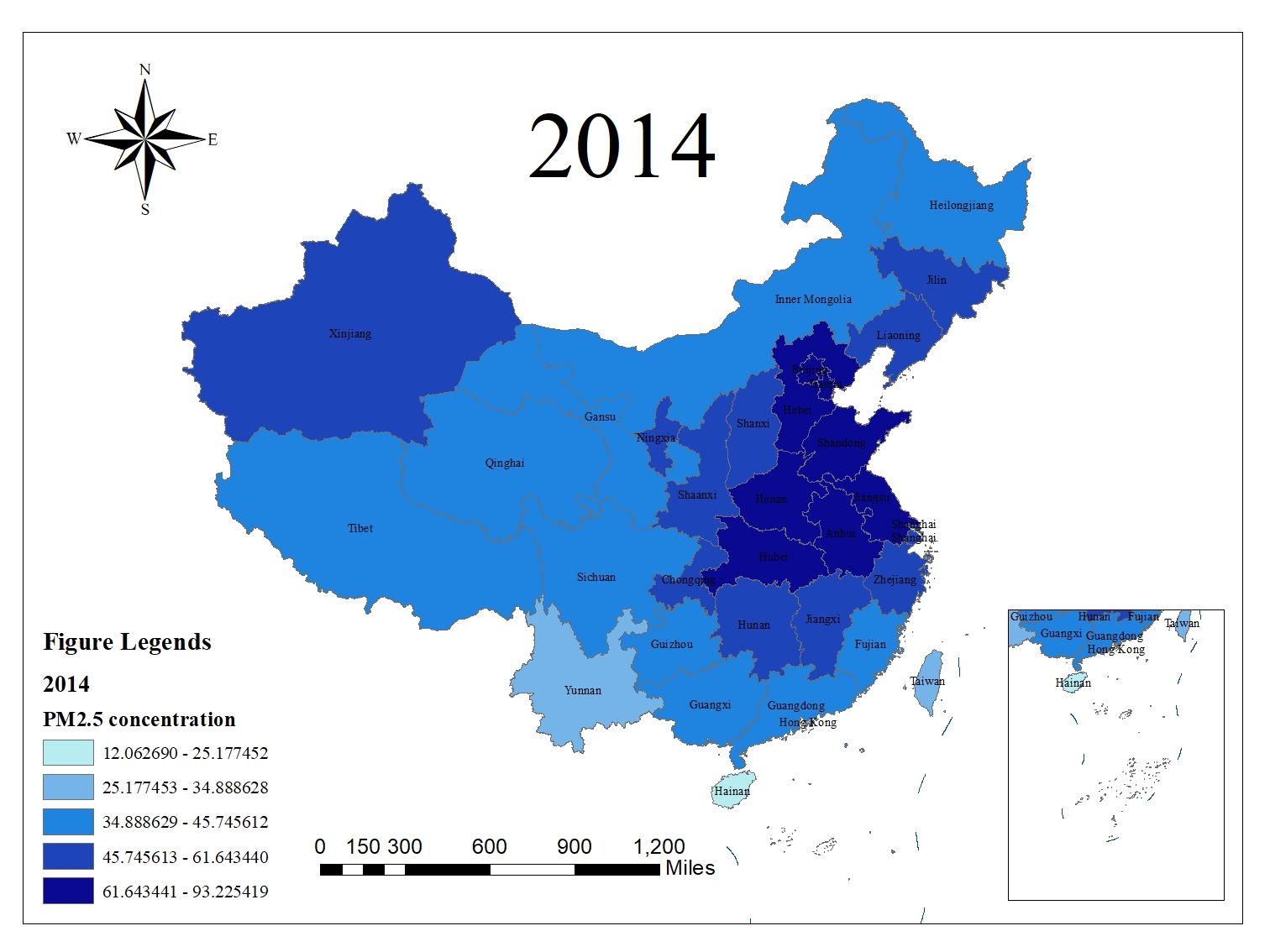

Supplement: Supplementary file 2 [file Data_Sheet_2.zip › PM2.5/PM2.5 concentration/2014.jpg]

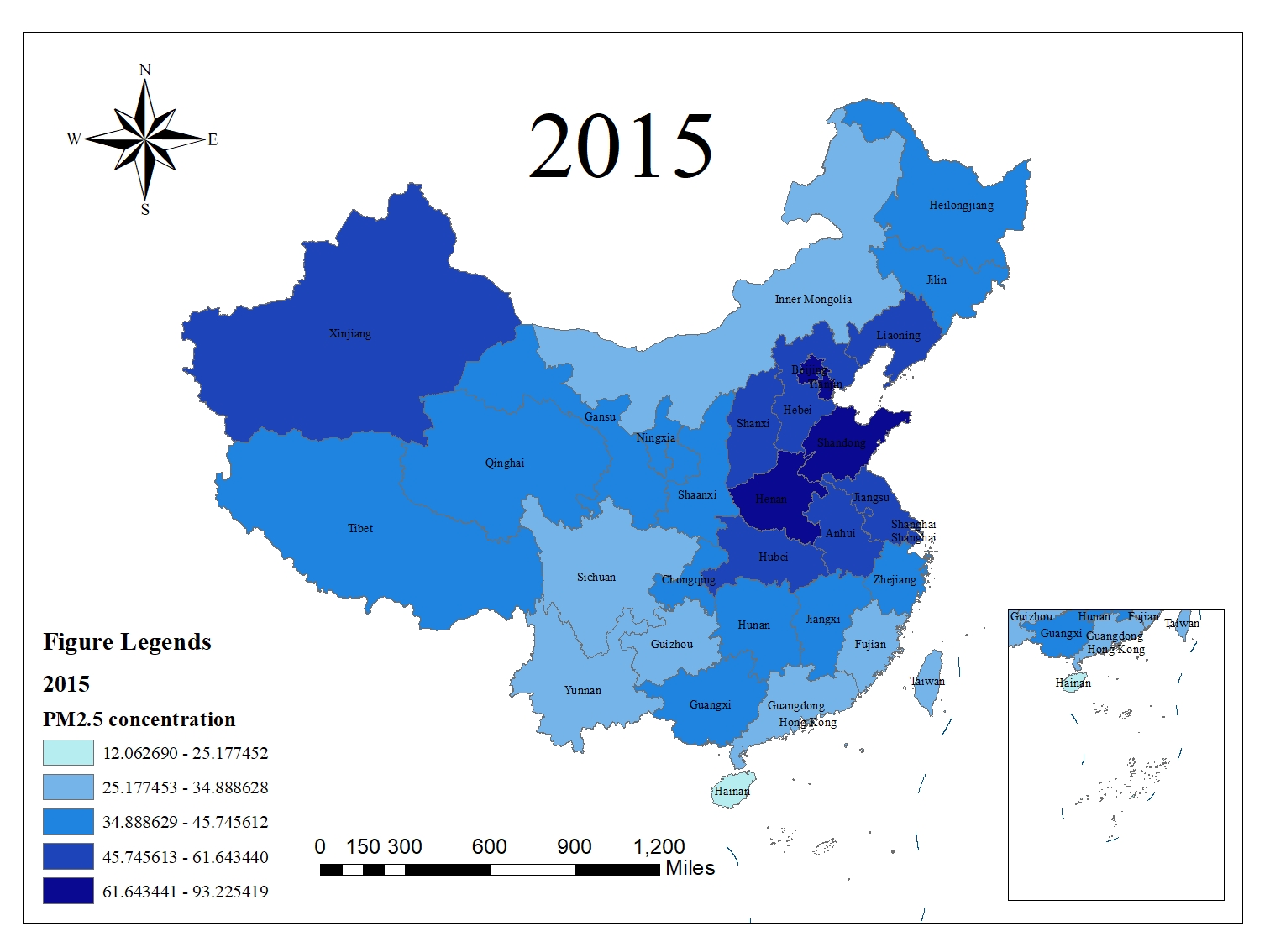

Supplement: Supplementary file 2 [file Data_Sheet_2.zip › PM2.5/PM2.5 concentration/2015.jpg]

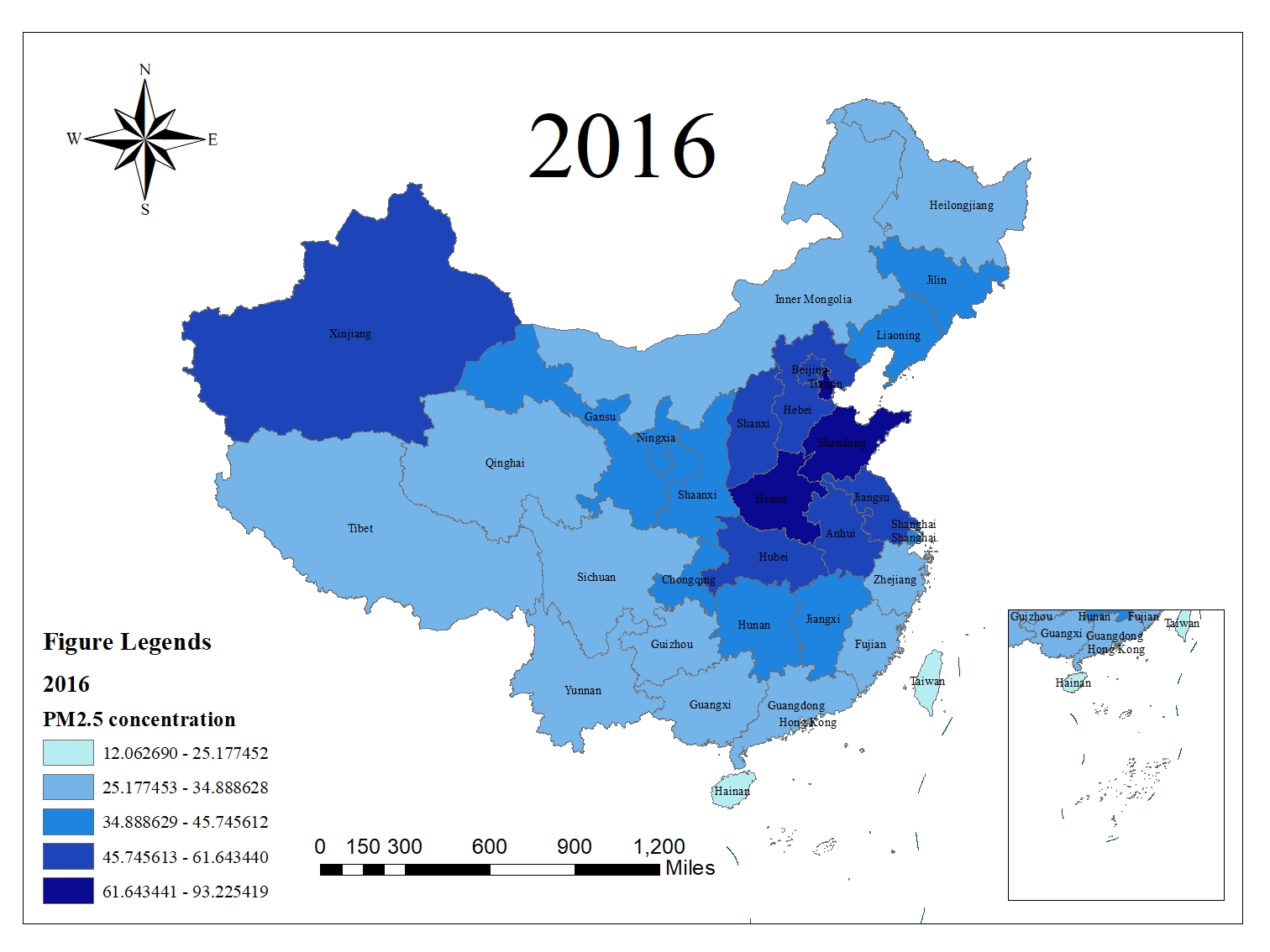

Supplement: Supplementary file 2 [file Data_Sheet_2.zip › PM2.5/PM2.5 concentration/2016.jpg]

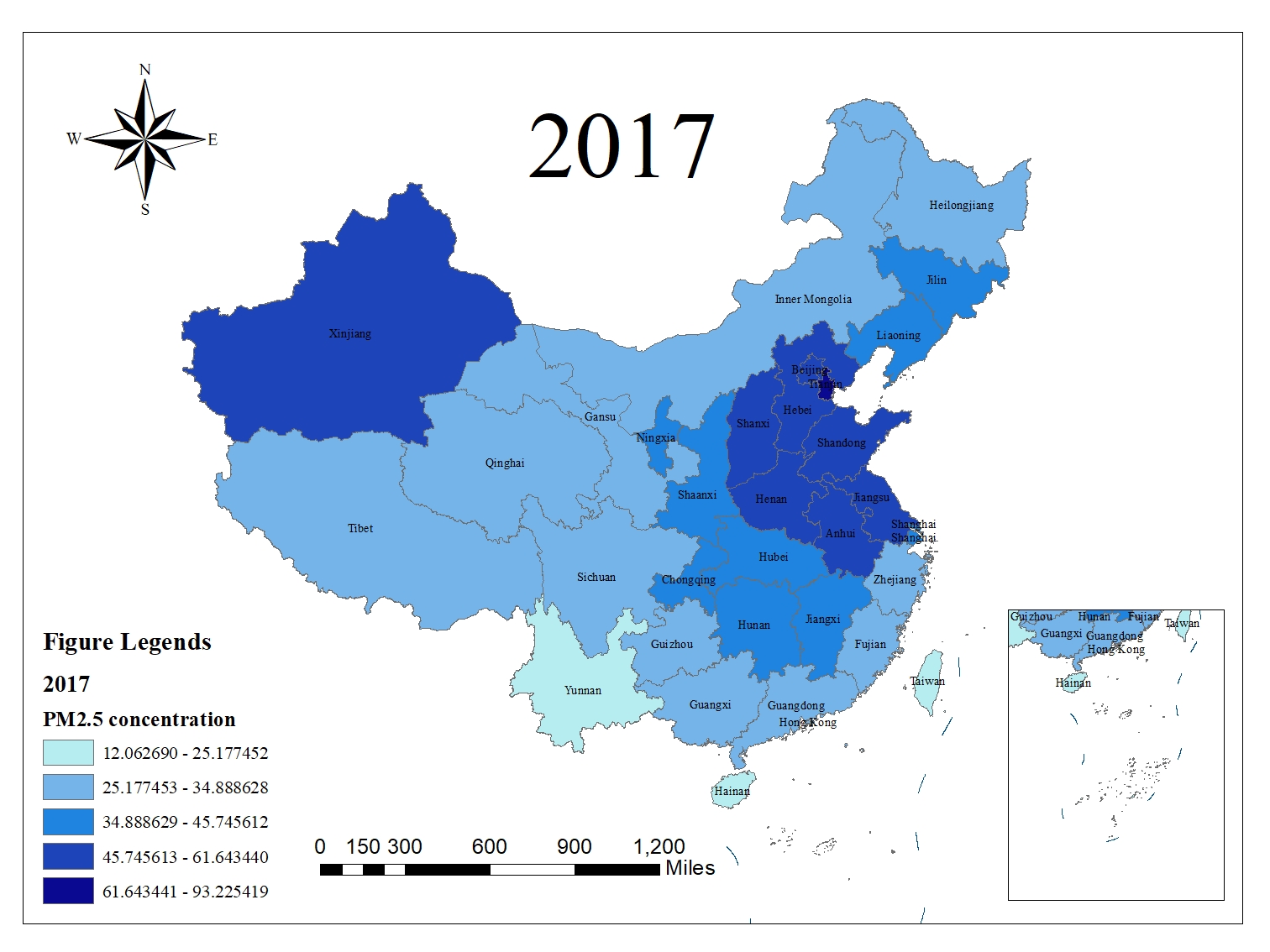

Supplement: Supplementary file 2 [file Data_Sheet_2.zip › PM2.5/PM2.5 concentration/2017.jpg]

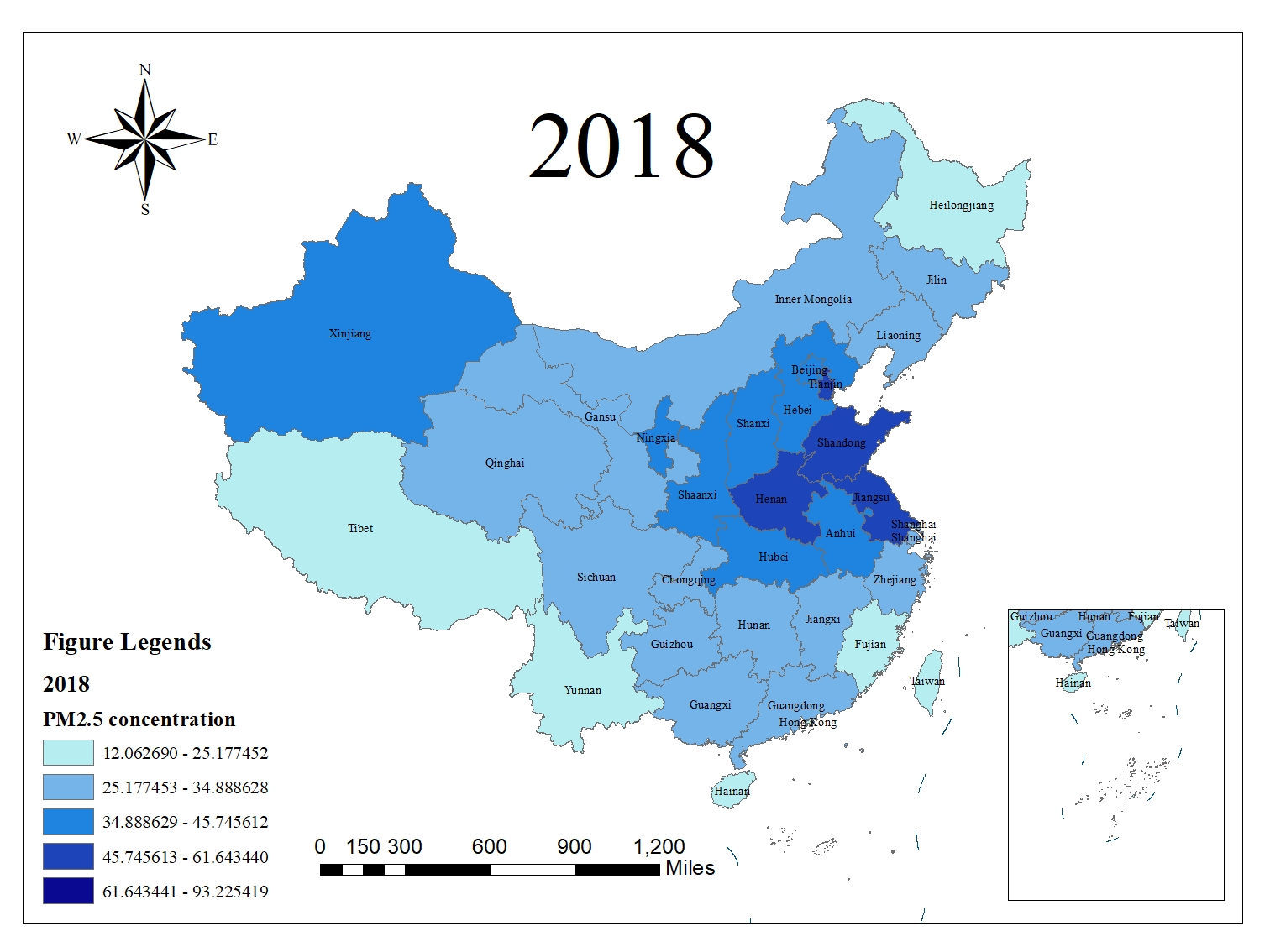

Supplement: Supplementary file 2 [file Data_Sheet_2.zip › PM2.5/PM2.5 concentration/2018.jpg]

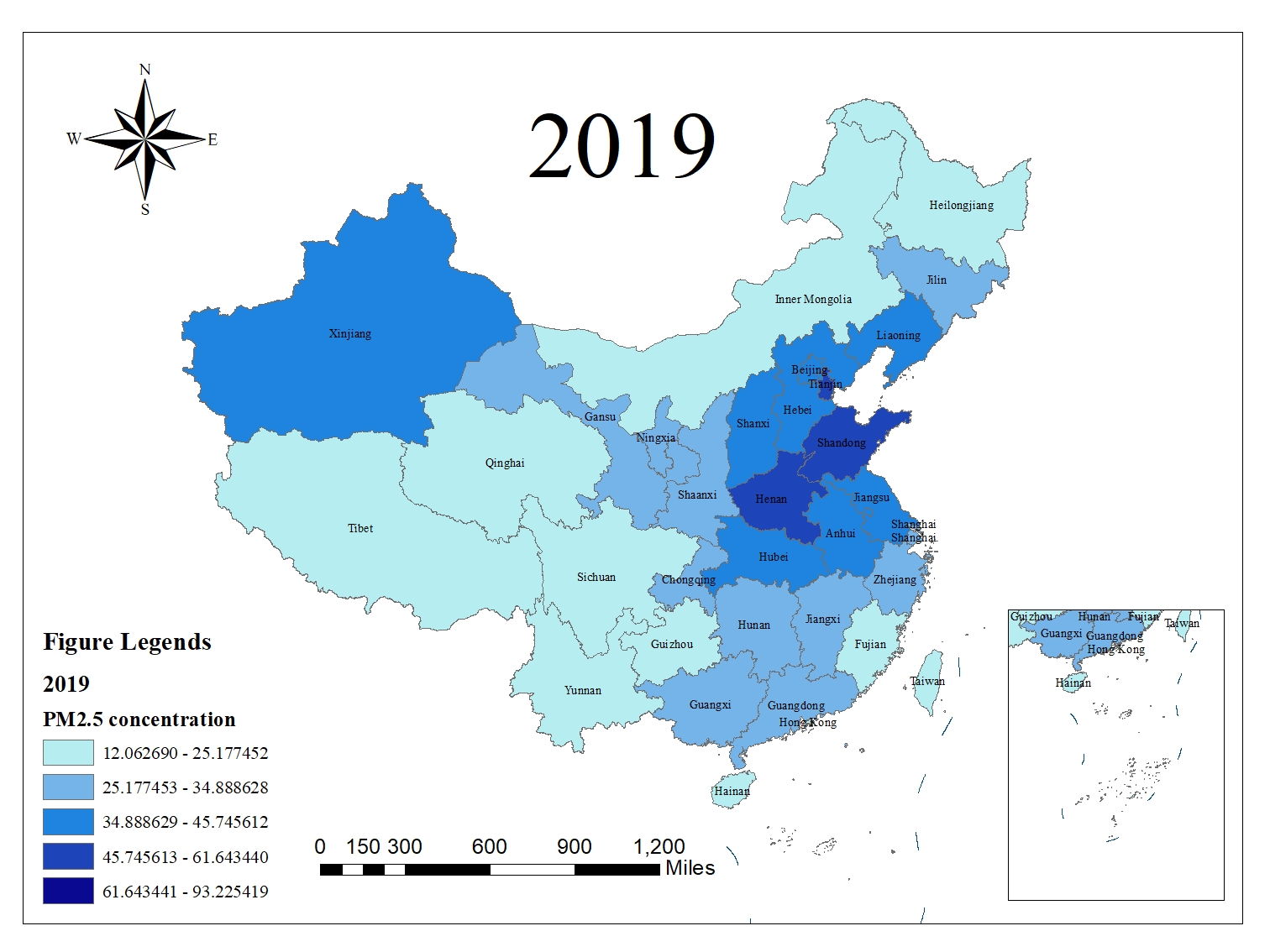

Supplement: Supplementary file 2 [file Data_Sheet_2.zip › PM2.5/PM2.5 concentration/2019.jpg]

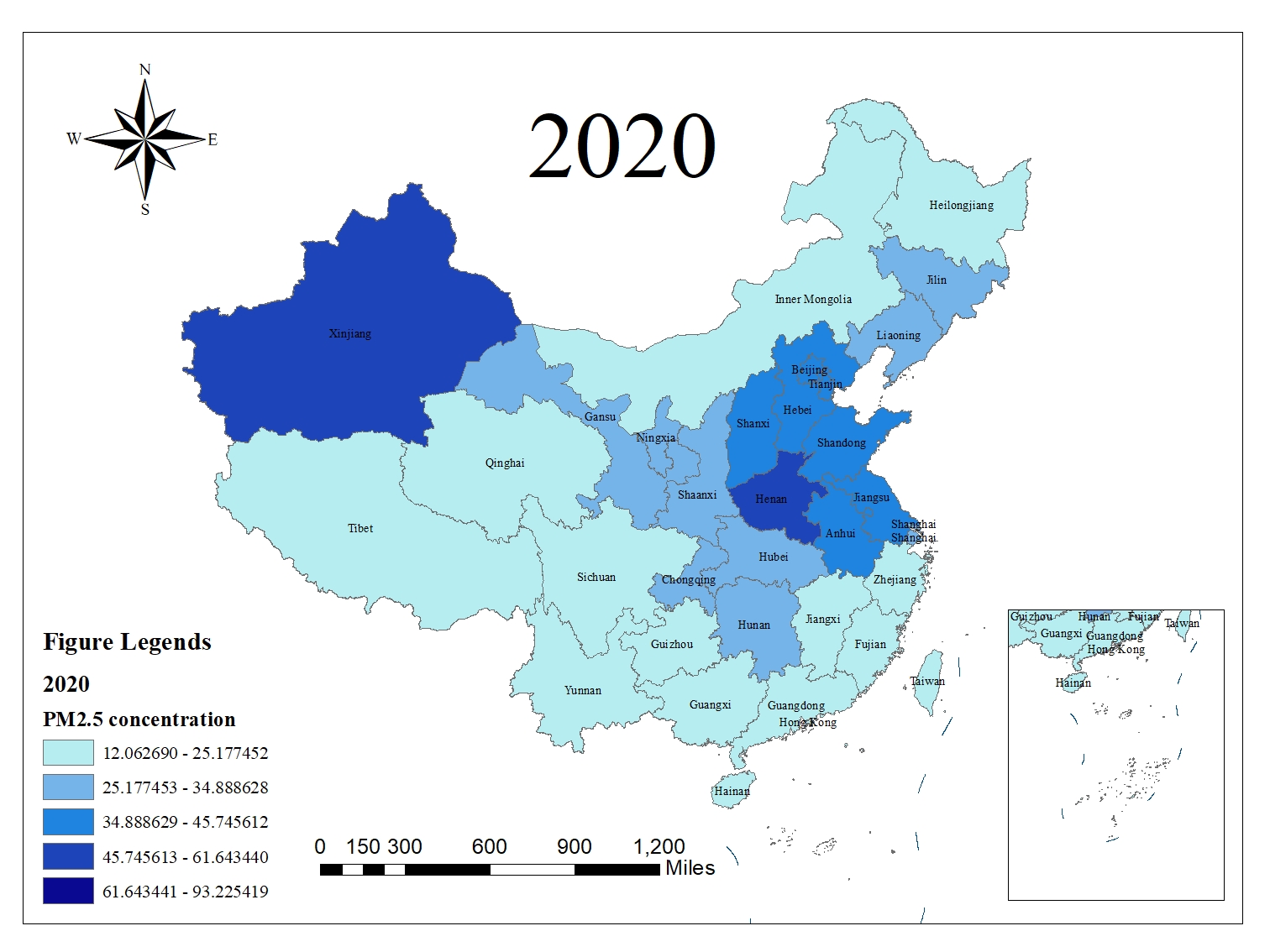

Supplement: Supplementary file 2 [file Data_Sheet_2.zip › PM2.5/PM2.5 concentration/2020.jpg]

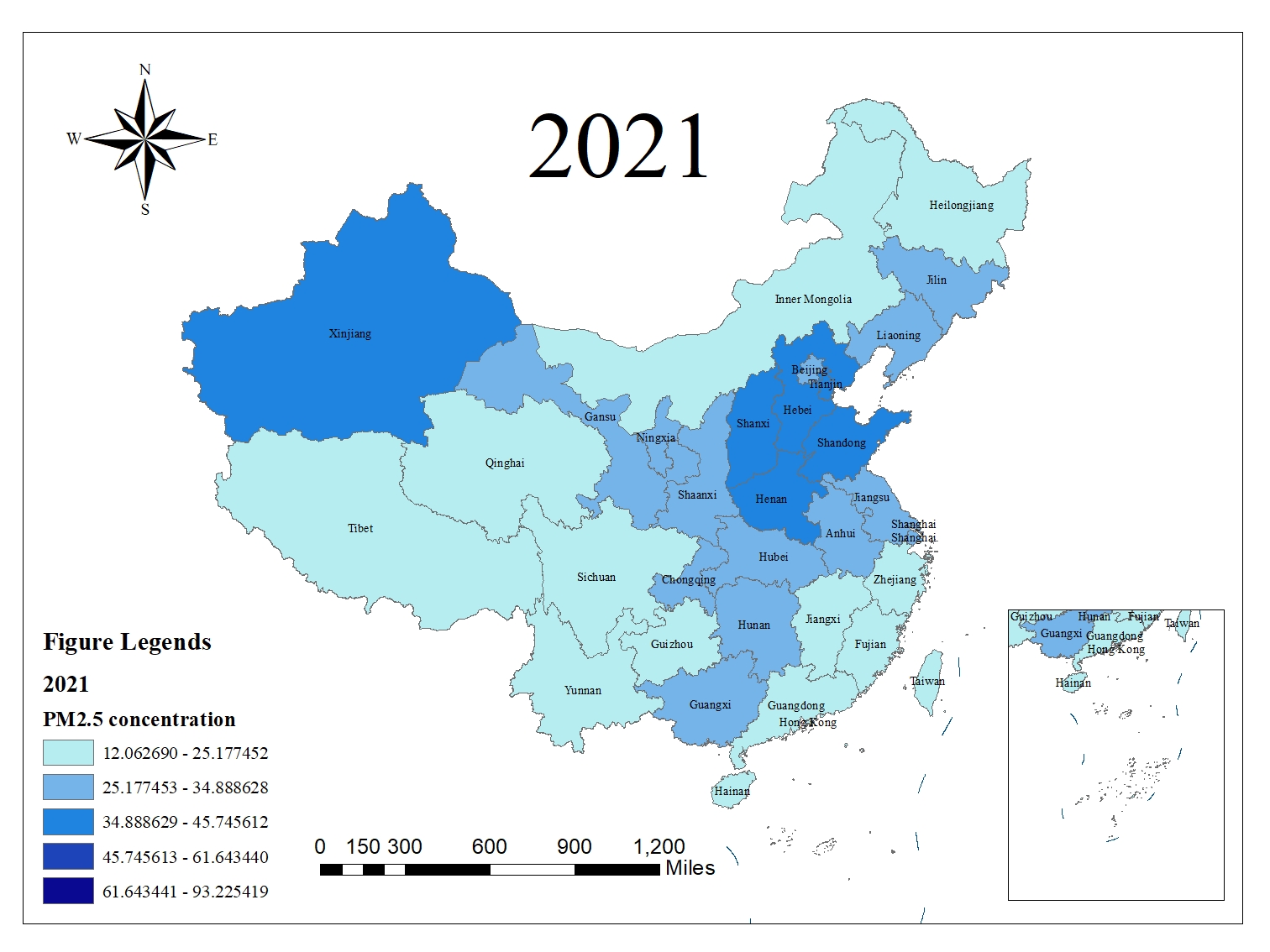

Supplement: Supplementary file 2 [file Data_Sheet_2.zip › PM2.5/PM2.5 concentration/2021.jpg]

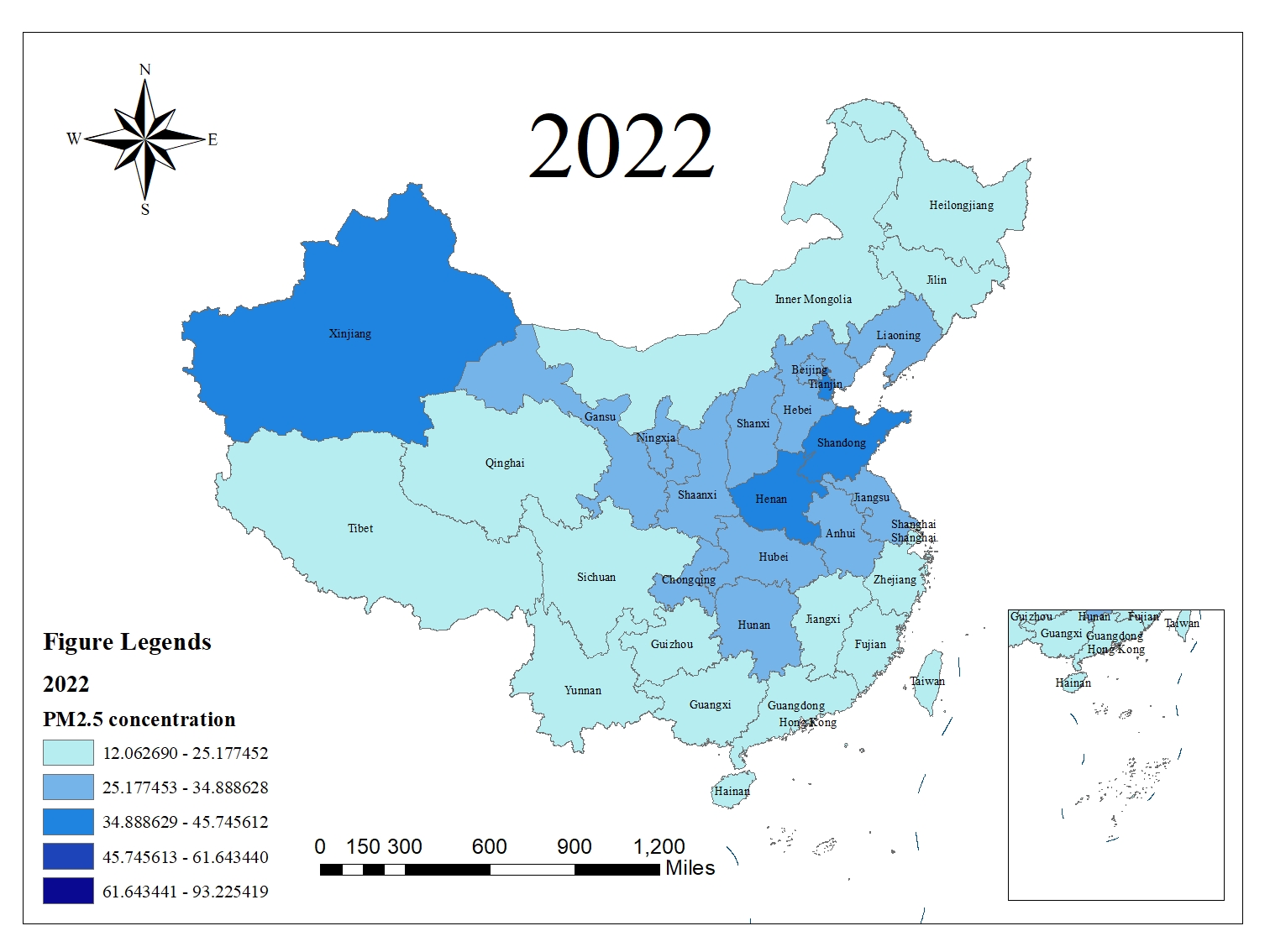

Supplement: Supplementary file 2 [file Data_Sheet_2.zip › PM2.5/PM2.5 concentration/2022.jpg]

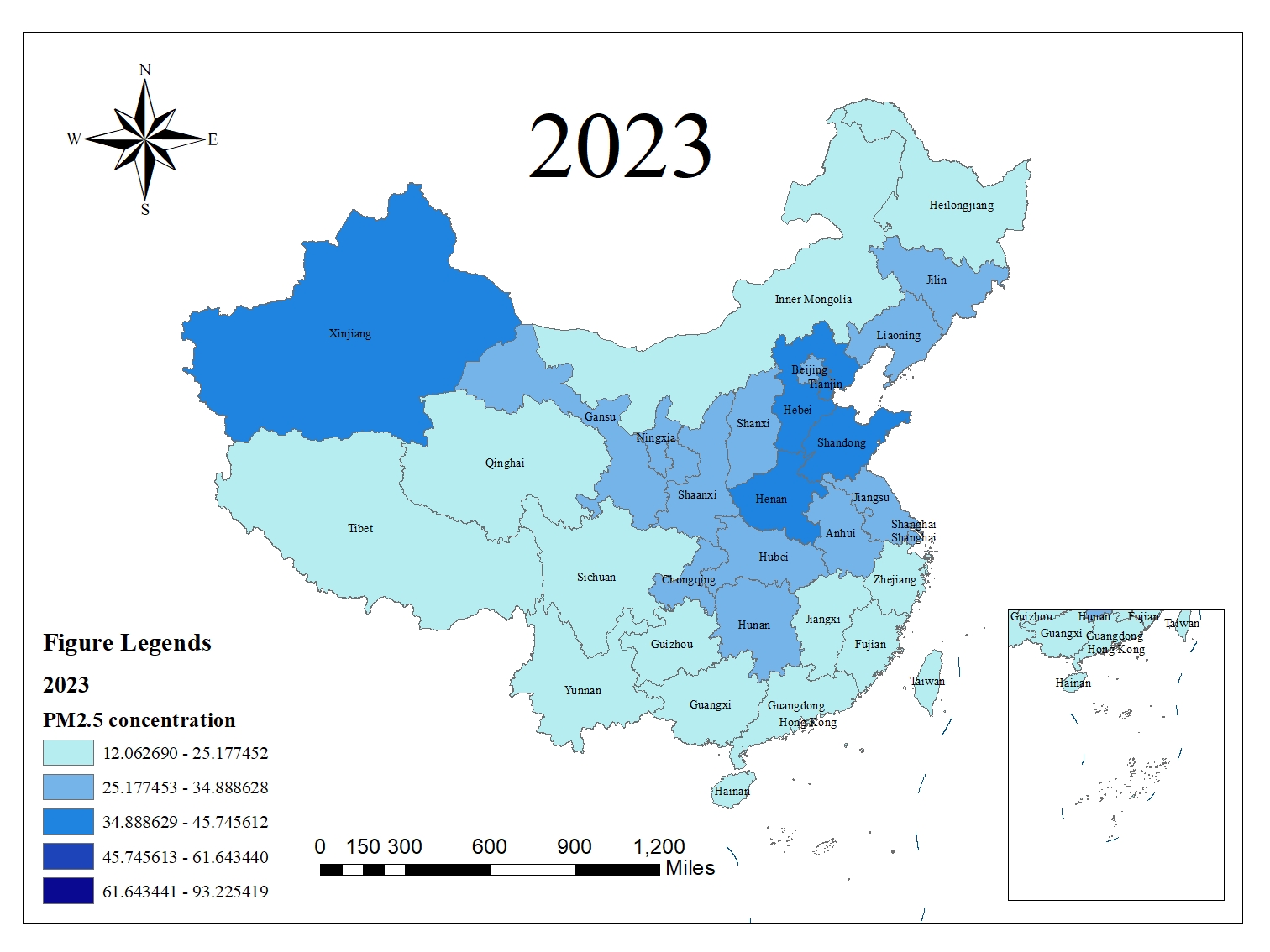

Supplement: Supplementary file 2 [file Data_Sheet_2.zip › PM2.5/PM2.5 concentration/2023.jpg]

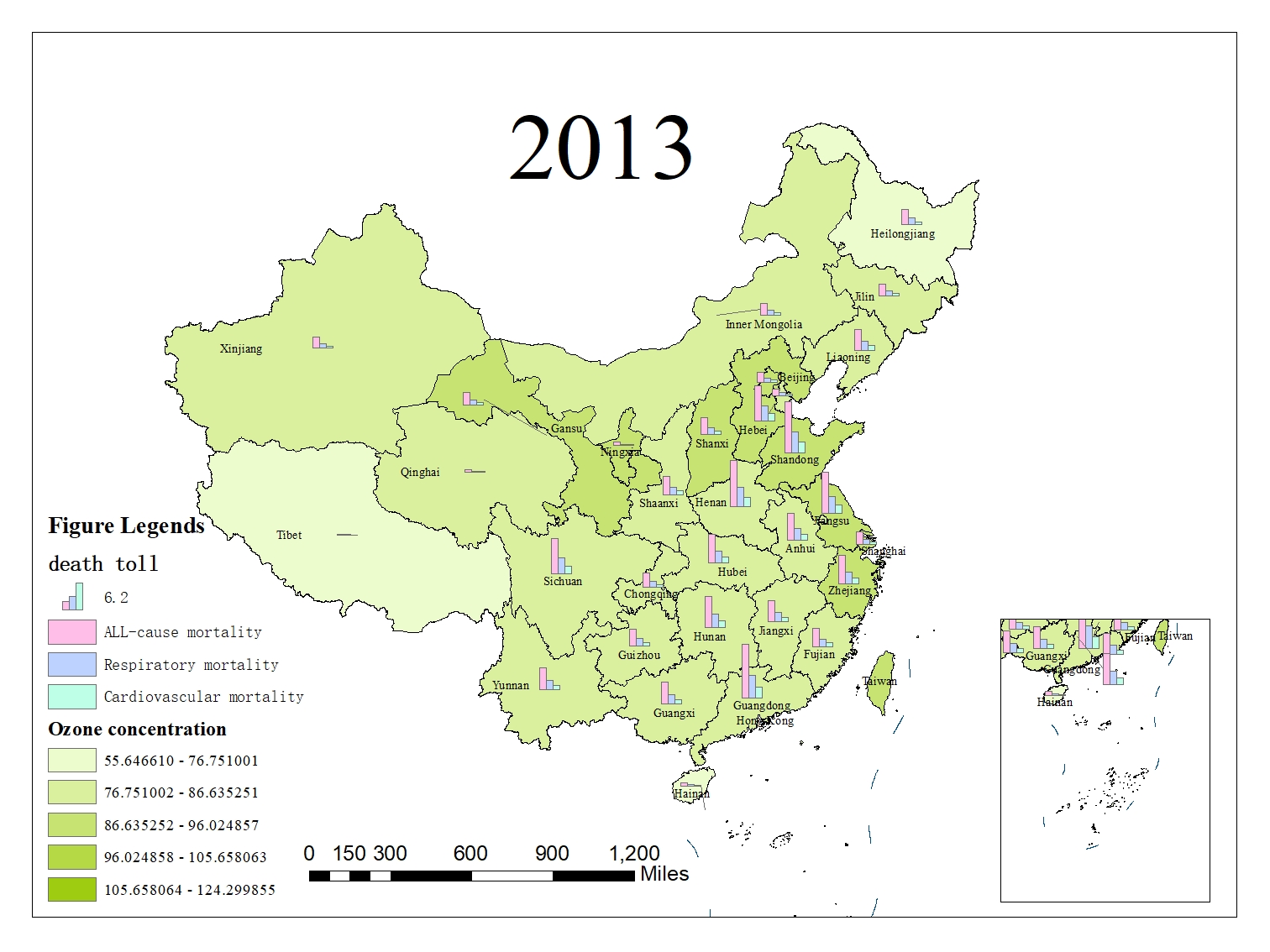

Supplement: Supplementary file 3 [file Data_Sheet_3.zip › O3/O3 0μgm3/2013.jpg]

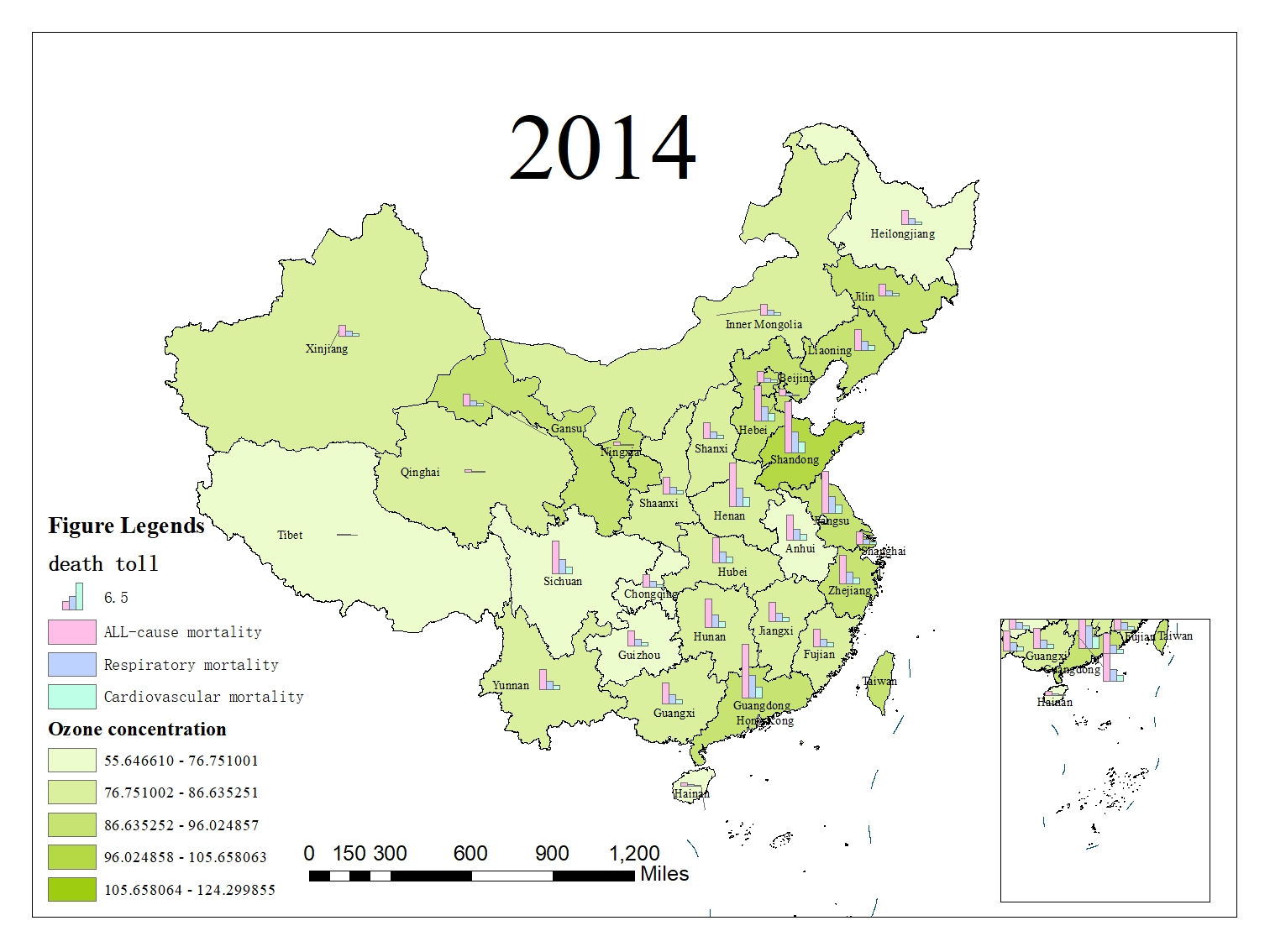

Supplement: Supplementary file 3 [file Data_Sheet_3.zip › O3/O3 0μgm3/2014.jpg]

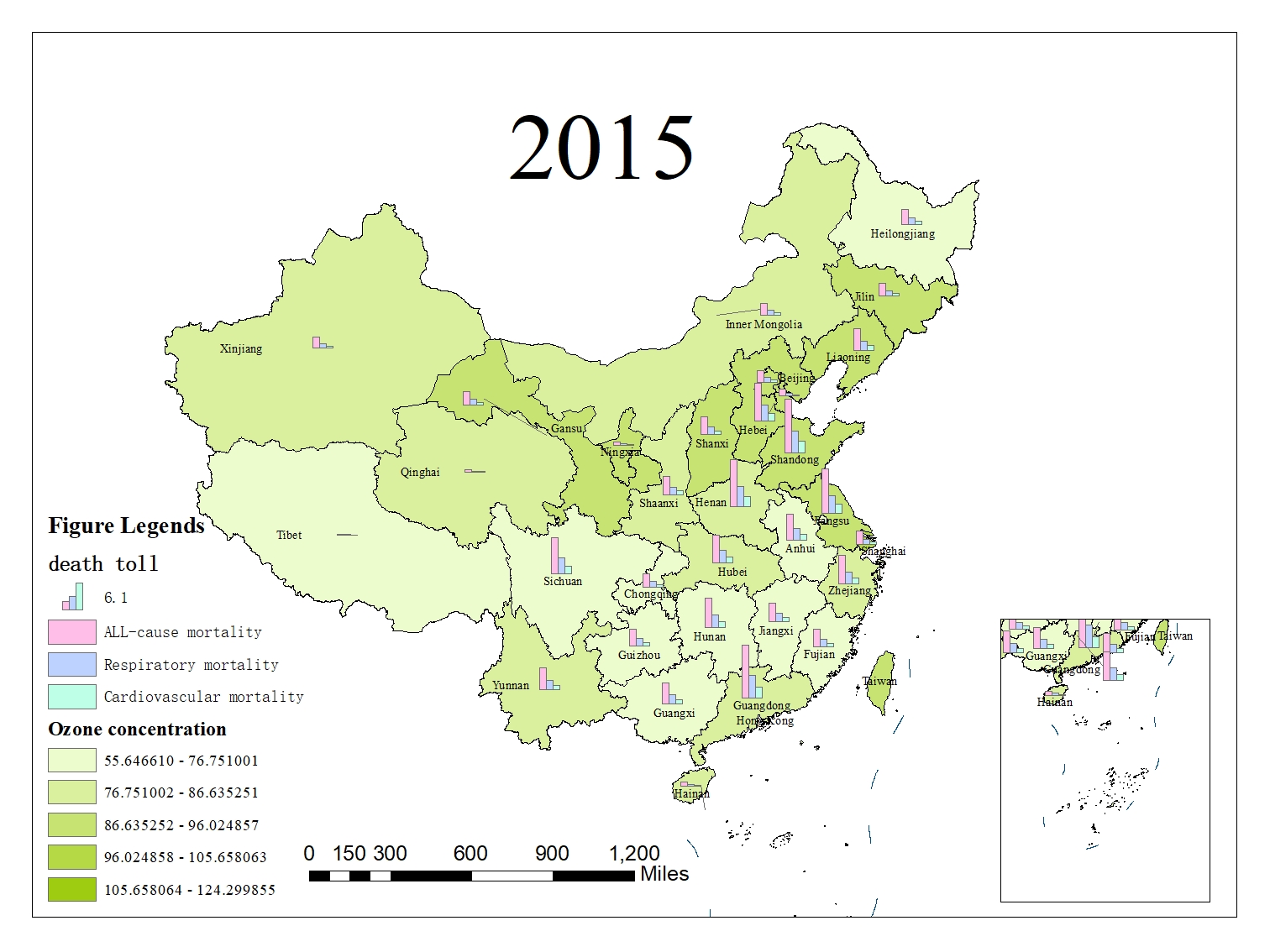

Supplement: Supplementary file 3 [file Data_Sheet_3.zip › O3/O3 0μgm3/2015.jpg]

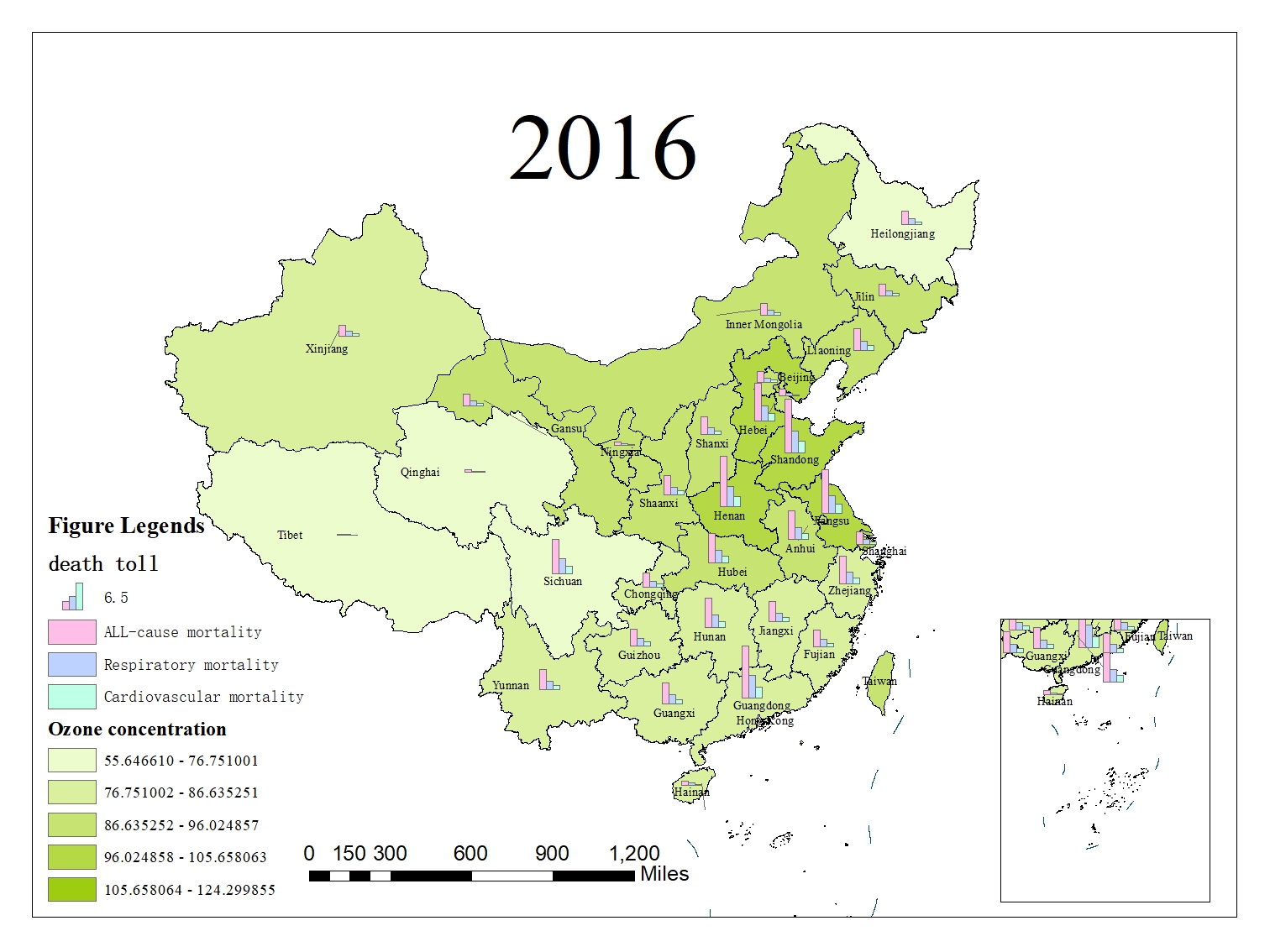

Supplement: Supplementary file 3 [file Data_Sheet_3.zip › O3/O3 0μgm3/2016.jpg]

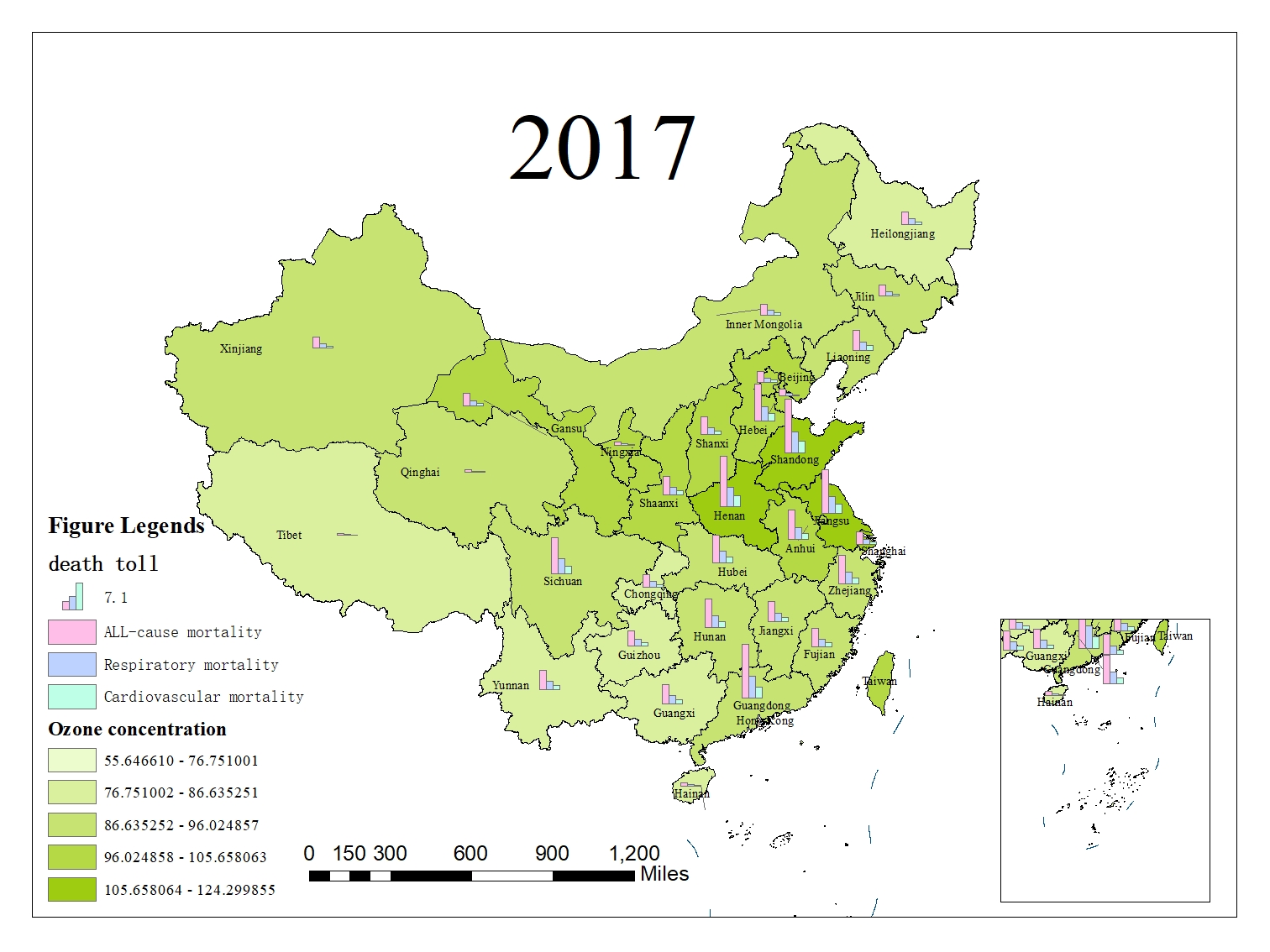

Supplement: Supplementary file 3 [file Data_Sheet_3.zip › O3/O3 0μgm3/2017.jpg]

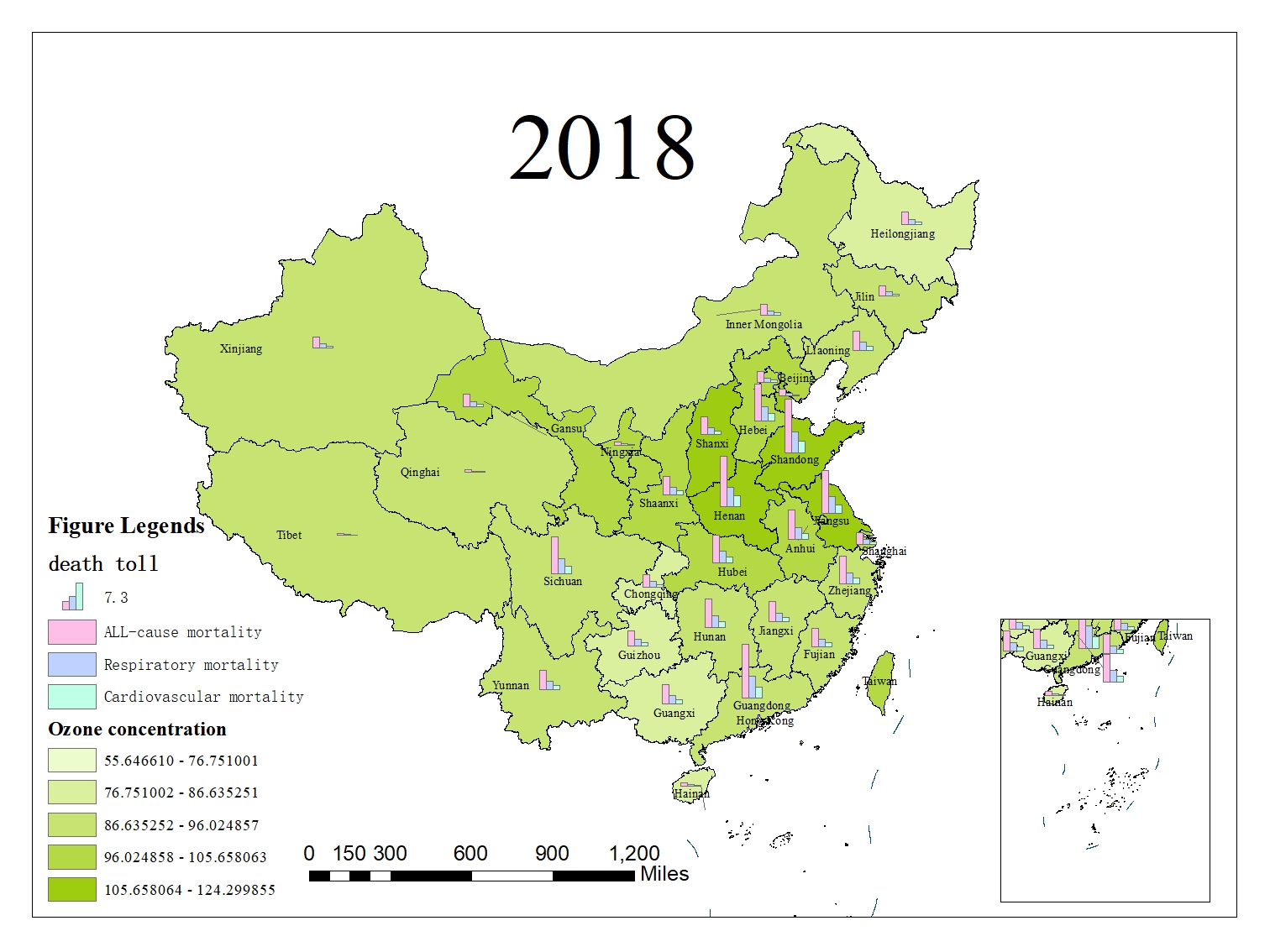

Supplement: Supplementary file 3 [file Data_Sheet_3.zip › O3/O3 0μgm3/2018.jpg]

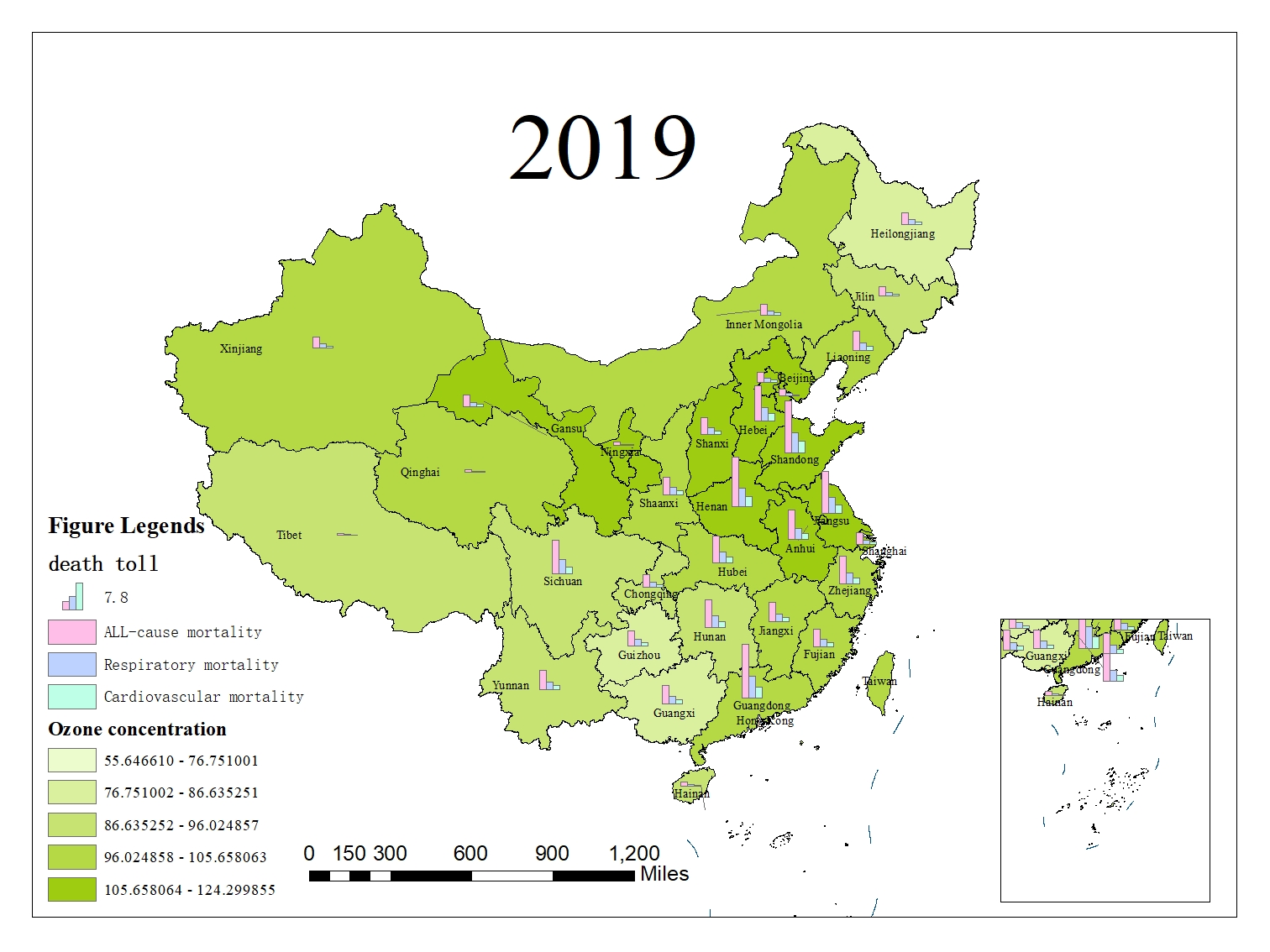

Supplement: Supplementary file 3 [file Data_Sheet_3.zip › O3/O3 0μgm3/2019.jpg]

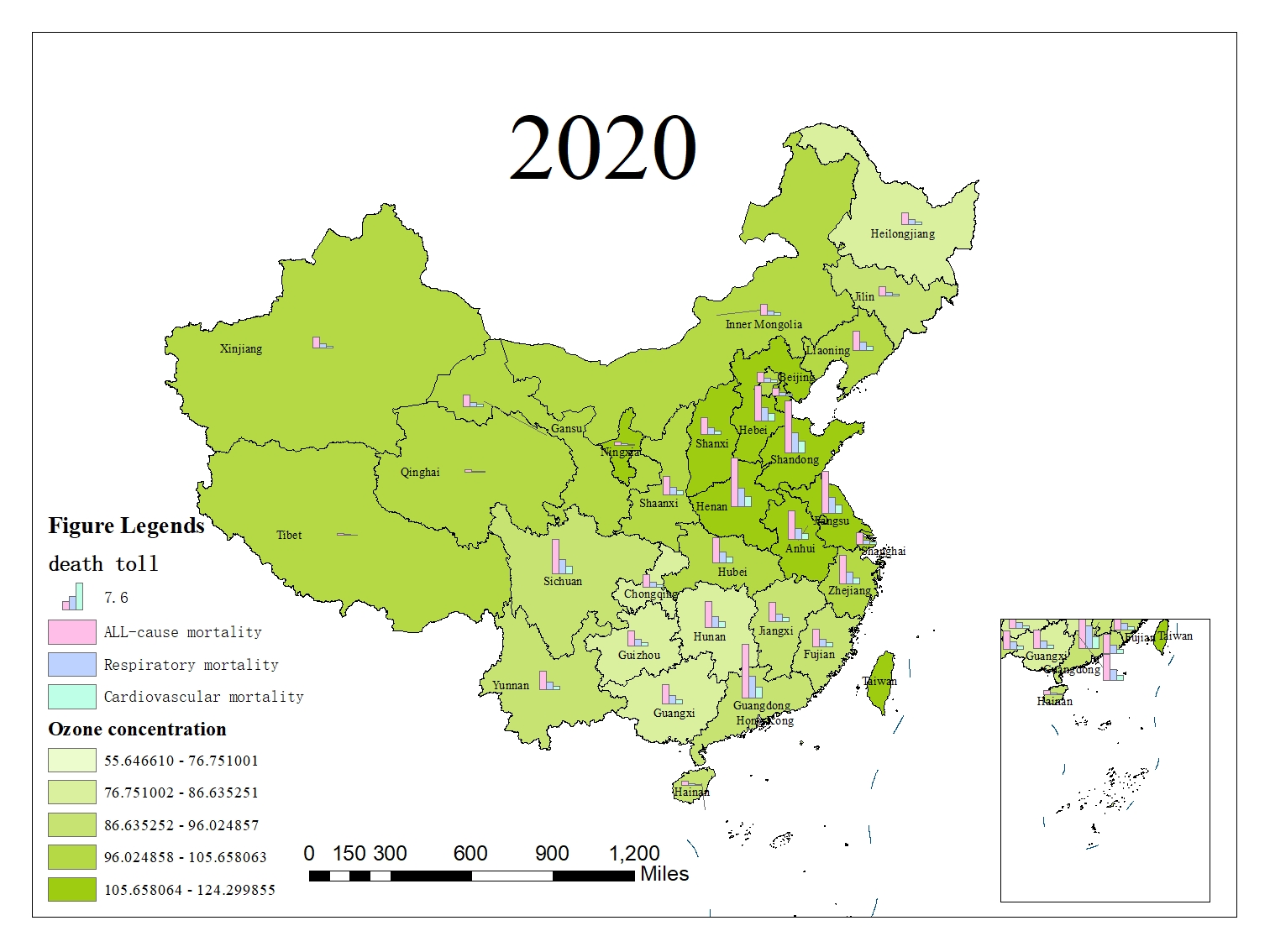

Supplement: Supplementary file 3 [file Data_Sheet_3.zip › O3/O3 0μgm3/2020.jpg]

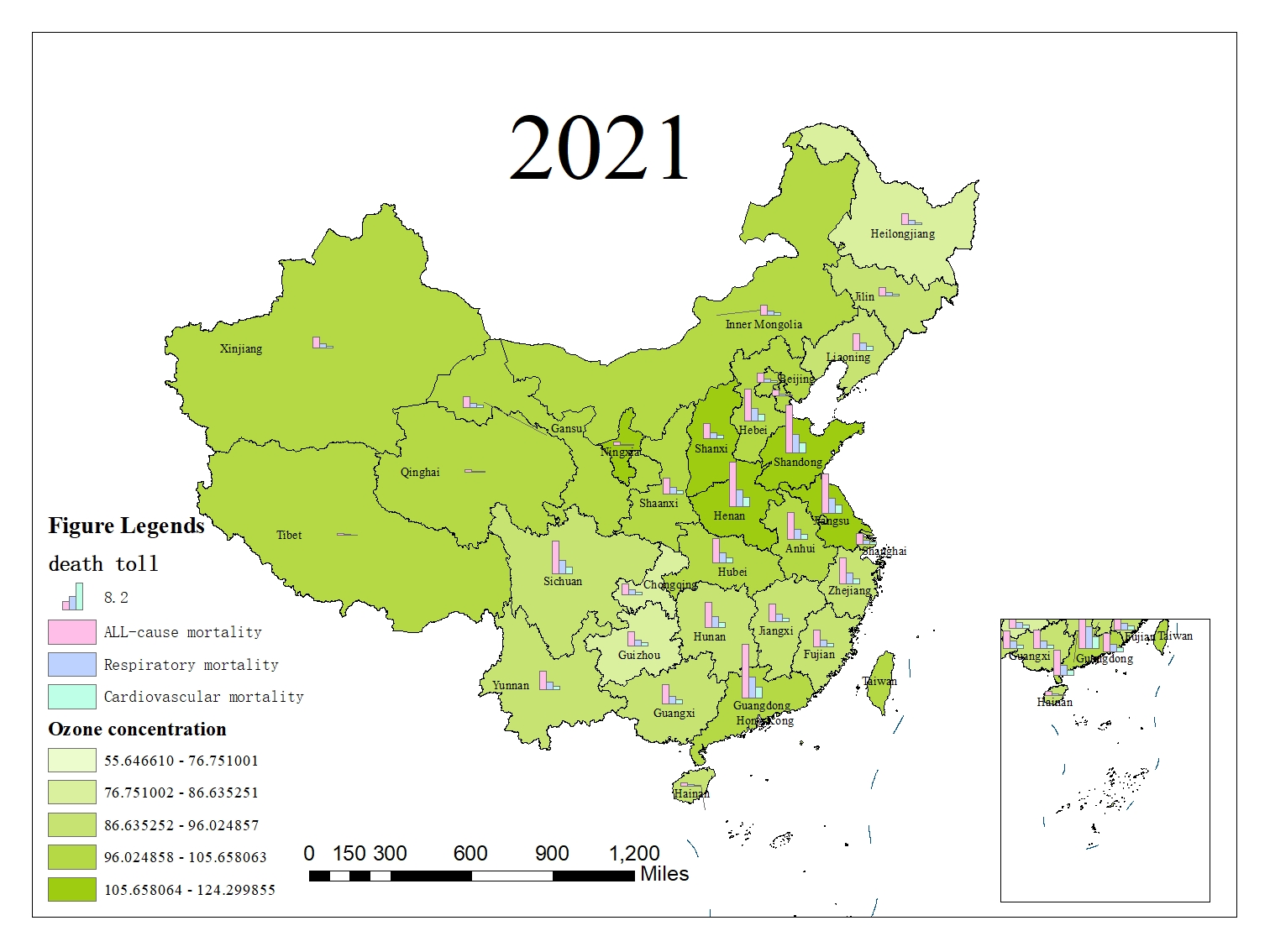

Supplement: Supplementary file 3 [file Data_Sheet_3.zip › O3/O3 0μgm3/2021.jpg]

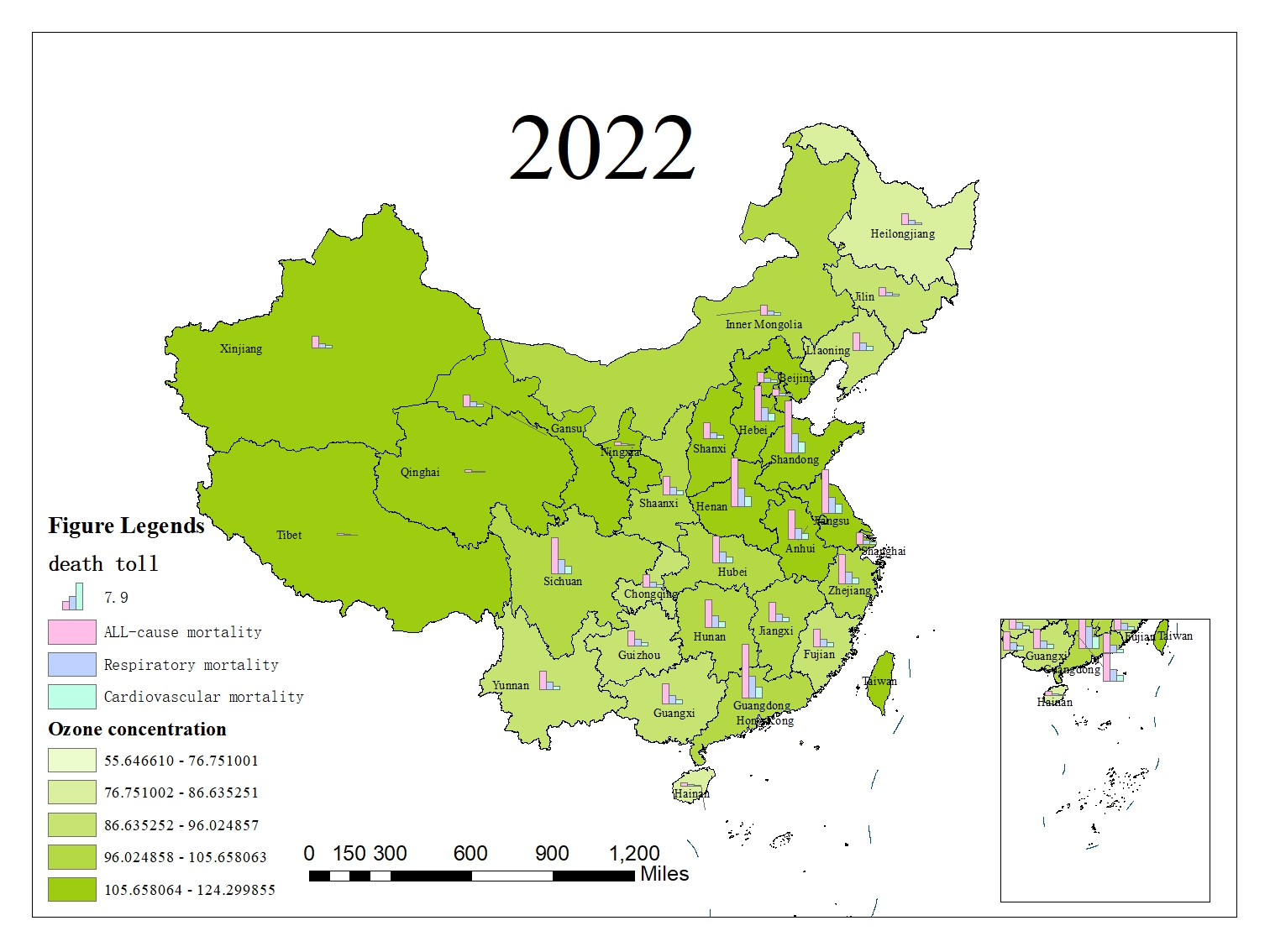

Supplement: Supplementary file 3 [file Data_Sheet_3.zip › O3/O3 0μgm3/2022.jpg]

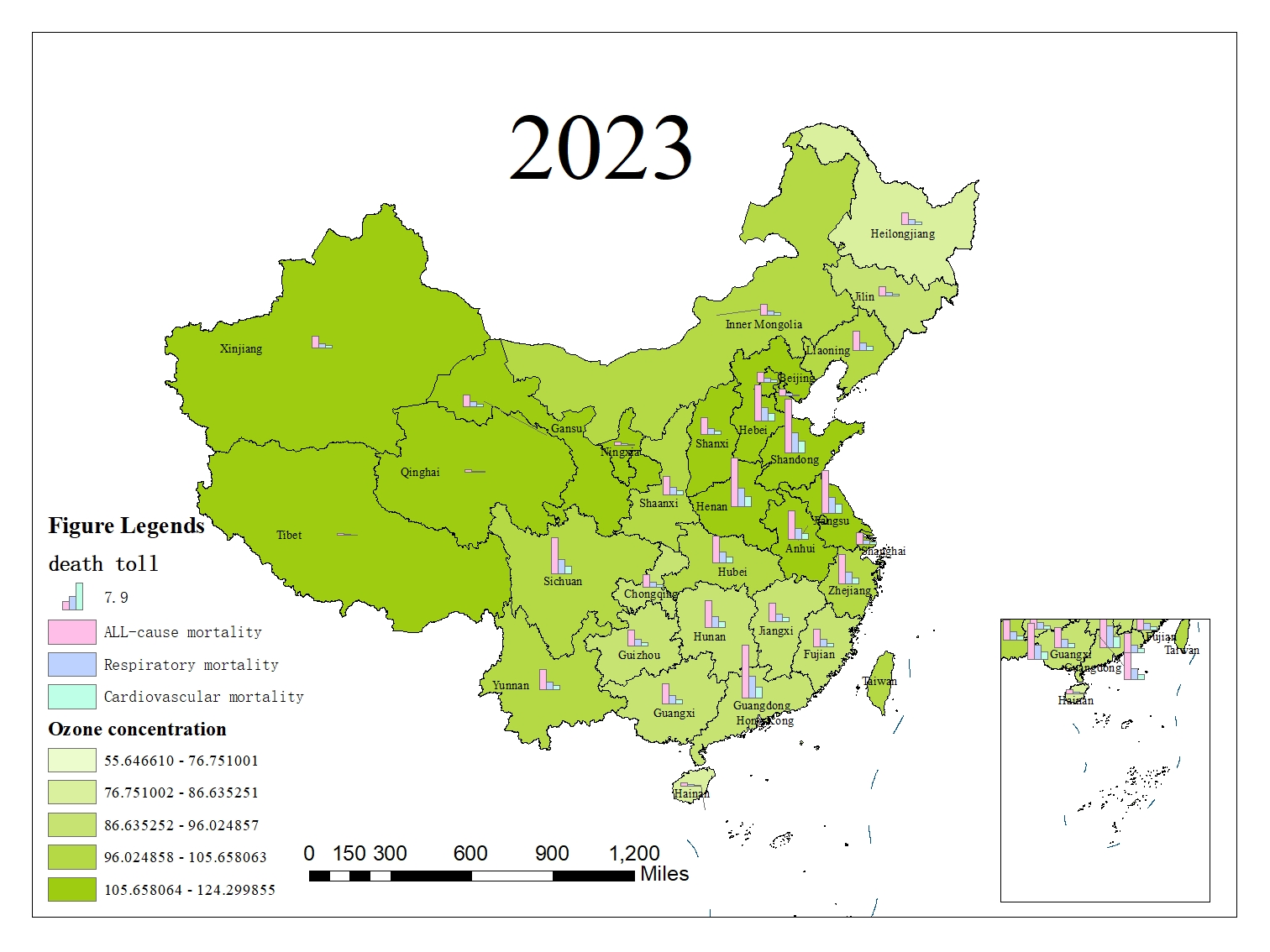

Supplement: Supplementary file 3 [file Data_Sheet_3.zip › O3/O3 0μgm3/2023.jpg]

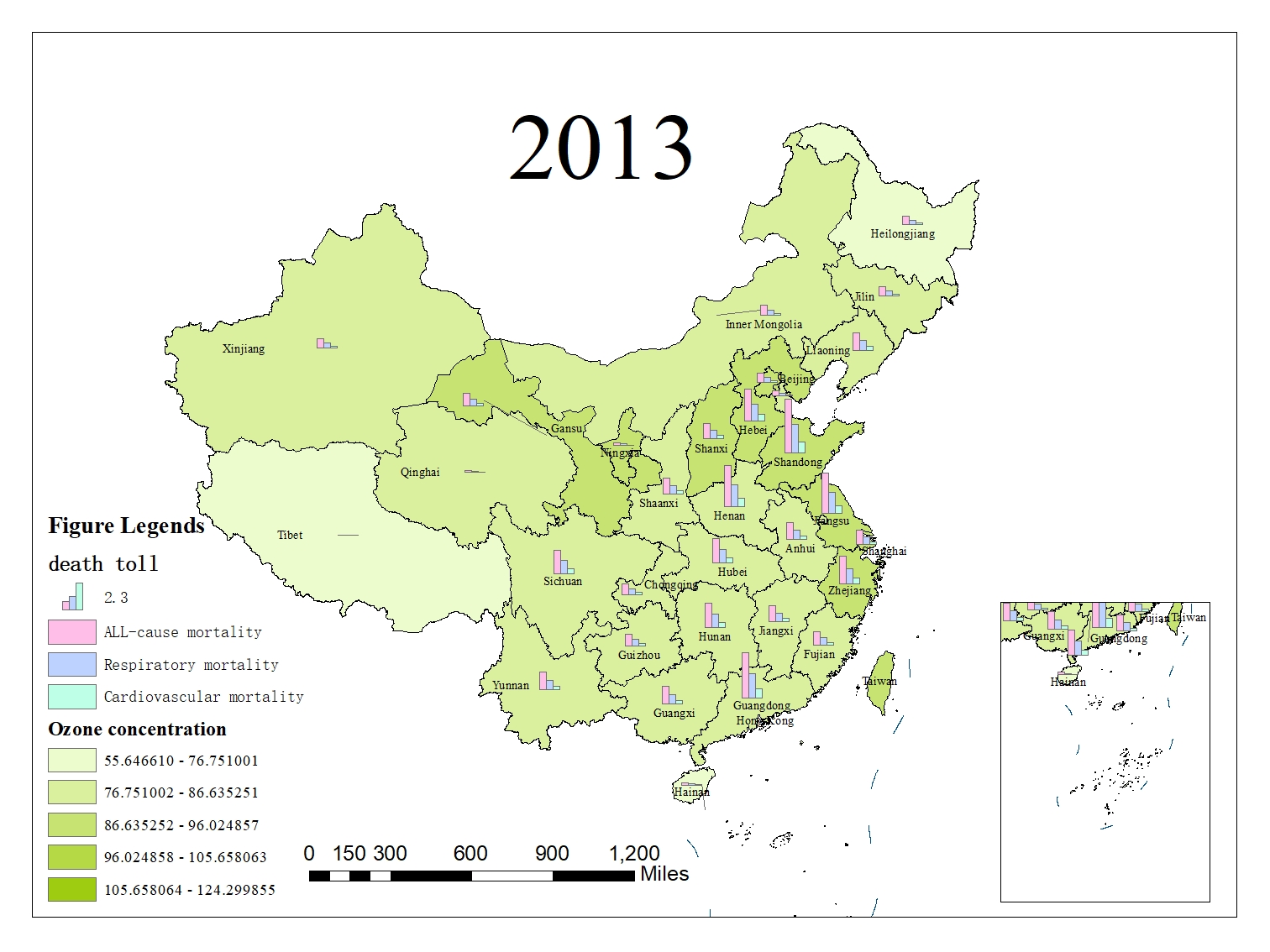

Supplement: Supplementary file 3 [file Data_Sheet_3.zip › O3/O3 60μgm3/2013.jpg]

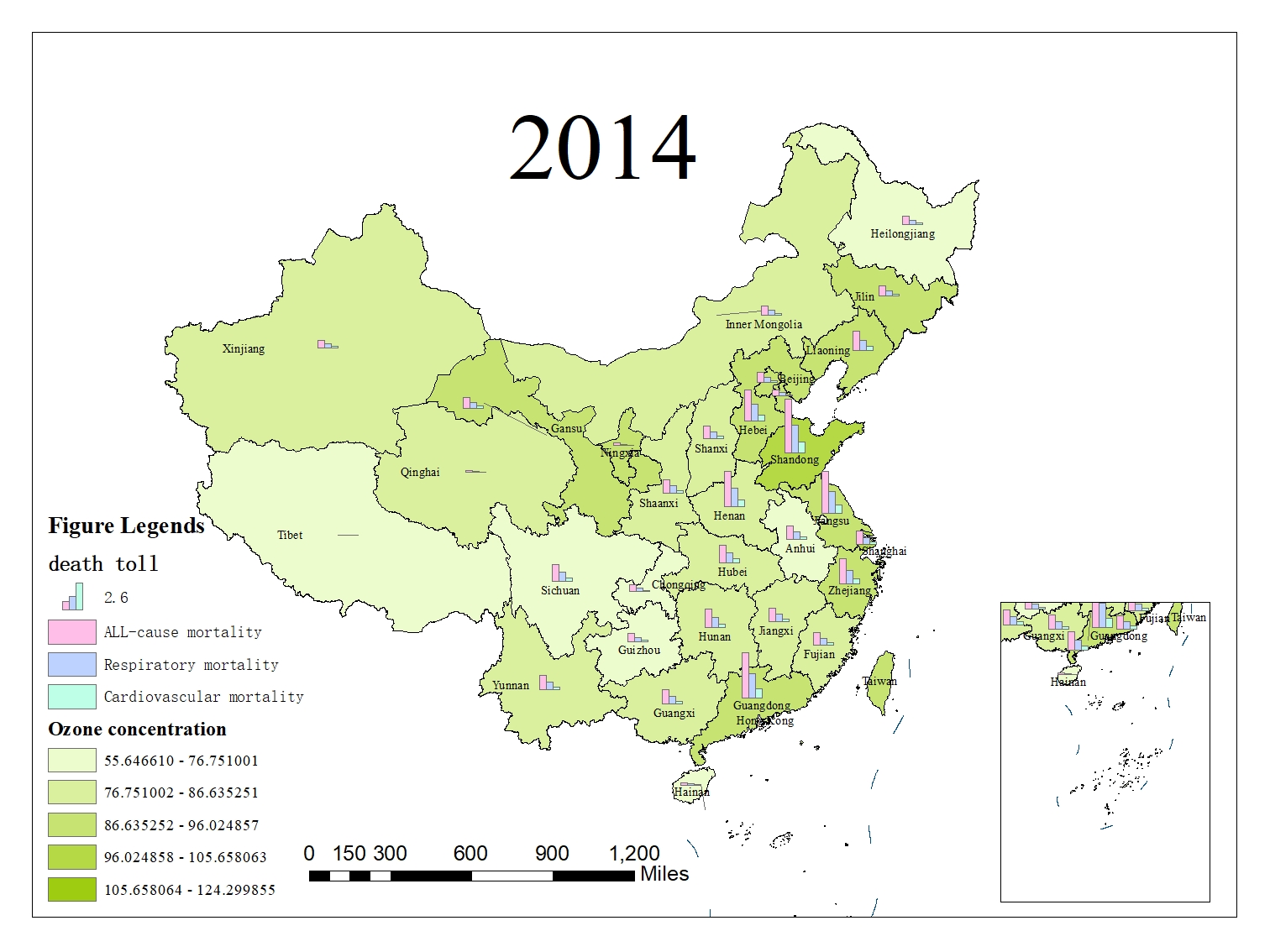

Supplement: Supplementary file 3 [file Data_Sheet_3.zip › O3/O3 60μgm3/2014.jpg]

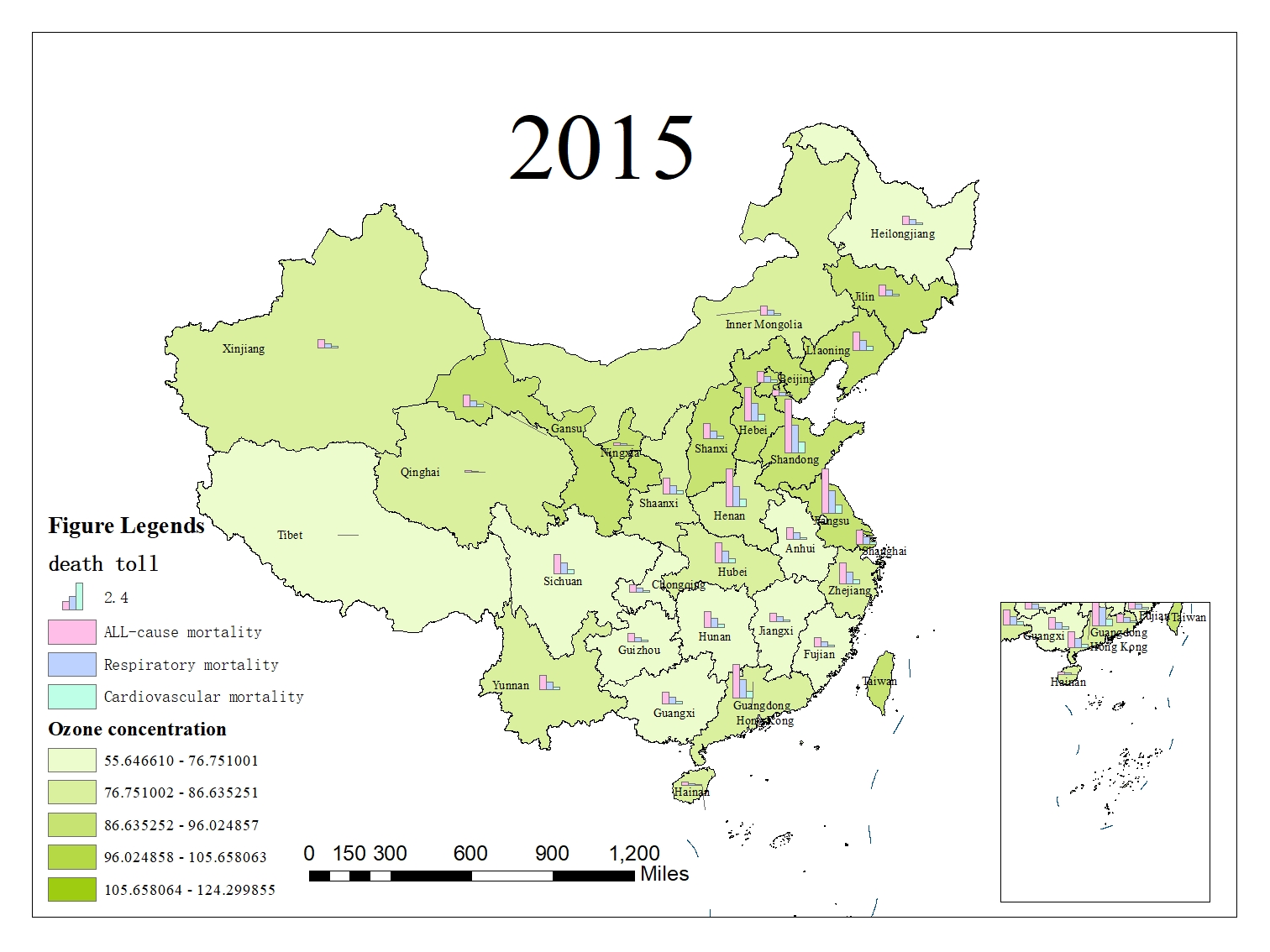

Supplement: Supplementary file 3 [file Data_Sheet_3.zip › O3/O3 60μgm3/2015.jpg]

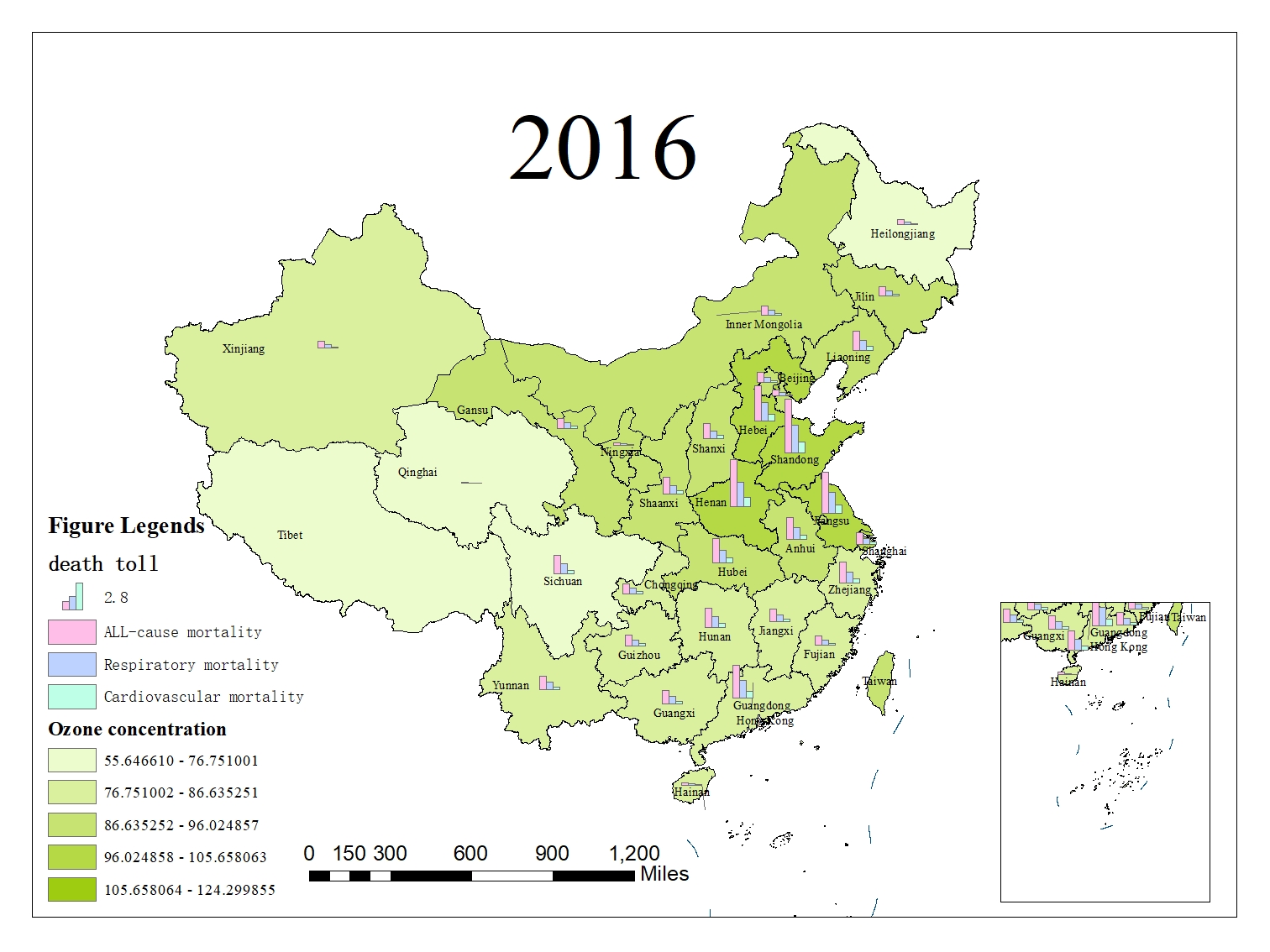

Supplement: Supplementary file 3 [file Data_Sheet_3.zip › O3/O3 60μgm3/2016.jpg]

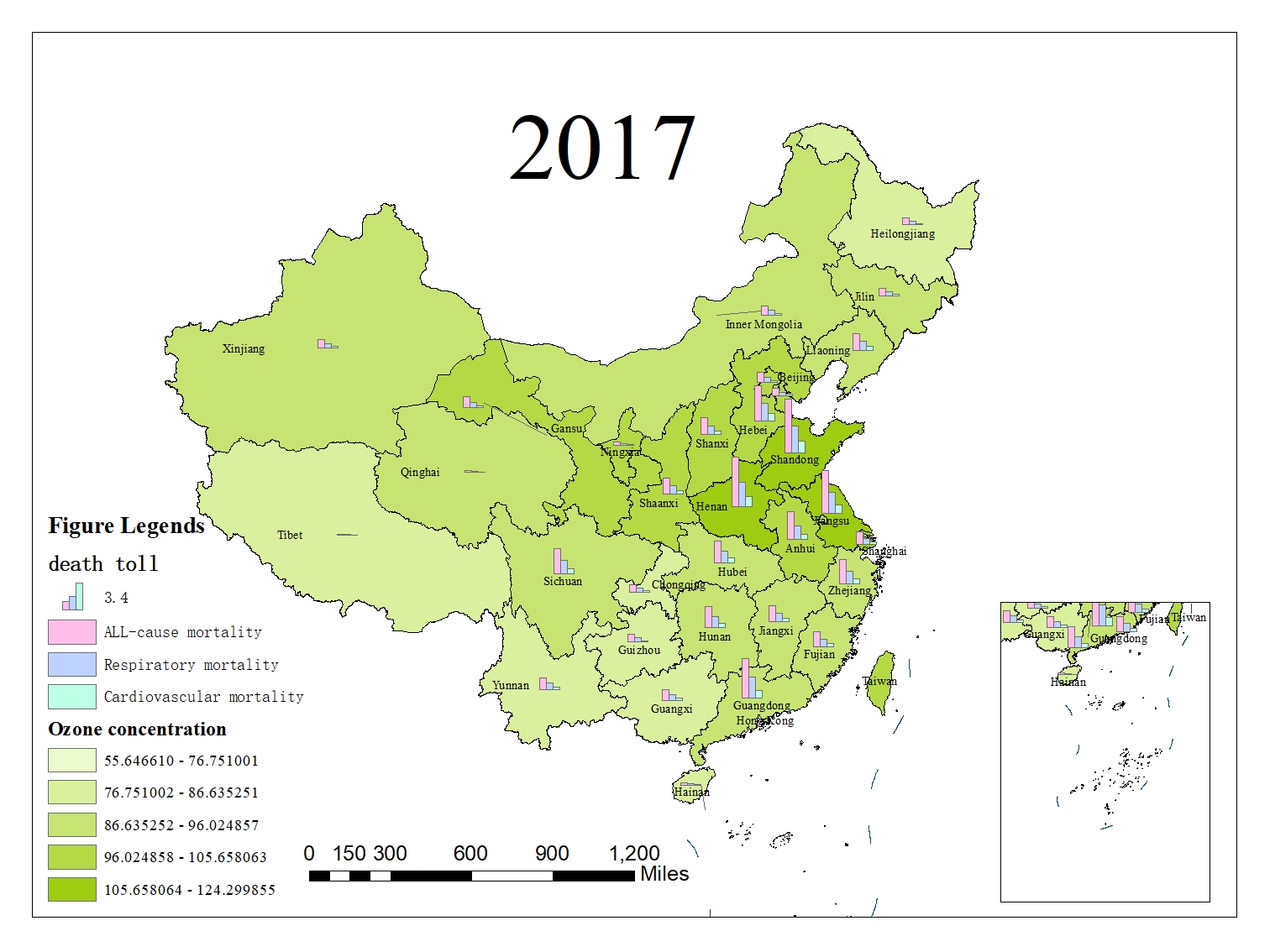

Supplement: Supplementary file 3 [file Data_Sheet_3.zip › O3/O3 60μgm3/2017.jpg]

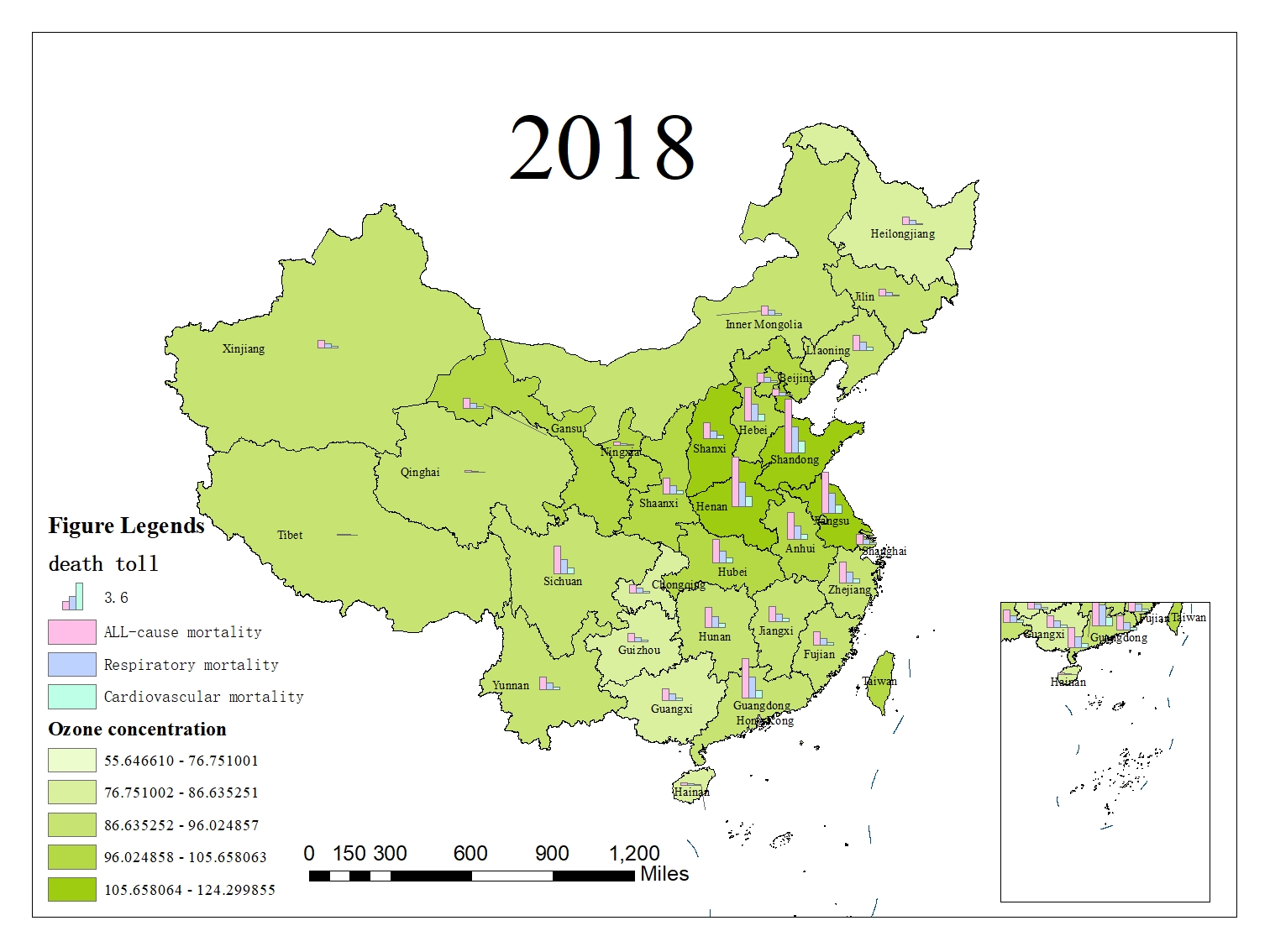

Supplement: Supplementary file 3 [file Data_Sheet_3.zip › O3/O3 60μgm3/2018.jpg]

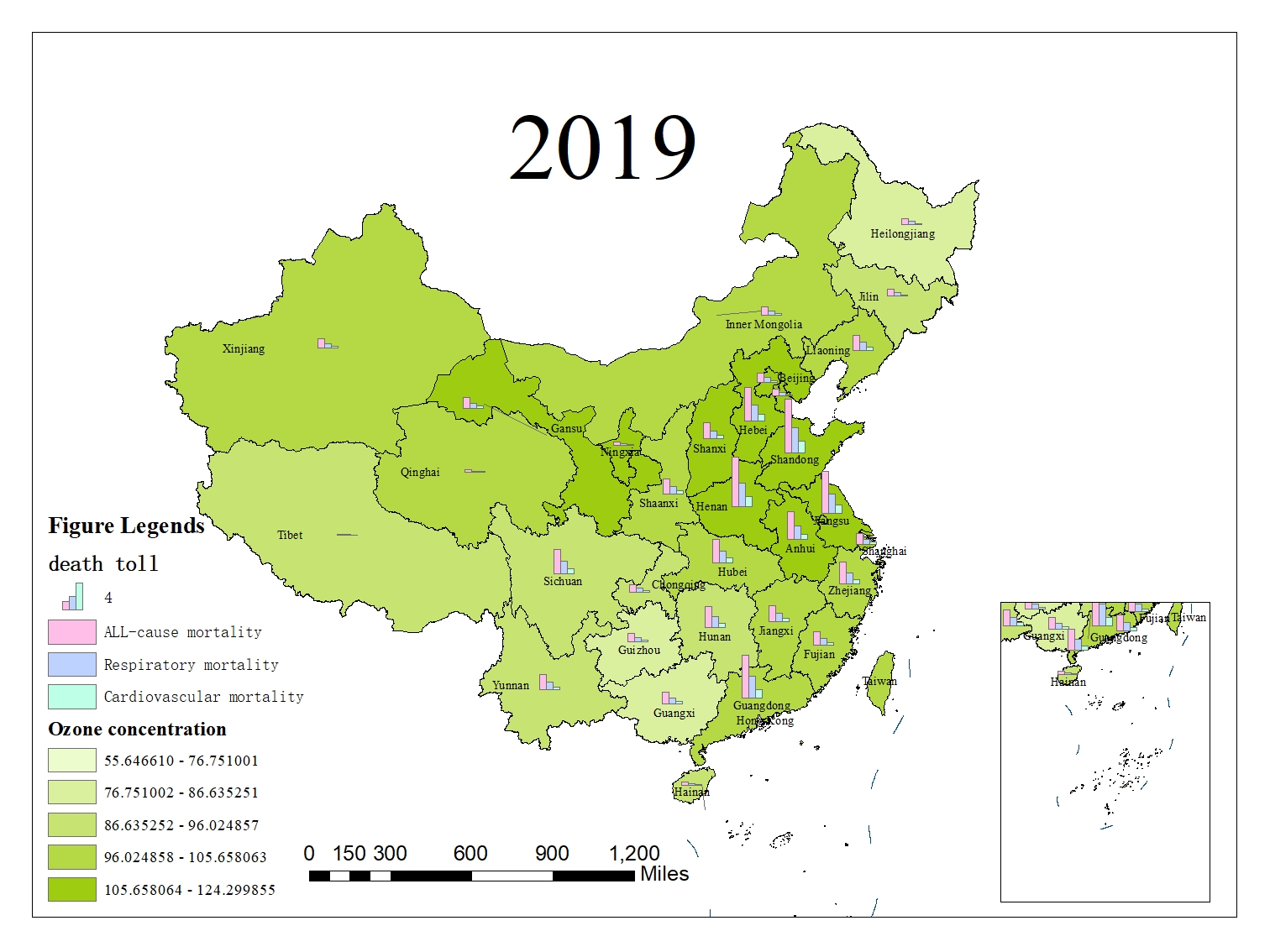

Supplement: Supplementary file 3 [file Data_Sheet_3.zip › O3/O3 60μgm3/2019.jpg]

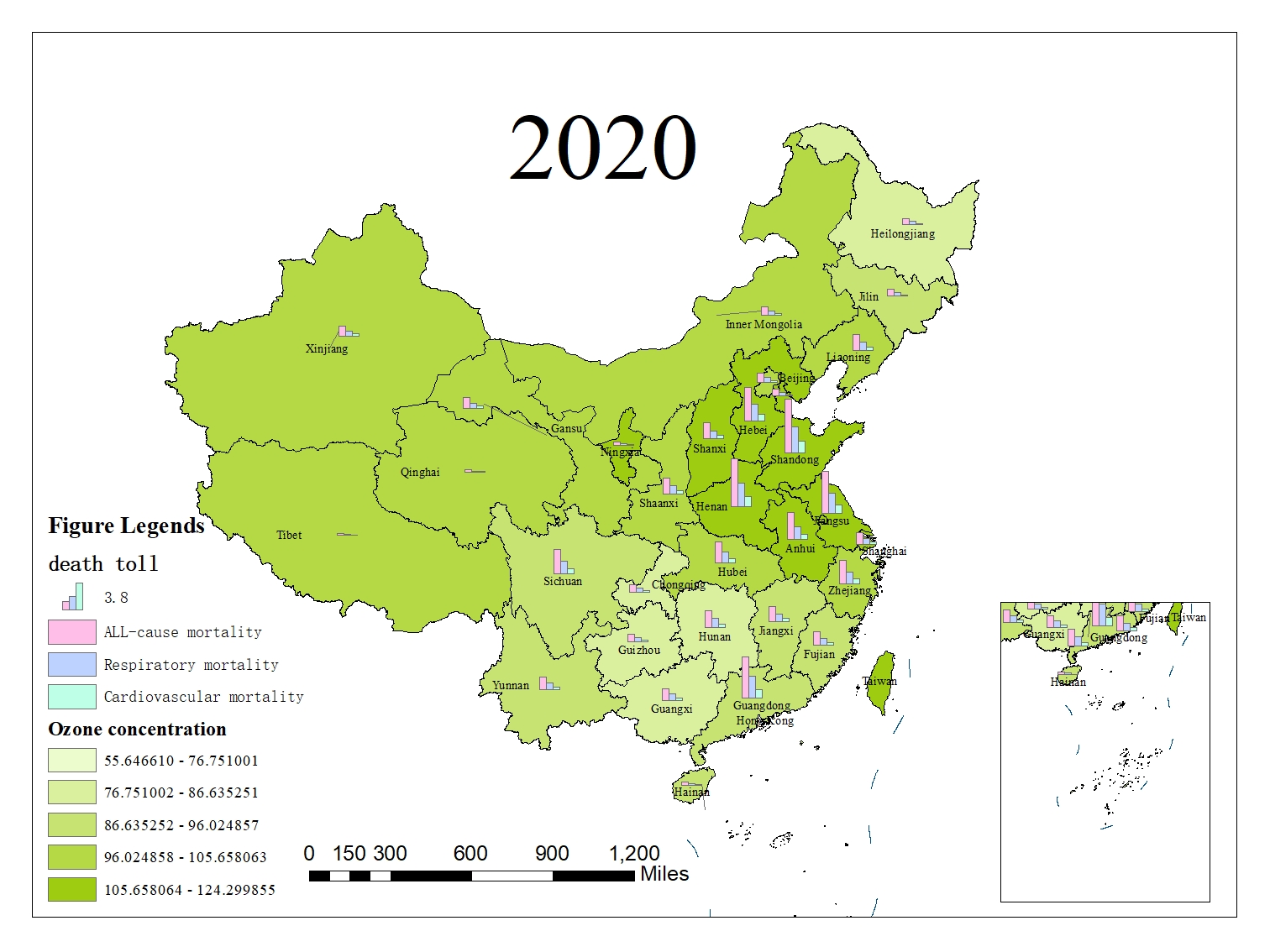

Supplement: Supplementary file 3 [file Data_Sheet_3.zip › O3/O3 60μgm3/2020.jpg]

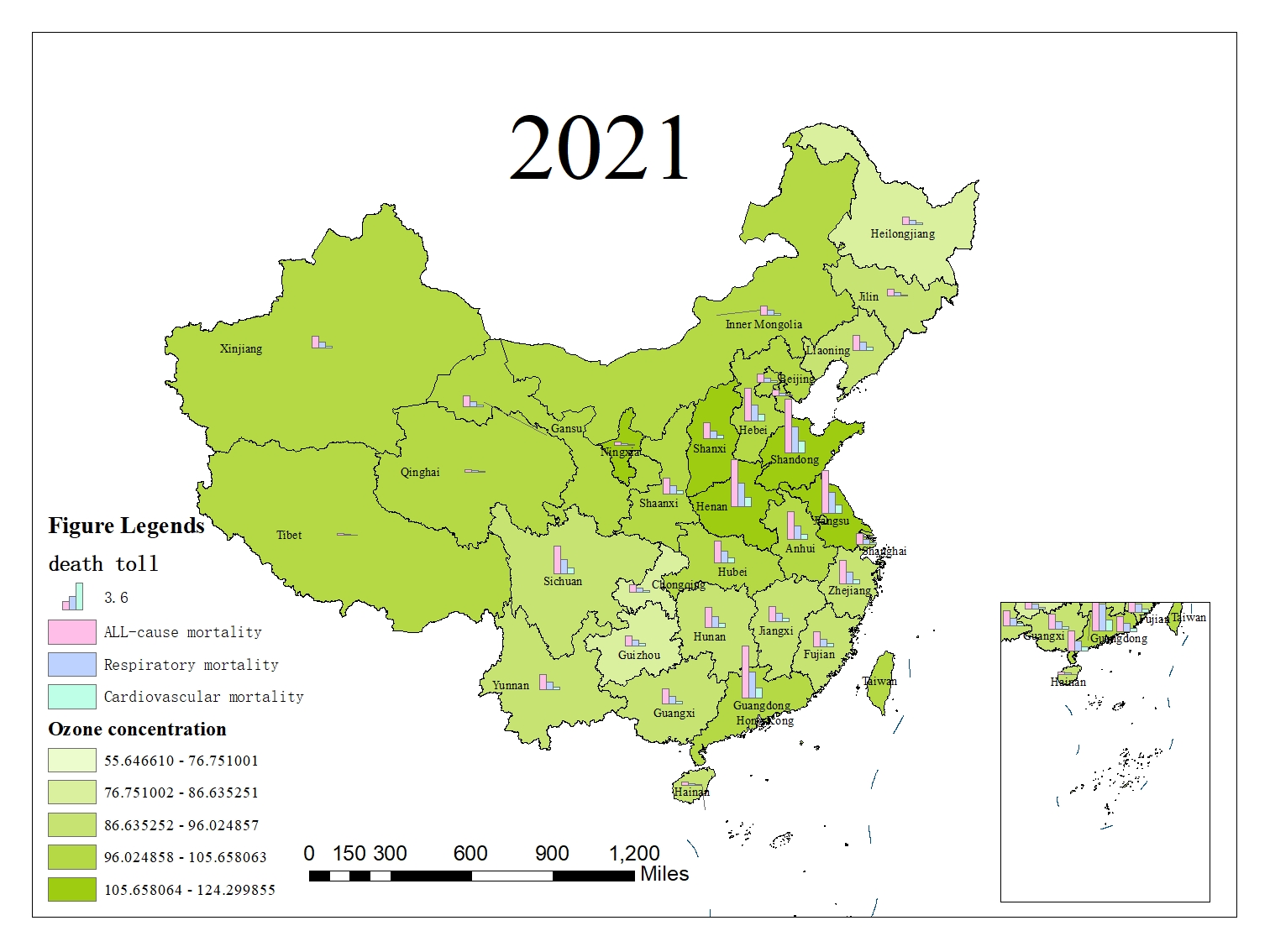

Supplement: Supplementary file 3 [file Data_Sheet_3.zip › O3/O3 60μgm3/2021.jpg]

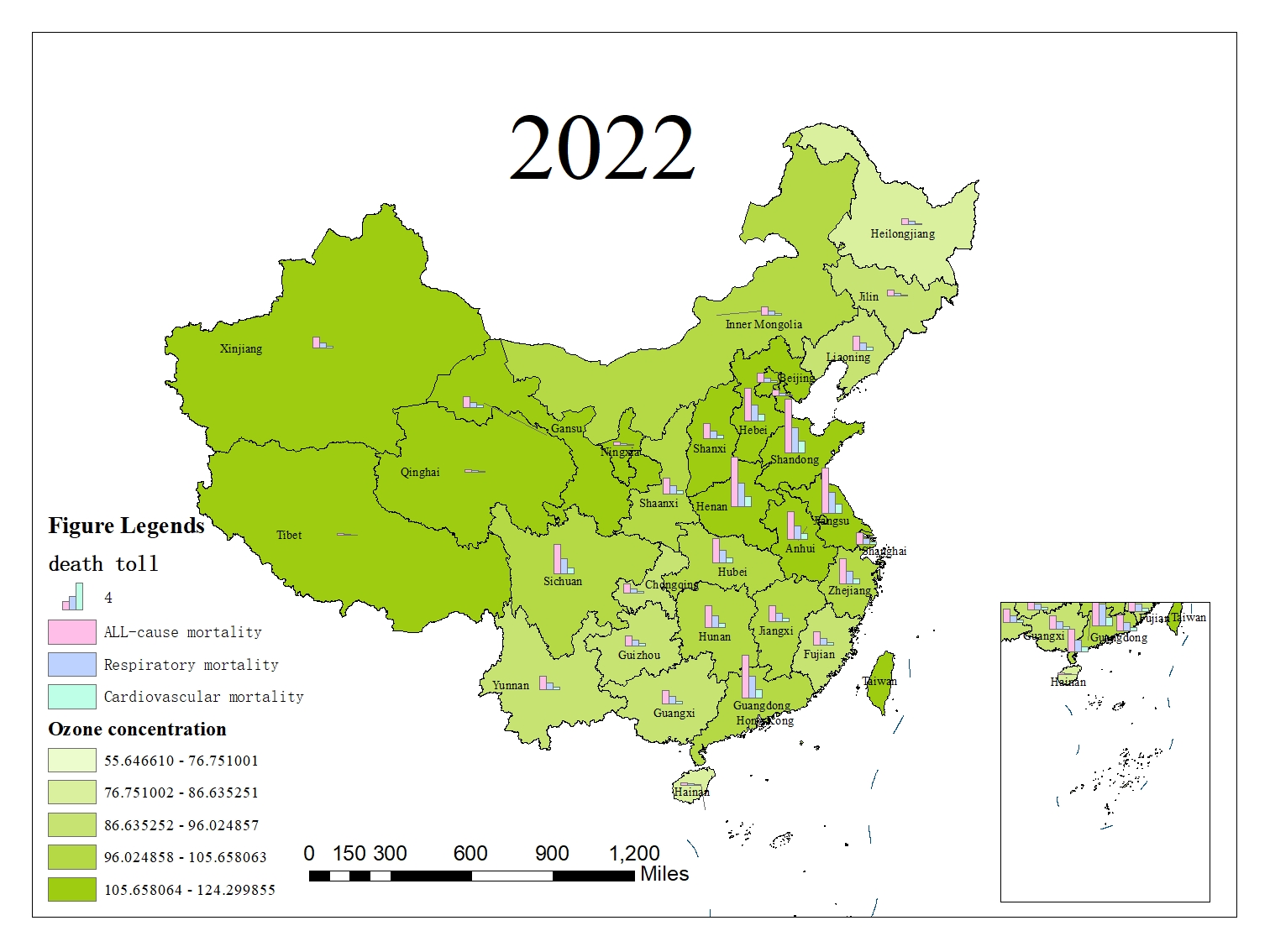

Supplement: Supplementary file 3 [file Data_Sheet_3.zip › O3/O3 60μgm3/2022.jpg]

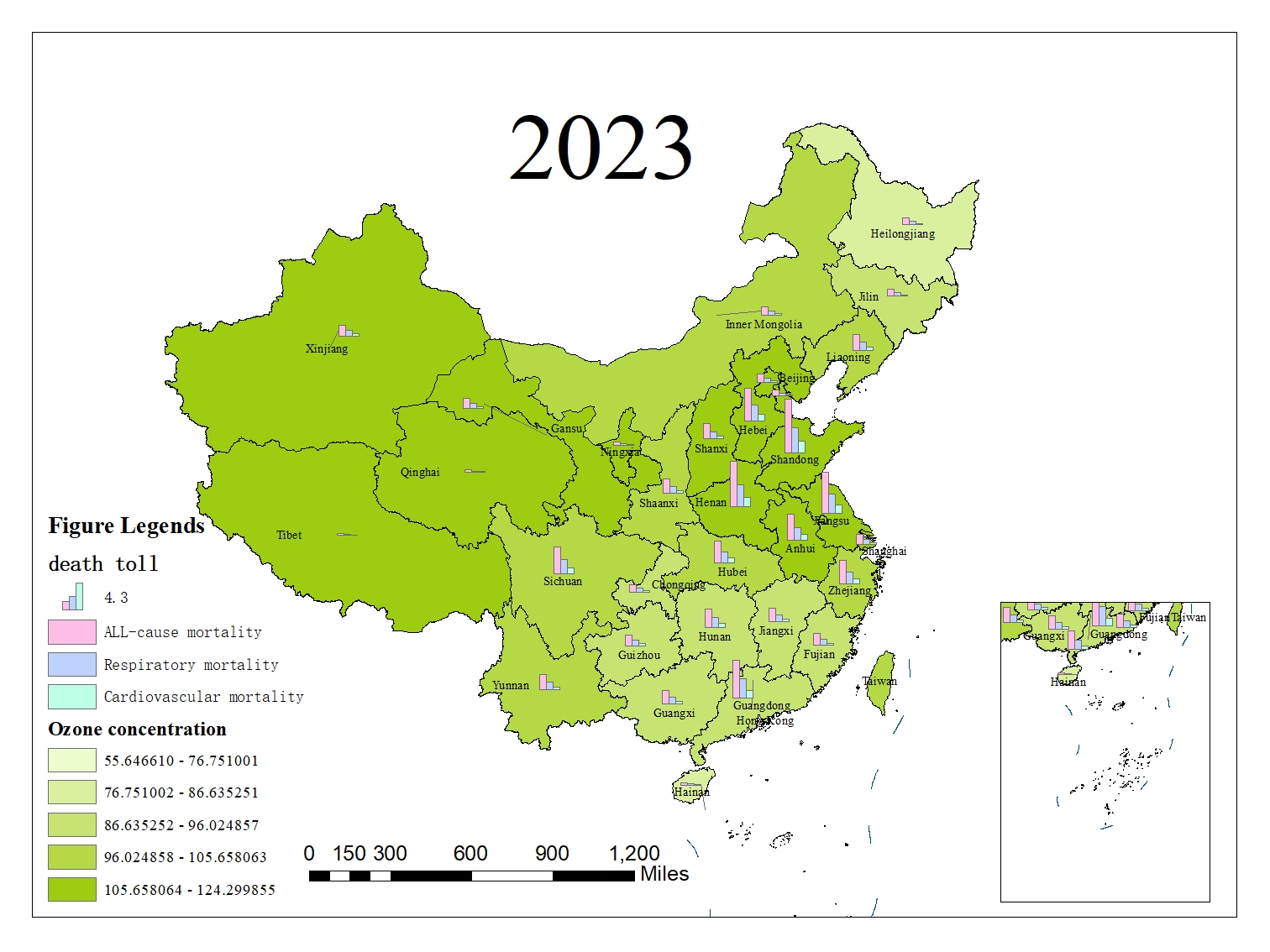

Supplement: Supplementary file 3 [file Data_Sheet_3.zip › O3/O3 60μgm3/2023.jpg]

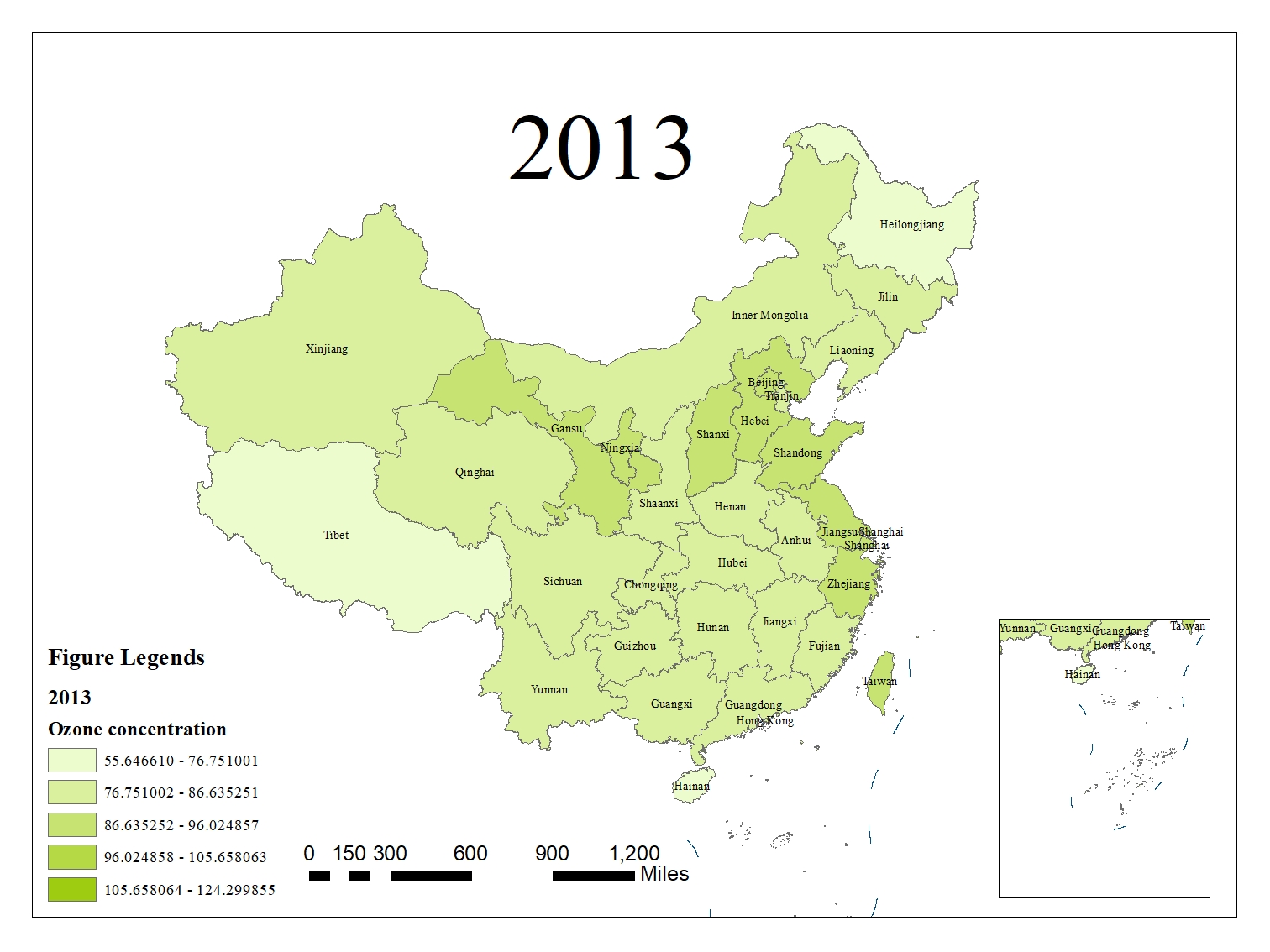

Supplement: Supplementary file 3 [file Data_Sheet_3.zip › O3/O3 concentration/2013.jpg]

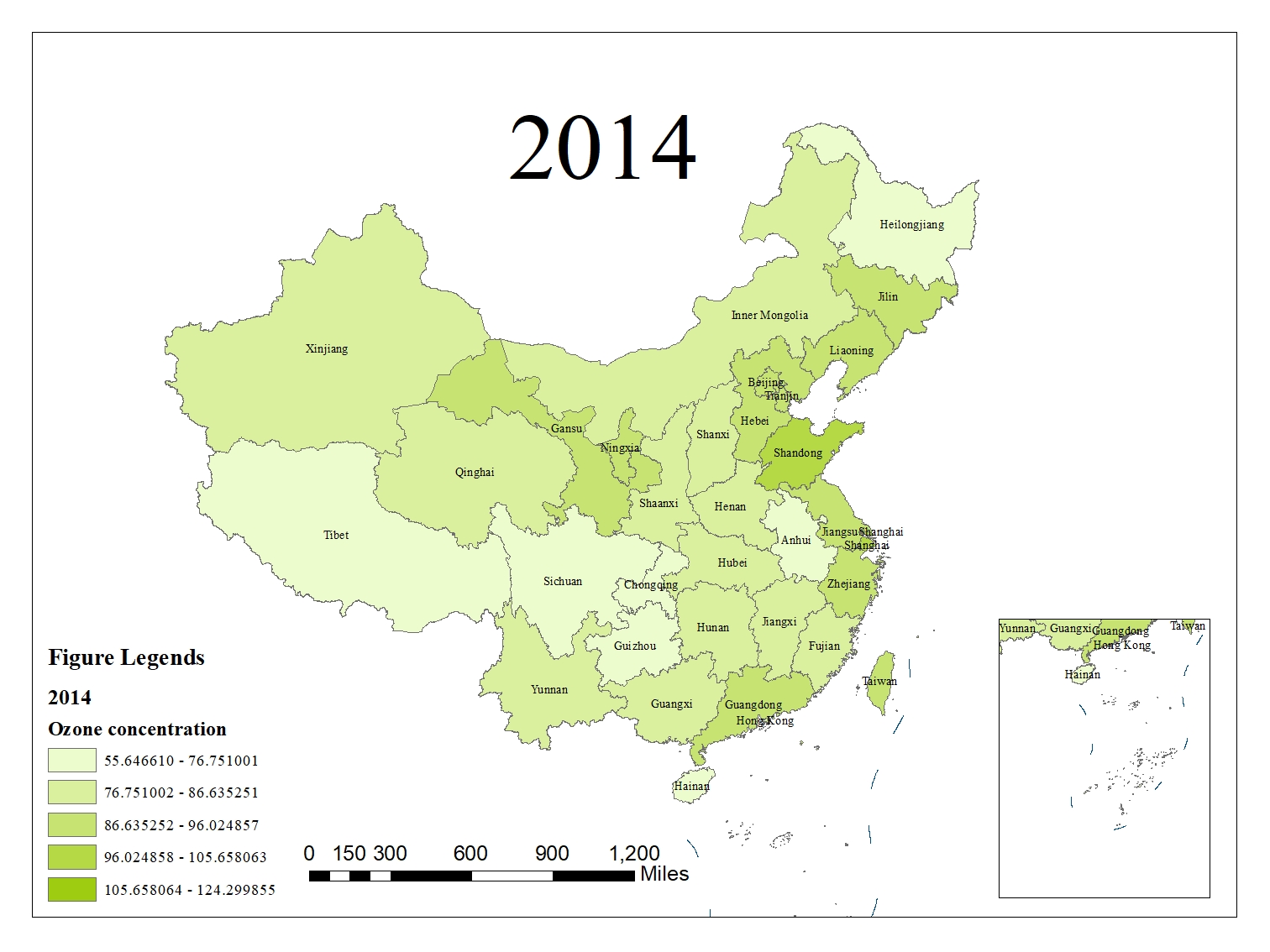

Supplement: Supplementary file 3 [file Data_Sheet_3.zip › O3/O3 concentration/2014.jpg]

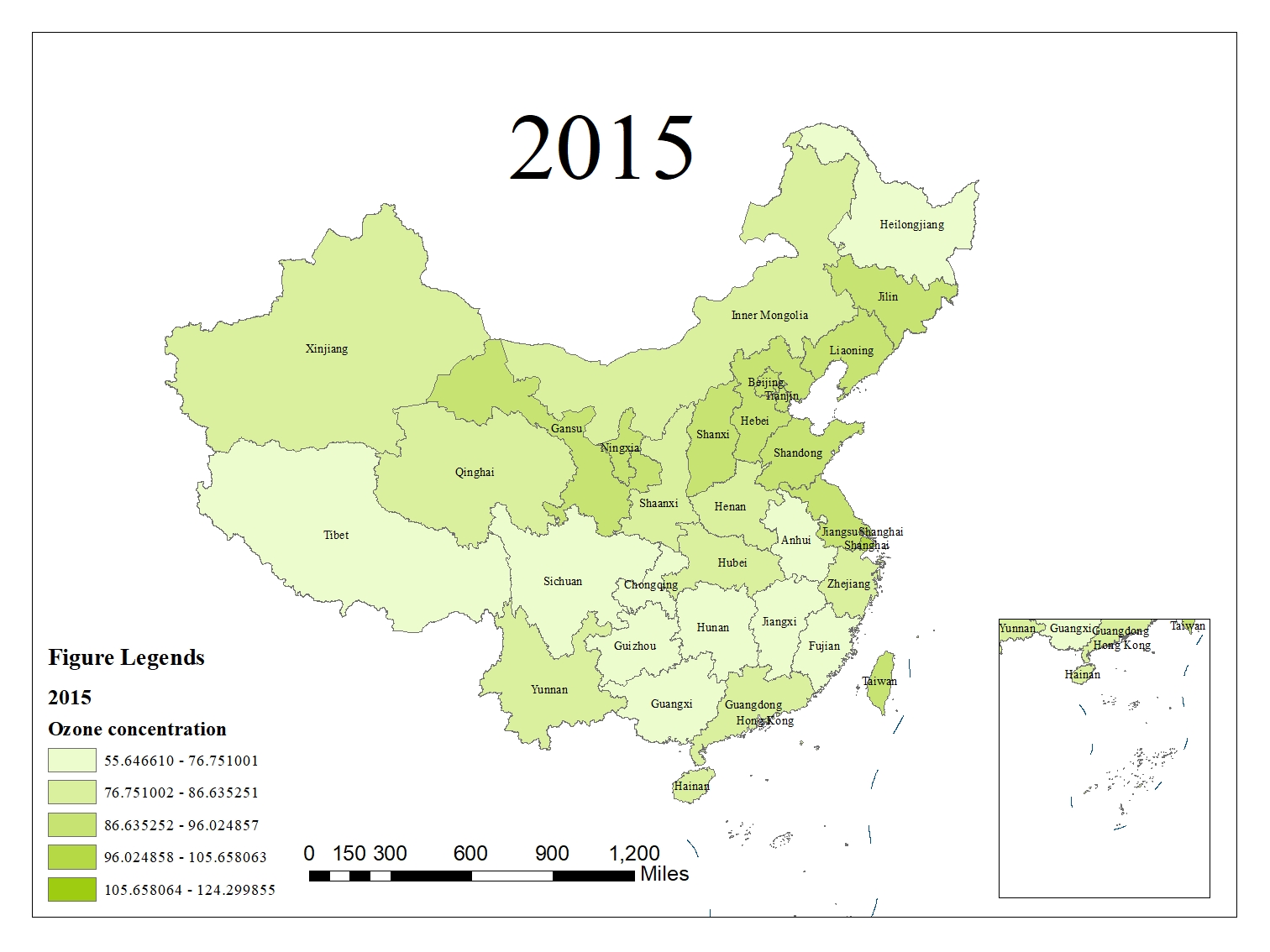

Supplement: Supplementary file 3 [file Data_Sheet_3.zip › O3/O3 concentration/2015.jpg]

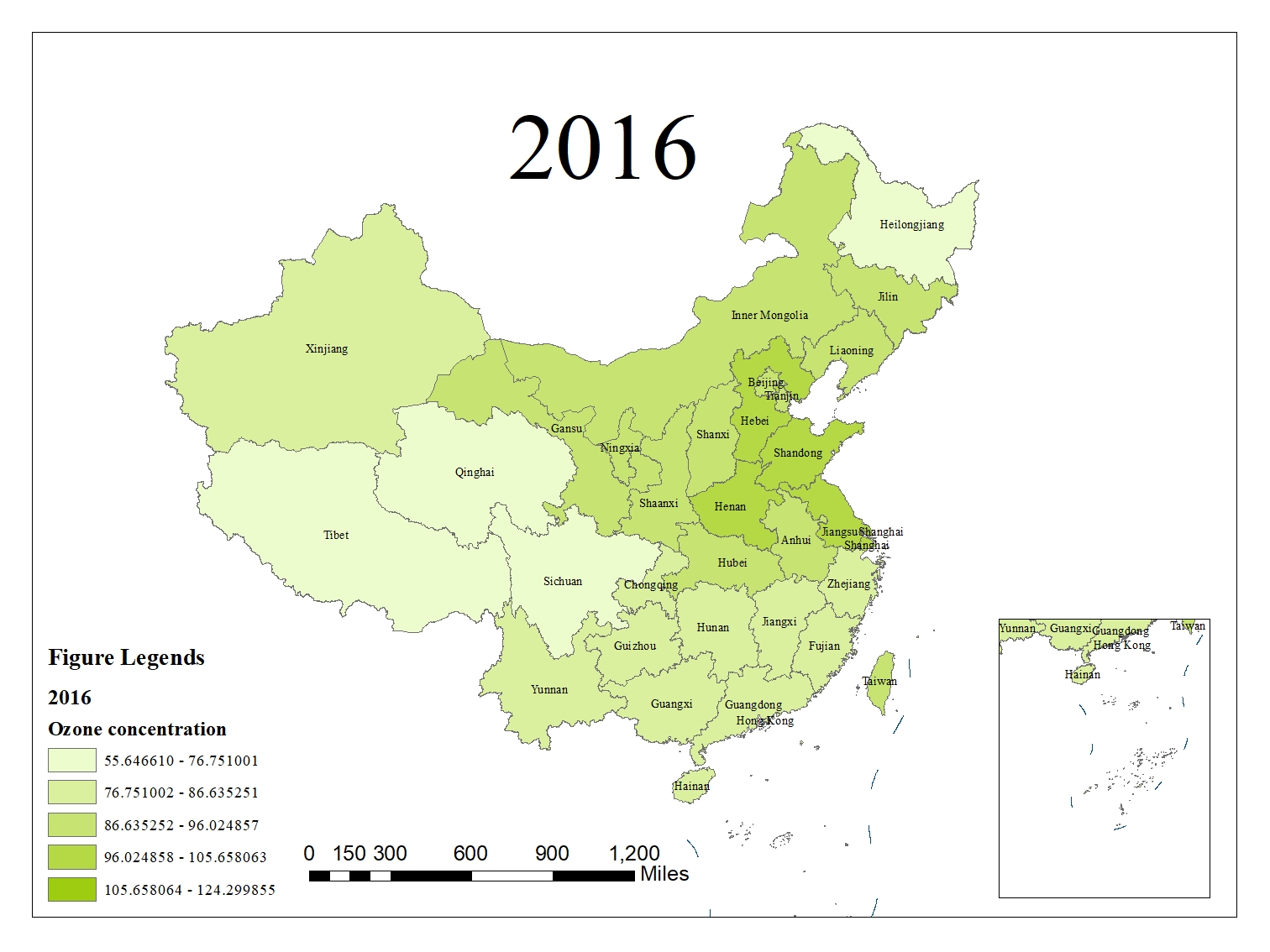

Supplement: Supplementary file 3 [file Data_Sheet_3.zip › O3/O3 concentration/2016.jpg]

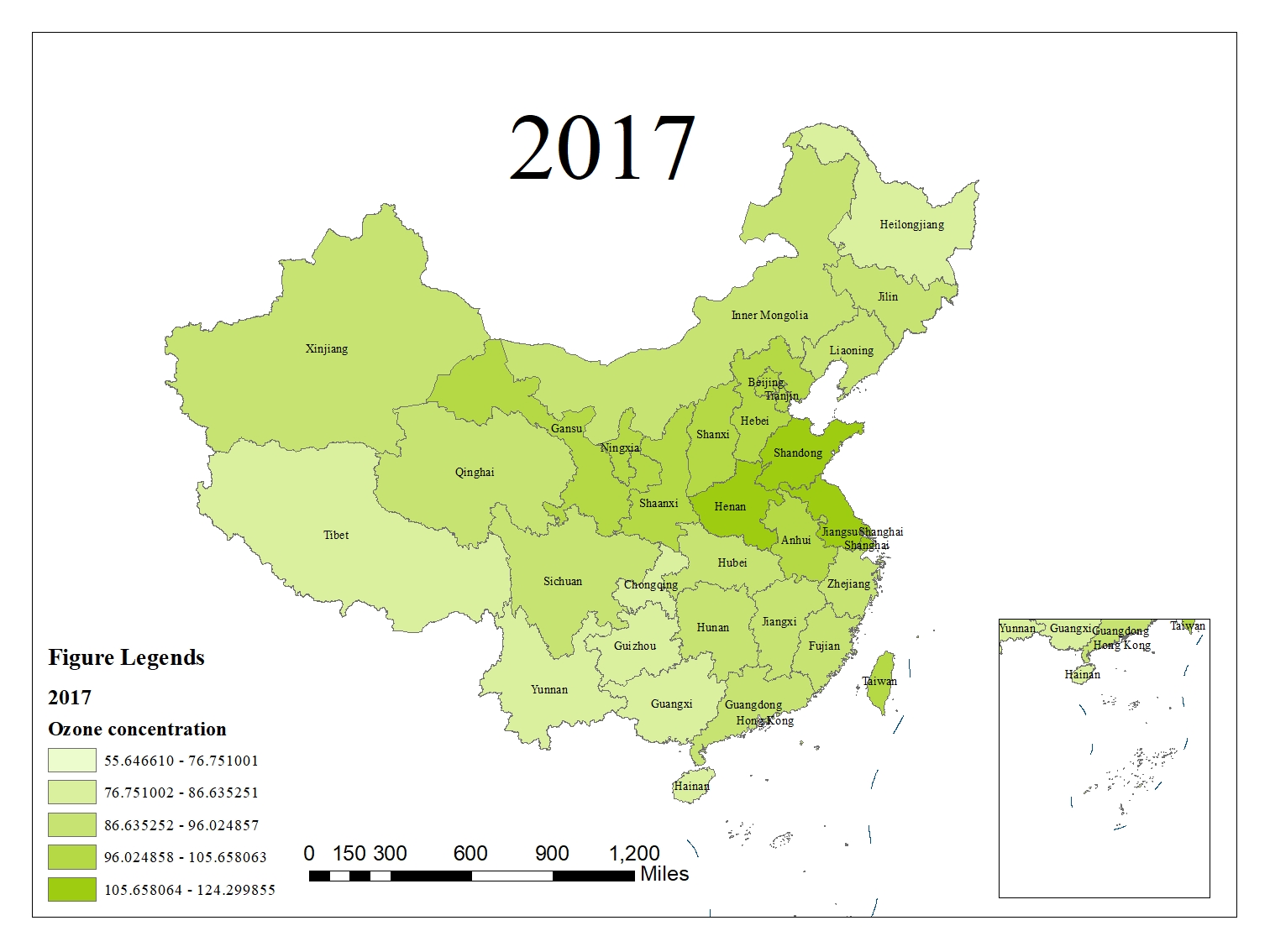

Supplement: Supplementary file 3 [file Data_Sheet_3.zip › O3/O3 concentration/2017.jpg]

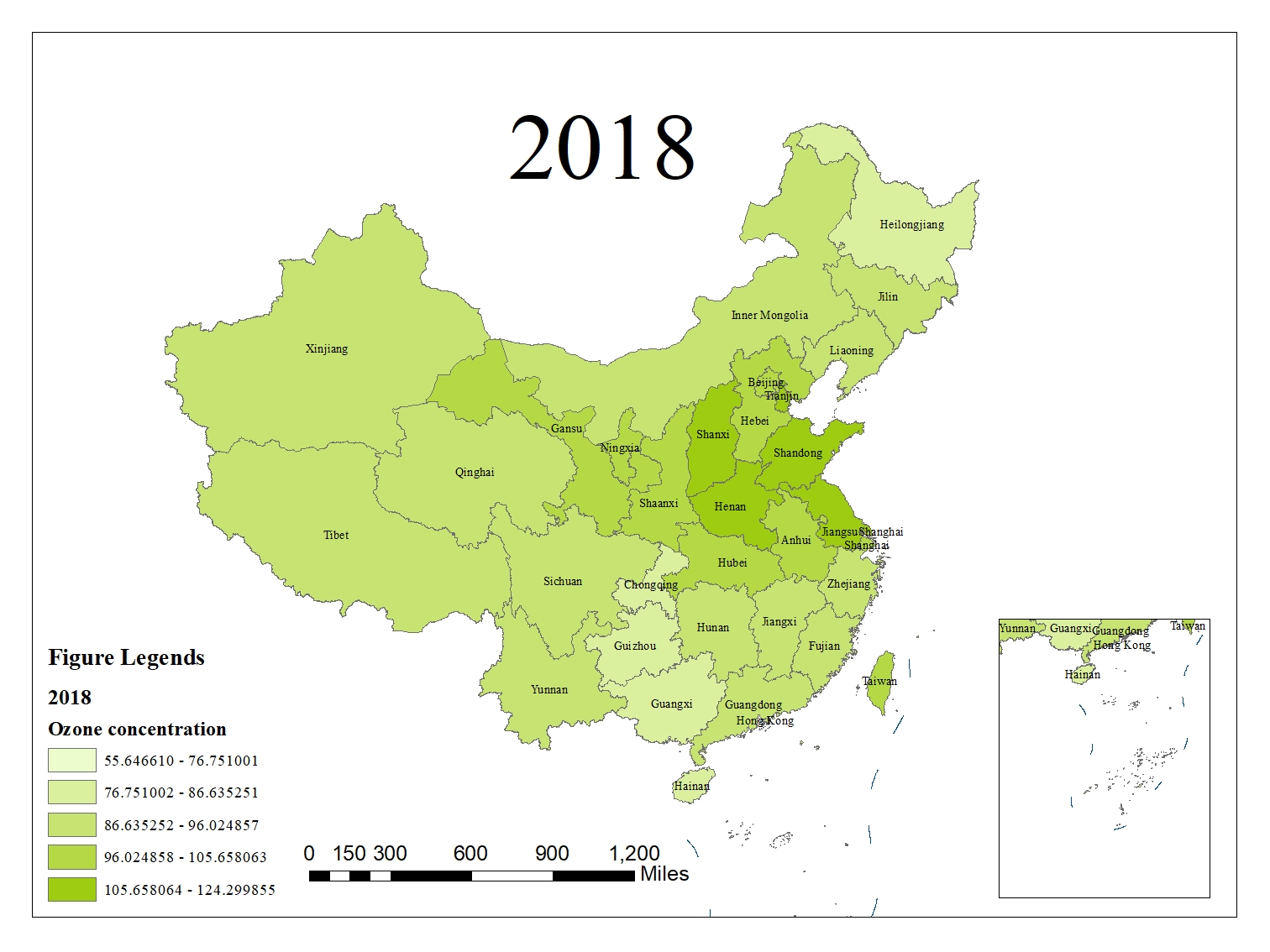

Supplement: Supplementary file 3 [file Data_Sheet_3.zip › O3/O3 concentration/2018.jpg]

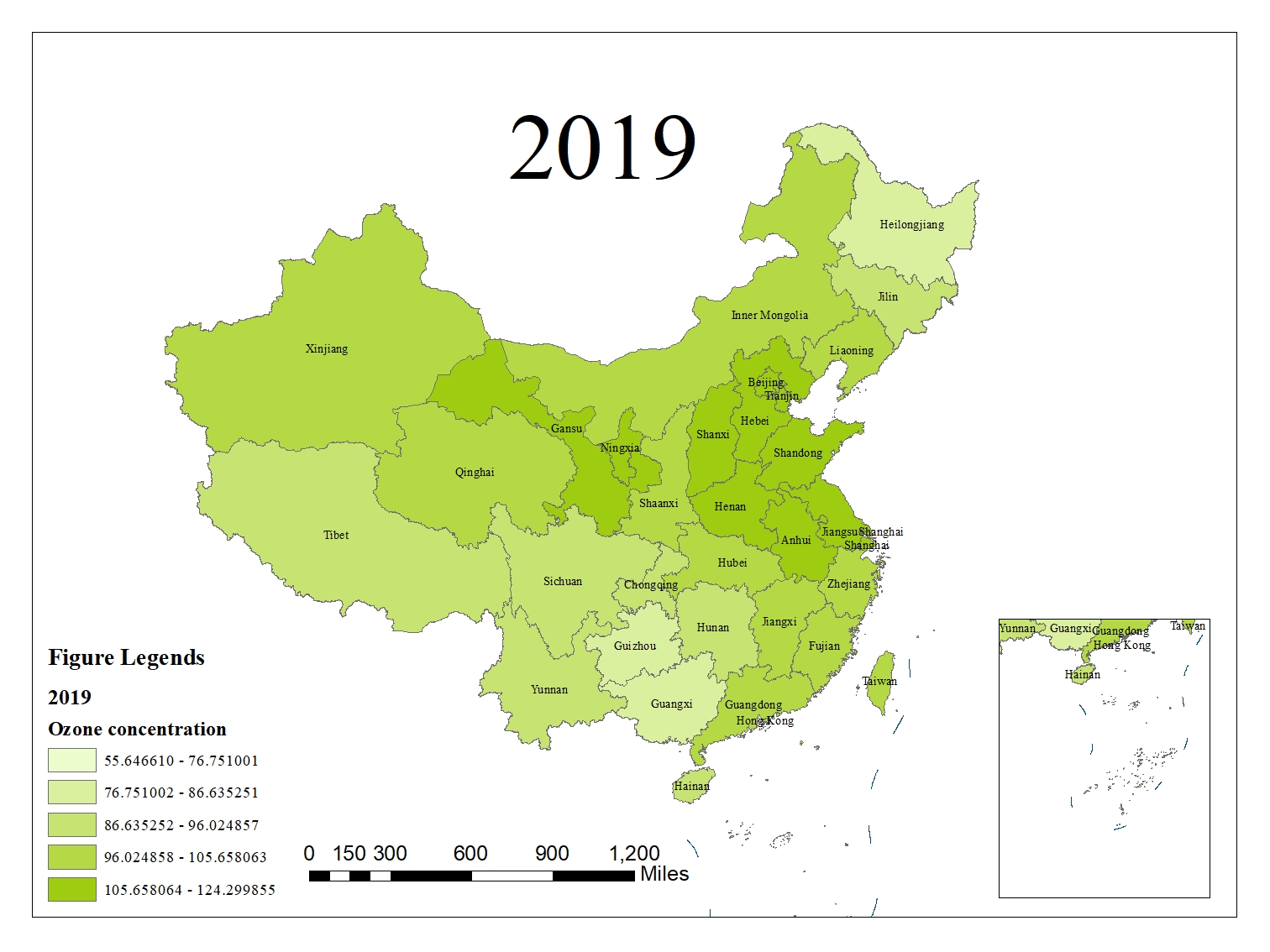

Supplement: Supplementary file 3 [file Data_Sheet_3.zip › O3/O3 concentration/2019.jpg]

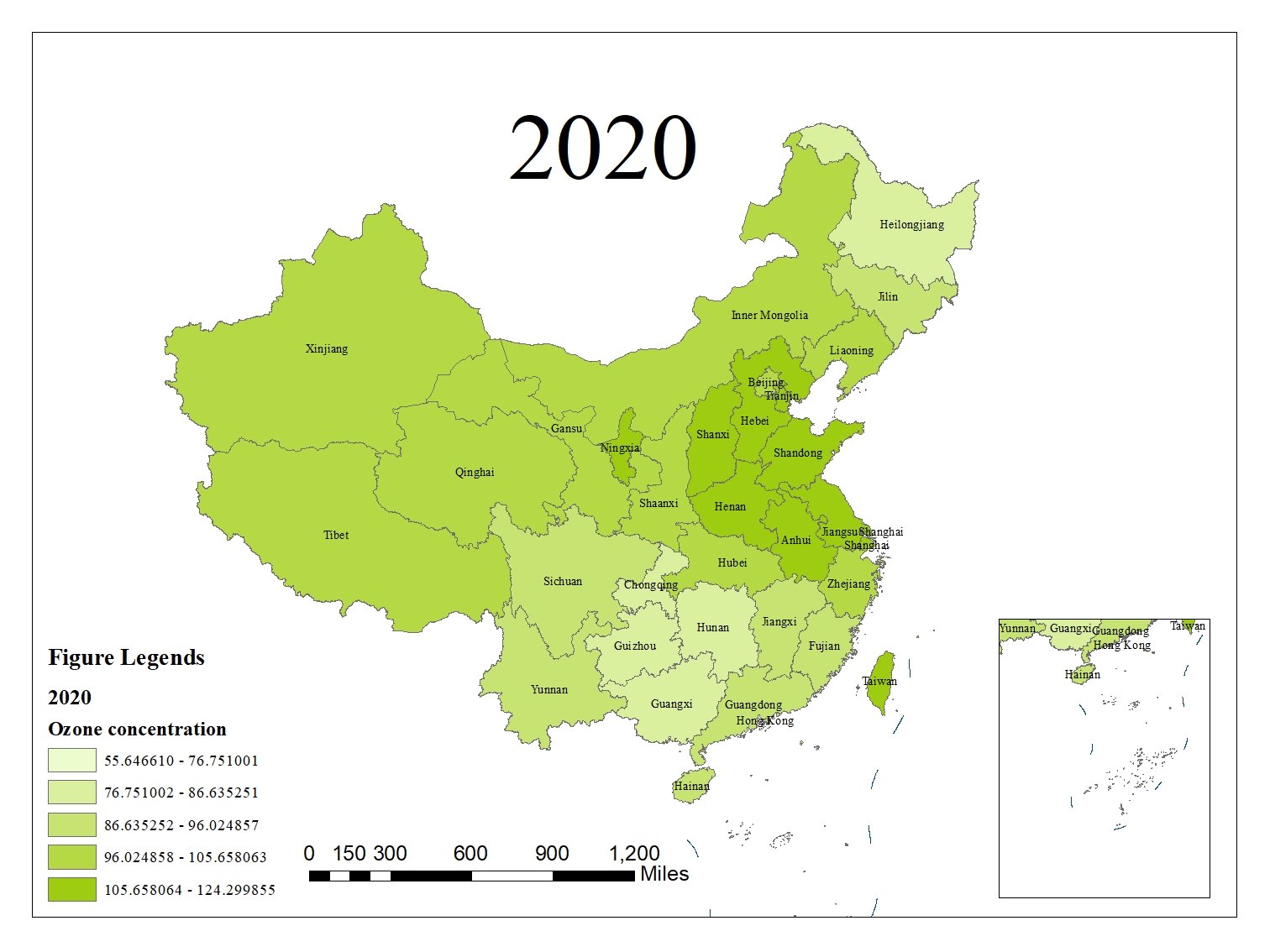

Supplement: Supplementary file 3 [file Data_Sheet_3.zip › O3/O3 concentration/2020.jpg]

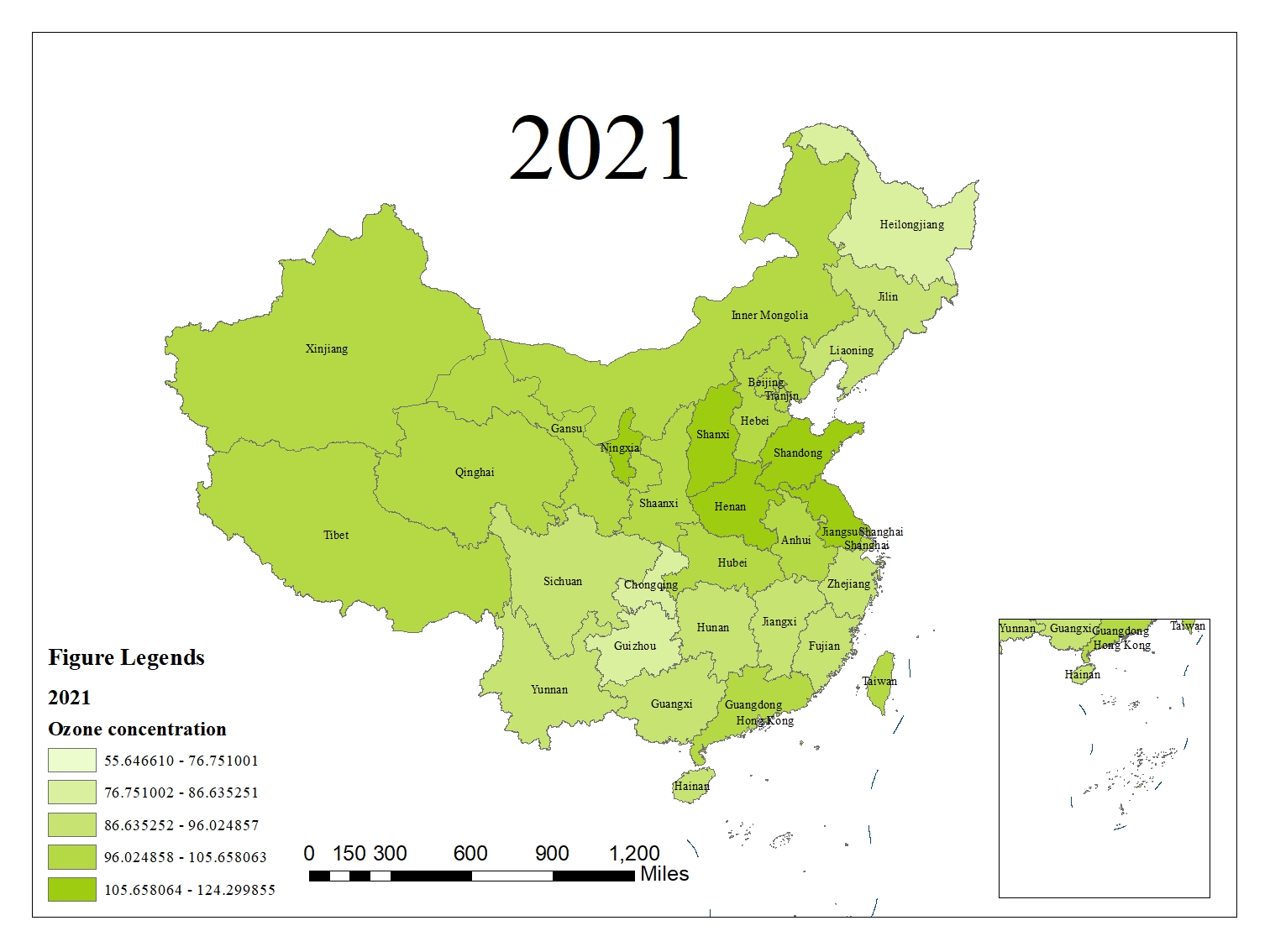

Supplement: Supplementary file 3 [file Data_Sheet_3.zip › O3/O3 concentration/2021.jpg]

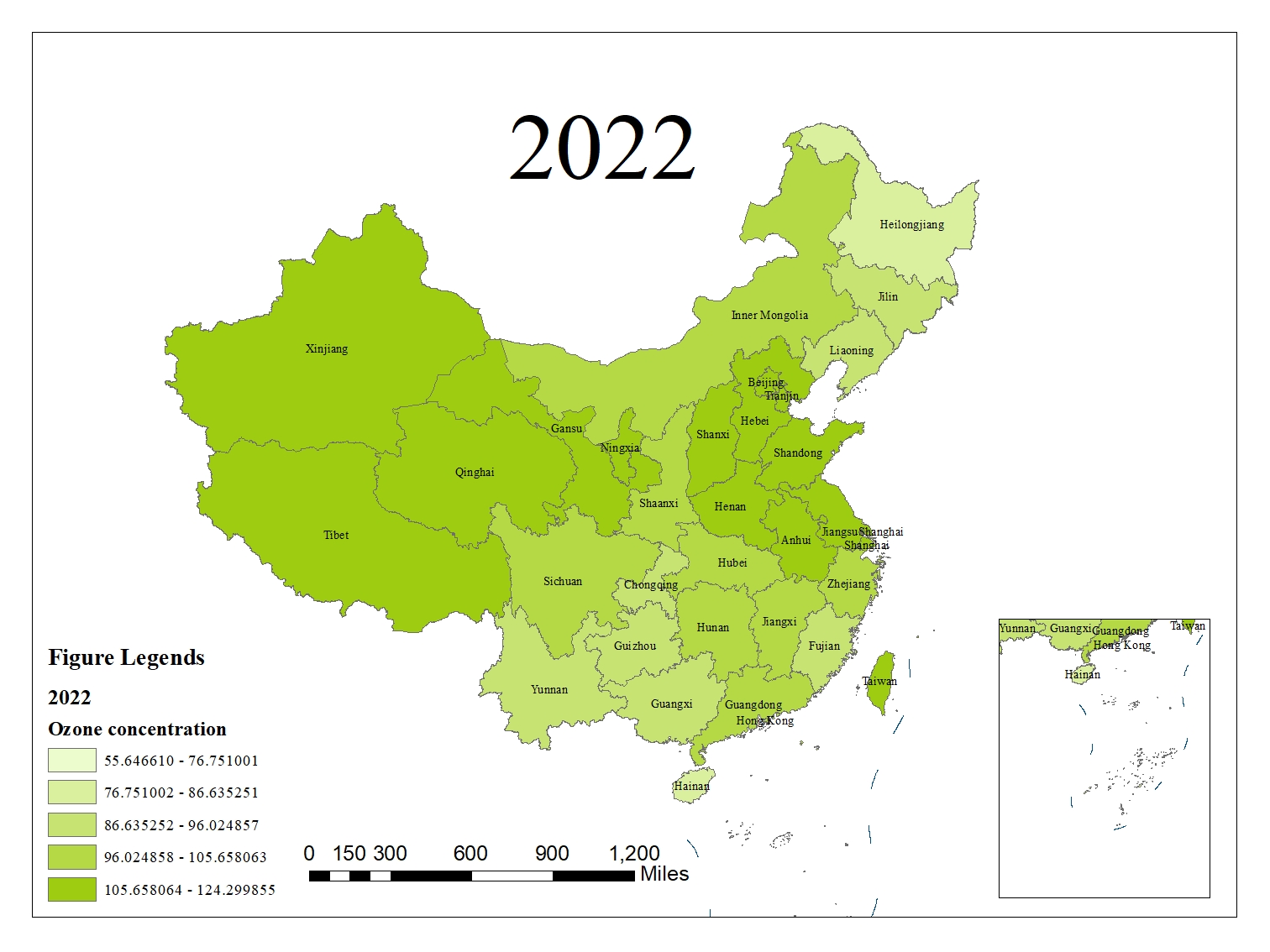

Supplement: Supplementary file 3 [file Data_Sheet_3.zip › O3/O3 concentration/2022.jpg]

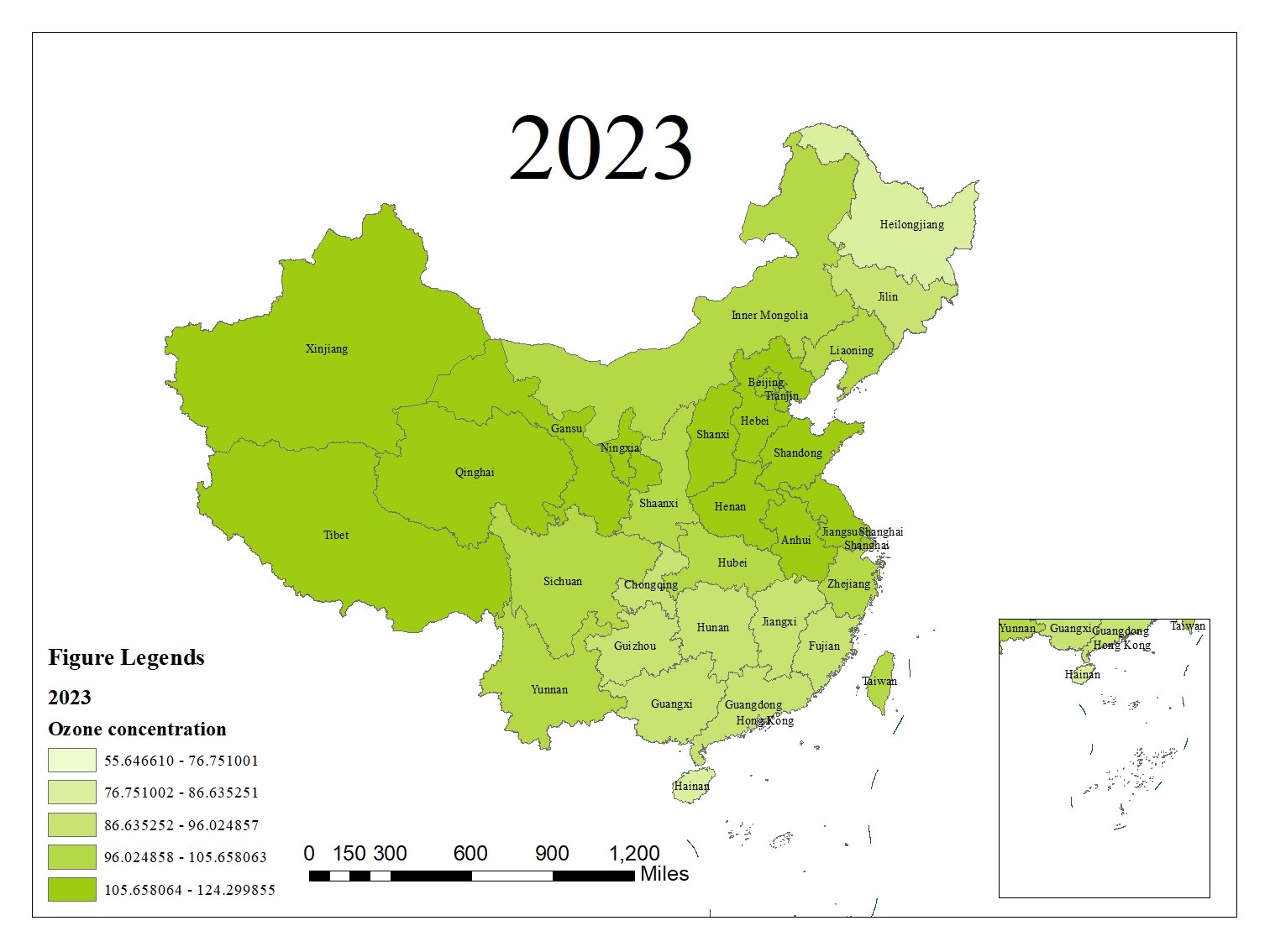

Supplement: Supplementary file 3 [file Data_Sheet_3.zip › O3/O3 concentration/2023.jpg]
